# The mevalonate pathway of isoprenoid biosynthesis supports metabolic flexibility in *Mycobacterium marinum*

**DOI:** 10.1128/jb.00287-25

**Published:** 2025-10-30

**Authors:** Christine M. Qabar, Edward E.K. Baidoo, Emine Akyuz Turumtay, Tariq M. Qayum, Jay D. Keasling, Cressida A. Madigan, Daniel A. Portnoy, Jeffery S. Cox

**Affiliations:** 1 Department of Plant and Microbial Biology, University of California Berkeley 1438, Berkeley, California, USA; 2 Joint BioEnergy Institute, Lawrence Berkeley National Laboratory 1666, Emeryville, California, USA; 3 Biological Systems and Engineering Division, Lawrence Berkeley National Laboratory 1666, Berkeley, California, USA; 4 Department of Molecular Biology, University of California San Diego 8784, La Jolla, California, USA; 5 Department of Bioengineering, University of California Berkeley 1438, Berkeley, California, USA; 6 Department of Chemical and Biomolecular Engineering, University of California Berkeley 1438, Berkeley, California, USA; 7 Department of Molecular and Cellular Biology, University of California Berkeley 1438, Berkeley, California, USA; University of Notre Dame, Notre Dame, Indiana, USA

**Keywords:** mycobacteria, *Mycobacterium marinum*, diversity, metabolism, terpene, terpenoid, mevalonate, methylerithritol, isoprenoid

## Abstract

Isoprenoids are a diverse class of natural products that are essential in all domains of life. Most bacteria synthesize isoprenoids through either the methylerythritol phosphate (MEP) pathway or the mevalonate (MEV) pathway, while a small subset encodes both pathways, including the pathogen *Mycobacterium marinum* (Mm). It is unclear whether the MEV pathway is functional in Mm, or why Mm encodes seemingly redundant metabolic pathways. Here, we show that the MEP pathway is essential in Mm, while the MEV pathway is dispensable in culture, with the ΔMEV mutant having no growth defect in axenic culture but a competitive growth defect compared to WT Mm. We found that the MEV pathway does not play a role in *ex vivo* or *in vivo* acute infection but does play a role in survival of peroxide stress. Metabolite profiling revealed that modulation of the MEV pathway causes compensatory changes in the concentration of MEP intermediates DOXP and CDP-ME, suggesting that the MEV pathway is functional and that the pathways interact at the metabolic level. Finally, the MEV pathway is upregulated early in the shift down to hypoxia, suggesting that it may provide metabolic flexibility to this bacterium. Interestingly, we found that our complemented strains, which vary in copy number of the polyprenyl synthetase *idsB2*, responded differently to peroxide and UV stresses, suggesting a role for this gene as a determinant of downstream prenyl phosphate metabolism. Together, these findings suggest that MEV may serve as an anaplerotic pathway to make isoprenoids under stress conditions.

**IMPORTANCE:**

Organisms from all domains of life utilize isoprenoids to carry out thousands of critical and auxiliary cellular processes, including signaling, maintaining membrane integrity, stress response, and host-pathogen interactions. The common precursor of all isoprenoids is synthesized via one of two biosynthetic pathways. Importantly, some bacteria encode both pathways, including *M. marinum*. We found that only one pathway is essential in *M. marinum*, while the nonessential pathway may confer metabolic flexibility to help the bacterium better adapt to various environmental conditions. We also found that the polyprenyl synthetase IdsB2 plays an important role in driving such phenotypes. Further, we demonstrate metabolic interplay between both functional pathways. These insights represent the first characterization of isoprenoid biosynthesis in dual pathway-encoding mycobacteria.

## INTRODUCTION

Isoprenoids are the largest class of natural products, of which 95,000 have been identified to date (1, 2). In bacteria, isoprenoids play diverse and critical roles, including microbial signaling, stress response, electron transport, and construction of the cell wall (3). Despite wide structural and functional diversity, all isoprenoids arise from the fundamental precursor isopentenyl pyrophosphate (IPP) and its isomer dimethylallyl pyrophosphate (DMAPP). These terpenoid building blocks can be synthesized via two independent pathways: the mevalonate (MEV) pathway, which is primarily found in eukaryotes and archaea, and the methylerythritol phosphate (MEP) pathway, which is found in bacteria, plastids, and algae (4). Downstream of IPP are a series of condensation reactions shared by both the MEV and MEP pathways, collectively known as prenyl phosphate metabolism.

**Fig 1:**
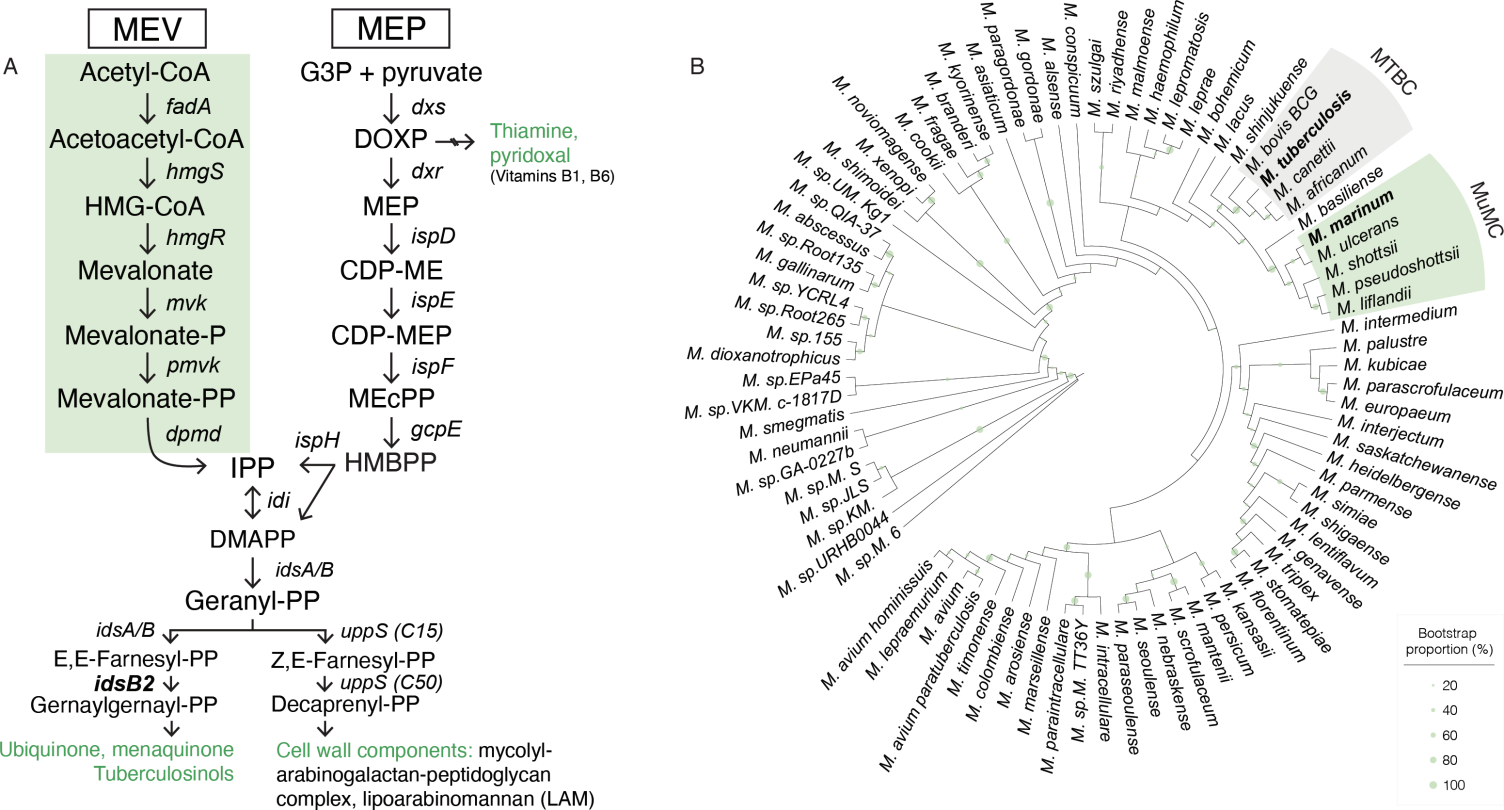
Mm encodes two pathways for isoprenoid biosynthesis. (**A**) Two pathways of isoprenoid biosynthesis. Diagram of the MEV and MEP pathways of isoprenoid precursor biosynthesis. Enzymatic steps are indicated by italicized gene names. Important end products in mycobacteria are shown in green. (**B**) MEV distribution in mycobacteria. The *M. ulcerans–M. marinum* (MuMC) clade is the only group of mycobacteria that encodes the MEV pathway. All mycobacteria, including the MuMC clade, encode the MEP pathway. Shading in green indicates species that encode MEV genes. Shading in gray indicates species that belong to the *M. tuberculosis* complex (MTBC). Tree represents 84 mycobacterial species; sequences and alignment data are available in Table S1 and File S1.

While the MEV and MEP pathways both produce IPP, they consume different starting reagents and proceed via unique enzymatic steps which produce distinct intermediates (Fig. 1A). All eukaryotes and archaea use the MEV pathway for isoprenoid biosynthesis, but there is much diversity in pathway utilization among bacteria. While most bacteria exclusively use the MEP pathway, some bacteria use only the MEV pathway, and others yet encode both pathways (5). Some obligate intracellular bacteria, like *Rickettsia parkeri*, lack any endogenous pathway and instead rely on import of host IPP for metabolism (6).

Insights into the origins of these different pathways can be gleaned from comparative genomics. MEP genes are scattered throughout the genomes of MEP-encoding bacteria, and conversely, MEV genes are found within a single operon, leading some to speculate acquisition via horizontal gene transfer (7, 8). Phylogenomic analyses suggest that the punctate distribution of MEV genes among bacteria can be traced back to a cenancestral MEV pathway, and subsequently, some bacterial lineages lost these genes as they evolved the MEP pathway (9). Mycobacteria present a unique divergence from this hypothesis, given that only one clade of mycobacteria encodes both pathways, including the marine pathogen *Mycobacterium marinum* (Mm). The evolutionary pressure promoting retention of the MEV pathway among certain bacteria remains unknown; however, horizontal gene transfer is not likely for Mm, as the GC content of the MEV operon is 64.56% compared to 65.73% for the rest of the genome (10), suggesting that this pathway was not recently acquired.

In bacteria encoding both MEP and MEV genes, there is evidence that these pathways are nonredundant and may support metabolic flexibility to a variety of stressors. Members of the *Actinomycetota* phylum, which also contains the genus *Mycobacterium*, use the MEP pathway early in growth for primary metabolism and later activate the MEV pathway to synthesize secondary metabolites (8). Interestingly, intermediates in the pathways have been implicated as effectors themselves, including the MEP metabolite (E)−4-hydroxy-3-methyl-but-2-enyl pyrophosphate (HMBPP), which is the most potent activator of host Vγ9Vδ2 T cells (11–13). Another compelling line of evidence points to the MEP pathway as an oxidative stress sensor, as the final two enzymes in this pathway contain oxygen-reactive iron-sulfur clusters (14, 15). Further, the MEP pathway intermediate 2-C-methyl-D-erythritol-2,4-cyclopyrophosphate (MEcPP) accumulates in response to oxidative stress in bacteria and acts as a stress signaling molecule in plastidal plants (16, 17), supporting a model in which encoding both pathways may confer resistance to environmental stresses.

One interesting case is in *Listeria monocytogenes* (Lm), a gram-positive intracellular pathogen, which also encodes both pathways. Lm relies on the MEV pathway for metabolism, yet encodes an MEP pathway that is aerobically nonfunctional due to the failure of the final two genes in the pathway (18). The related *Listeria innocua* encodes an incomplete MEP pathway, including all but those same two final MEP genes which are directly downstream of the synthesis of MEcPP (19). Further, a mutant in the MEcPP synthase *ispF* was identified as more sensitive to oxidative stress in Lm (20). Taken together, it is highly likely that the MEP pathway in *Listeria* synthesizes the putative oxygen sensor MEcPP, while the MEV pathway primarily supports isoprenoid metabolism. However, this remains to be explored in Mm, which also encodes both pathways.

The MEP pathway is essential in the important human pathogen *M. tuberculosis*, its vaccine strain *M. bovis* BCG, and likely all mycobacteria that exclusively use the MEP pathway; however, it is unclear if one or both pathways is essential in Mm (21, 22). Previous transposon mutagenesis experiments failed to identify several genes in the MEP pathway as essential in Mm (23, 24). In contrast, we provide conclusive genetic evidence of the essentiality of the MEP pathway in Mm and, conversely, the dispensability of the MEV pathway. Further, in this work, we demonstrate that although the MEV pathway is nonessential, it is intact, functional, and supports the ability of Mm to persist in dynamic environmental conditions.

## MATERIALS AND METHODS

### Bioinformatic analysis

Phylogenetic tree building was done as previously described (25). Briefly, full-length 16S rRNA sequences were collected from one representative genome for each unique species of mycobacteria available on BioCyc (26) and the NCBI Gene database (27), representing 84 mycobacterial species. Geneious Prime (v2024.0.7) was used to perform a MUSCLE alignment (28) and build a PhyML tree (29) with 100 bootstraps. Annotation and tree visualization were performed on iTol (30). Sequences and alignment data are available in Table S1 and File S1. *In silico* SigF binding motif analysis was performed on the region upstream of the MEV operon to identify the consensus SigF binding motif GTTC[N17]GGGTAT between −10 and −200 of the coding region, allowing for two mismatches. One match was found: GTTC[agtagcggttgacgatt]TGGTAG, located −83 bp from the start codon of *idi*. Structural and sequence alignments for IdsB2 homologs in Mm and *M. tuberculosis* were performed using MUSCLE and visualized using FastTree 2.1.11 (31). AlphaFold 3 (32) was used to predict structures, and structural alignment was performed using PyMOL (33).

### Bacterial strains and culture

*Mycobacterium marinum* M (ATCC BAA-535) was routinely grown at 30°C in Middlebrook 7H9 liquid medium or on 7H10 agar (BD Difco) supplemented with 10% OADC (oleic acid-albumin-dextrose-catalase) and 0.2% Tween 80. *E. coli* strain DH5α was grown at 37°C in Luria broth or agar and used for plasmid cloning. When required, the following antibiotics were used: kanamycin (25 µg/mL for Mm, 50 µg/mL for *E. coli*), hygromycin (50 µg/mL for Mm, 150 µg/mL for *E. coli*), zeocin (100 µg/mL for Mm, 50 µg/mL for *E. coli*), and anhydrotetracycline (200 ng/mL for CRISPR, 500 ng/mL for ORBIT).

### Molecular cloning

Primers, oligos, and plasmids used in this study are listed in Tables S2 and S3. Standard electroporation protocols were used for the transformation of plasmids into *E. coli*. Electrocompetent mycobacteria were prepared by washing OD_600_ 0.5–0.8 cultures four times in decreasing volumes of sterile 10% glycerol, resulting in a final resuspension concentrated ~20–25×. Two hundred microliters of electrocompetent cells were combined with 5 µlLof plasmid DNA in a 0.2 cm electroporation cuvette and electroporated with a single pulse at 2.5 kV, 25 µF capacitance, and 1,000 Ω resistance. Transformants were recovered overnight at 30°C in 7H9 medium and plated on selective 7H10 agar containing antibiotics. Three to six clones were randomly selected and verified via PCR.

### ORBIT

Gene replacement was performed via the oligonucleotide-mediated recombineering followed by Bxb1 integrase targeting (ORBIT) system as previously described (34). Targeting oligos were designed such that an *attP* site was flanked on either side by 30 bp arms homologous to the flanking regions of the genomic region. Strains were transformed with the integrase/annealase plasmid pKM444, induced with anhydrotetracycline (ATc), and transformed with both the targeting oligo and “payload plasmid” pKM464 or pKM496. Transformant colonies were screened via colony PCR and sequencing of the insertion junctions. Integrated payload plasmids were cured out of the genome with the excisionase plasmid pKM512. Transformants were induced with ATc, and the plasmid was counter-selected for on 7H10 + 10% sucrose.

### CRISPRi and CRISPRn

Guide RNA sequences were designed using CHOPCHOP (35) and previously characterized PAM sequences (36). Guides were designed to be 21–24 nt with the most PAM-distal nucleotide being an A or G. BsmBI overhangs were added to each spacer to mediate insertion into pLJR965. Gene silencing was performed using the site-specific transcriptional repression system CRISPRi as previously described (36). Briefly, guide oligos were annealed and ligated into the integrating, dCas9-containing pLJR965 vector, and the subsequent plasmid was transformed into mycobacteria. Inducible transcriptional repression was achieved by treatment with 200 ng/ml ATc. To test strains, mid-log cells were OD_600_-matched to 0.3, serially diluted in 7H9, and 3 µL spots were plated on both 7H10 agar supplemented with kanamycin alone (Kan25 µg/mL; no induction) or kanamycin and ATc (ATc200 ng/mL; induced). Gene deletion was performed using the site-specific gene disruption system CRISPRn as previously described (37). Briefly, up to two guide oligos were cloned into the Cas9-containing vector pCRISPRx, and the subsequent plasmid was transformed into mycobacteria. Cells were then allowed to recover overnight in 7H9. Transformants were then induced with 75 ng/mL ATc for one hour and plated on selective media. Subsequent transformants were screened via sequencing to identify successful mutants. Indels were most common, but deletions were prioritized.

### RNA isolation + RT-PCR

Twenty-five milliliters of OD_600_ 0.3–0.4 cultures were resuspended in 1 mL TRIzol (Invitrogen, catalog #15596026) and lysed via bead-beating thrice in 30-second increments, with incubation on ice in between bursts. RNA was then extracted from the supernatant via chloroform-ethanol extraction, washed using PureLink RNA Mini Wash Buffers I and II (Invitrogen, catalog #12183018A), and treated with DNAse I (NEB). Following a 10-minute DNAse inactivation at 75°C, RNA was stored at −80°C. Primers were designed with the following criteria: 17–25 bp in length, 3′ G/C clamp, 75–150 bp amplicon, Tm ~60°C, and analyzed for secondary structure. cDNA was prepared using a SuperScript III First-Strand Synthesis Kit (Invitrogen, catalog #18080051), and SsoAdvanced Universal SYBR Green Supermix (BioRad, catalog #1725271) was used for RT-PCR reactions on genes of interest as well as 16S for normalization. 1:10-diluted cDNA samples were run in technical triplicate on a CFX Connect Real-Time System (BioRad) running CFX Manager software. Normalized expression ratios were obtained via the 2^-ΔΔCq^ (Livak) method (38).

### Plasmid loss frequency analysis

Strains were passaged every 48 hours in fresh 7H9 media without antibiotic selection, serially diluted, and plated on both plain and hygromycin-containing 7H10 agar. After seven days incubation at 30°C, dilutions were counted, and CFU/ml was calculated for each dilution. Percent loss is calculated as [(CFU/mL on plain 7H10) − CFU/mL on hyg50)]/(CFU/mL on plain 7H10).

### Growth curve

Cultures were grown to mid-log (OD_600_ 0.5–0.8) and back-diluted to 0.05 in 7H9. One hundred microliters of each strain were loaded to respective wells of 96-well flat-bottom microplate (ThermoFisher, catalog #267427). To prevent evaporation of the sample wells, all remaining wells were filled with uninoculated 7H9, and the exterior troughs were filled with 1% agarose. Cultures were shaken at 30°C, and optical density (OD_600_) readings were taken every two hours for eight days on a BioTek Epoch 2 Microplate Spectrophotometer (Agilent) running Gen5 software (v3.11). Readings were normalized to blank 7H9 controls and OD-corrected. Data points acquired during logarithmic growth (determined to be between 38 and 54 hours) were fit with a linear regression, giving the equation y = *k*X + logY_0_. The rate constant *k* was used to determine the doubling time using the equation *t* = ln^2/k^.

### Metabolite extraction + metabolomic analysis

The metabolite extraction protocol was modified from previously described mycobacterial extraction (39). Briefly, cells were grown to a target OD_600_ of 0.2 (early log), 0.5 (mid log), 1 (late log), or 2 (early stationary). The equivalent of 10 OD_600_ units was pelleted, resuspended in metabolite extraction buffer (2:2:1 methanol:acetonitrile:water), and lysed via bead beating six times in 30-second increments at 4°C. Lysate was then pelleted, and supernatant was fractionated through 3 kDa ultra-centrifugal filter columns (Amicon, catalog #UFC500324). Extracts were held at −80°C until targeted metabolomic analysis. Liquid chromatography–mass spectrometry (LC-MS) analysis of metabolites was performed as previously described (40). Adenylate energy charge (AEC) was calculated using (ATP + 1/2 ADP)/(AMP + ADP + ATP) (41). OD_600_ 0.2, 0.5, and 2 samples were isolated and analyzed in a different experiment from OD_600_ 1 samples. All metabolomics data are available in File S3.

### Competitive co-culture

Cultures were grown to mid-log (OD_600_ 0.5–0.8) and back-diluted to 0.05 in 7H9 a day prior to inoculating the co-cultures to ensure uniform growth stage across strains. Strains were inoculated into co-culture tubes at an individual OD_600_ of 0.04 and either transferred to plastic inkwells for normoxic growth or sealed in tubes with rubber stoppers according to the Wayne model for hypoxic growth. Cultures were shaken at 120 rpm (normoxic) or stirred at 360 rpm (hypoxic) at 30°C for the entire time course. Time points were taken daily from day 0 to day 7 (normoxic) or every other day from day 0 to day 12 (hypoxic) and plated on both kanamycin- or hygromycin-containing plates. CFU from each plate were counted on day 5 after plating. Normalized competitive index was calculated using the formula: [(mutant CFU/mL)/(WT CFU/mL)] × (input CI).

### Growth in spent media

Cultures of either strain individually (axenic) or both strains together (co-culture) were grown to an OD_600_ of ~1.3 (axenic) or ~2.6 (co-culture), pelleted, and the supernatant was sterilized through a 0.22 µm filter, resulting in “spent media.” Prior to inoculating new cultures, spent media was supplemented with 0.002 g/mL dextrose and 0.5% glycerol to replenish critical nutrients. Supplemented spent media was then inoculated at an OD_600_ of ~0.08, and OD_600_ and CFU/mL were measured as described above.

### *Ex vivo* macrophage infection

Murine bone marrow-derived macrophages (BMMs) were cultured in DMEM (Gibco) supplemented with 10% FBS, 10% M-CSF supernatant produced by 3T3-MCSF cells as previously described, 2 mM L-glutamine (Gibco), and 1 mM sodium pyruvate (Gibco). BMMs were plated at a density of 3 × 10^4^ cells per well of a 96-well plate and incubated at 37°C for 24 hours. Cells were then treated with 2 ng/µL IFNγ and incubated at 37°C for 24 hours. On the day of infection, the bacterial inoculum was prepared as follows: mid-log (OD_600_ 0.6–0.8) mycobacterial cultures were washed in 1X D-PBS + 0.01% Tween 80, and an MOI of 1 inoculum was prepared in BMM media supplemented with 10% horse serum for opsonization. BMMs were then spin-infected by the opsonized bacteria and incubated at 37°C for 10 minutes to allow for phagocytosis. Following phagocytosis, the supernatant was removed and replaced with fresh BMM media. Plates were incubated at 33°C with daily media changes. At each time point, plates were fixed and washed thrice in 1X D-PBS. Cells were stained with 1 µg/mL DAPI (Invitrogen, catalog #D1306) immediately before imaging on the Opera Phenix High-Content Screening System. Images were analyzed using Harmony high-content imaging and analysis software to quantify the fluorescent area of bacteria (mCherry) and the number of nuclei (DAPI).

### Zebrafish infection

Zebrafish husbandry and experiments were performed in compliance with the United States National Institutes of Health and approved by the University of California, San Diego Institutional Animal Care and Use Committee. Wild-type AB strain zebrafish larvae were incubated at 28.5°C in fish water as previously described (42). Larvae were anesthetized with 2.8% Syncaine (Syndel, catalog #886862) prior to infection and imaging. Larvae of both sexes were injected in the caudal vein at three days post-fertilization using a glass capillary needle with bacterial suspensions diluted in phenol red to allow visualization of successful injection. To determine input CFU, the same infection inoculum was plated directly onto 7H10 agar supplemented with kanamycin (25 µg/mL). Input CFU was optimally determined to be ~40–60 CFU per larva. To normalize bacterial burden across bacterial strains, larvae were infected with varying inocula of each strain (*n* ≥ 10 larvae per bacterial strain at 1:10 or 1:15 dilutions) and fluorescence pixel count (FPC) analysis (42) was used to inoculum match across bacterial strains. Optimal dilutions were determined to be 1:10 for WT Mm and 1:15 for ΔMEV. At two and three days post-infection, zebrafish were transferred to an optical 96-well plate and imaged on a Nikon Eclipse Ti2 microscope. ImageJ was used to determine FPC and brain dissemination.

### Growth in self-generated hypoxia

Hypoxic growth curves were performed in accordance with the Wayne model of nonreplicating persistence as previously described (43). Briefly, mid-log (OD_600_ 0.50) cultures were back-diluted tenfold and transferred into glass tubes with 8 mm stir bars (Grainger, catalog #401R24) to a headspace ratio of 0.5. Cultures were then sealed with a rubber stopper (ThermoFisher, catalog #FB57875) and incubated at 30°C with stirring. OD_600_ and CFU samples were taken daily for a minimum of 10 days. To avoid reaeration during the time course, one tube was designated per time point.

### UV tolerance assay

Cultures were grown to mid-log (OD_600_ 0.5–0.8) and serially diluted in 7H9 media. Five microliters of each dilution was plated on 7H10 agar and exposed at 0, 10, and 20 mJ of UV in a Stratalinker UV 1800 Crosslinker (Stratagene). CFU were counted following seven days of growth at 30°C.

### Oxidative stress survival assay

For H_2_O_2_ survival experiments, Mm was grown in media supplemented with 10% ADS (albumin-dextrose-saline) rather than OADC which contains catalase. Mm was treated with hydrogen peroxide (H_2_O_2_, ThermoFisher catalog #BP2633500) as previously described (44). Mid-log (OD_600_ 0.6–0.8) bacteria were washed in PBS containing 0.2% Tween 80, back-diluted to OD_600_ 0.1, and treated with 0, 1, or 2.5 mM H_2_O_2_ for six hours prior to serial dilution and plating for CFU. Plates were incubated at 30°C for a minimum of six days before counting CFU.

## RESULTS

### The MEP pathway is essential in Mm

While most mycobacteria encode the MEP pathway exclusively, there exists a unique subset which also encodes MEV genes (Fig. 1A), including the pathogen *Mycobacterium marinum*, although it was previously unknown whether the MEV pathway is functional (10). Further, species of the *M. ulcerans–M. marinum* (MuMC) clade, a cluster of highly related subspecies that evolved from Mm, also encode MEV genes (Fig. 1B) (45–47). However, the *M. ulcerans* MEV pathway is predicted to be nonfunctional due to disruption by mobile genetic elements (48), but Mm appears to encode an intact MEV pathway. All other mycobacteria, including the important human pathogen *M. tuberculosis* and its vaccine strain BCG, only encode the MEP pathway (21, 22).

**Fig 2:**
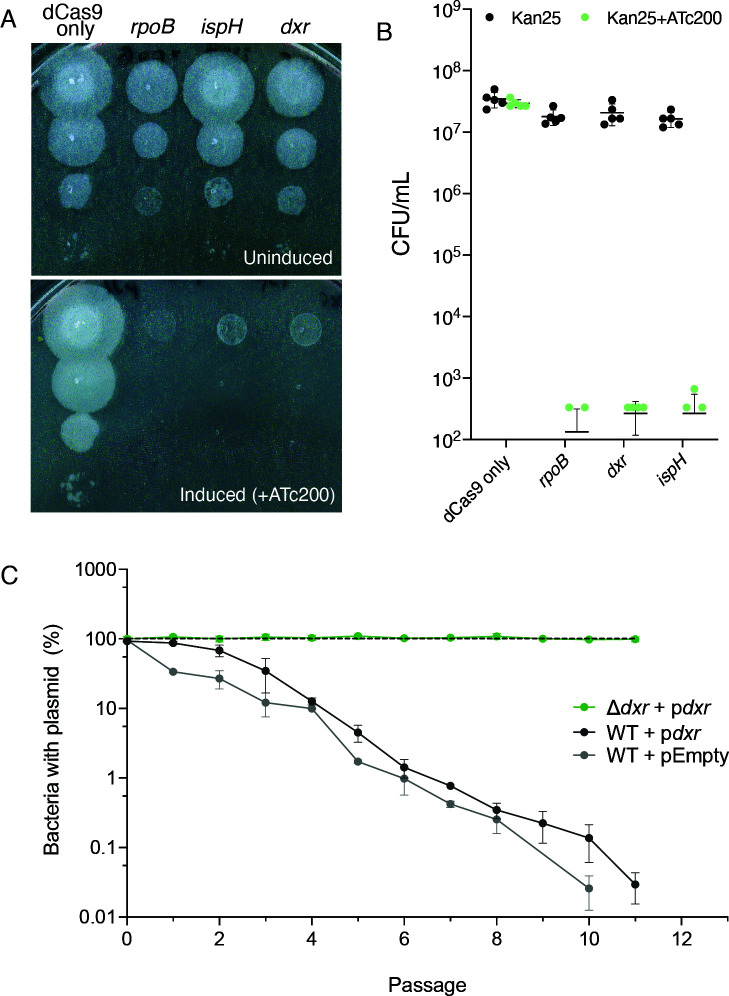
The MEP pathway is essential in Mm. (**A-B**) Transcriptional repression (CRISPRi) of *dxr* and *ispH* is lethal. Guides targeting genes were cloned into the kanamycin-resistant vector pJLR965 as previously described (35, 36). (**A**) Upon induction of CRISPR interference machinery by addition of ATc, target genes are transcriptionally repressed. (**B**) Quantified CFU data from CRISPRi plates ± ATc200. Shown is mean ± SD. *N* = 5 biological replicates. (**C**) Plasmid loss frequency analysis of a Mm mutant in Dxr, the nonmevalonate (MEP) pathway of isoprenoid biosynthesis. MmΔ*dxr* + p*dxr*, WT Mm + p*dxr* , and WT Mm + pEmpty (empty vector) were passaged every 48 hours in fresh 7H9 media without antibiotics. At each passage, cultures were serially diluted and plated on both plain and hygromycin-containing agar. Loss % ≤0 indicates more CFU on hyg. Shown is mean ± SD. *N* = 3 biological replicates.

To test whether the MEP pathway is essential in Mm, we attempted to make knockout strains that lacked either of two genes that encode critical enzymes in the MEP pathway: Dxr, which catalyzes the first committed step in the pathway, and IspH, which catalyzes the final reaction in the pathway (Fig. 1A). We were unable to delete either gene, suggesting that the MEP pathway is essential for growth in Mm, as has been reported for other mycobacteria (21, 22, 49). Using a tetracycline-inducible CRISPR interference (CRISPRi) system to conditionally inhibit expression of these two MEP genes (36), we found that transcriptional repression of *dxr* or *ispH* inhibited growth (Fig. 2A and B), indicating that the MEP pathway is likely essential in Mm.

Since we were unable to delete *dxr* in the wild-type background, we generated a merodiploid strain in which a second copy of *dxr* was expressed ectopically from an episomal plasmid (Δ*dxr*+ p*dxr*). Using this recombinant strain, we were able to delete the chromosomal copy of *dxr*. To definitively test whether *dxr* is essential for growth, we performed plasmid loss experiments with the merodiploid strain. Because the plasmid vector is not faithfully transmitted to both daughter cells during every bacterial cell division (50), growth in the absence of selective pressure to maintain the plasmid will lead to progressive plasmid loss in a population. Strains were serially passaged in nonselective media over 50 generations and plated for CFU to assess plasmid loss. WT cells carrying p*dxr* were able to lose the plasmid and survive, indicating that the p*dxr* plasmid is not essential in this background (Fig. 2C). In contrast, Δ*dxr* + p*dxr* had no apparent plasmid loss over the course of the experiment (Fig. 2C), demonstrating that the *dxr* cargo on the plasmid is essential in the Δ*dxr* background. Together with our inability to delete *dxr* and *ispH* in wild-type Mm (data not shown), these data demonstrate that the MEP pathway is essential in Mm.

Finally, in agreement with findings in *M. tuberculosis*, we found that the Mm genome contains two nonredundant isoforms of *ispH*, also referred to in the literature as *lytB*. We were able to delete *lytB1*/MMAR_0227 (data not shown), which is homologous to the nonessential isoform in *M. tuberculosis* (51), while targeting *ispH*/MMAR_4355, which is homologous to the essential *lytB2* in *M. tuberculosis*, was lethal (Fig. 2A). Together, we demonstrate that *ispH* and *lytB1* are nonredundant in Mm.

### The MEV pathway is nonessential in Mm

**Fig 3:**
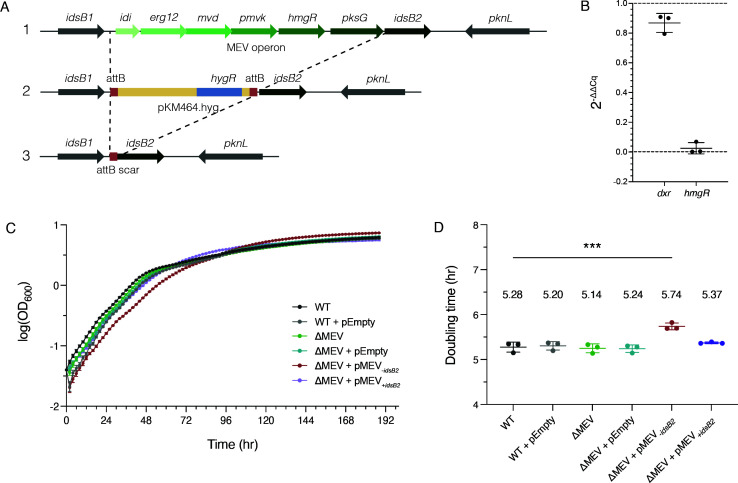
The MEV pathway is nonessential in Mm. (**A**) Deleting the mevalonate pathway of isoprenoid biosynthesis. The MEV locus in Mm (step 1) was replaced with a plasmid containing a hygromycin resistance marker via homologous recombination (step 2). The gene *idsB2* was not deleted to avoid disruptions of isoprenoid synthesis downstream of IPP production. To generate a markerless deletion strain, the integrated plasmid was excised from the locus via induction of a phage excisionase enzyme (step 3). Gene numbers are as follows: *MMAR_3218/idi*, *MMAR_3217/erg12*, *MMAR_3216/mvd*, *MMAR_3215/pmvk*, *MMAR_3214_hmgR*, *MMAR_3213/pksG*, *and MMAR_3212/idsB2*. (**B**) Relative expression of mevalonate (MEV) and non-mevalonate (MEP) pathway genes. Data shown are 2^-ΔΔCq^ of ΔMEV compared to WT. Sample quantification cycle (Cq) values were normalized to respective 16S controls and ΔΔCq values were calculated for representative genes in each pathway (*dxr* for MEP, *hmgR* for MEV) in the ΔMEV strain compared to WT. Shown is mean ± SD. *N* = 3 biological replicates. (**C,D**) MEV deletion mutant has similar growth kinetics as WT, and complementation without *idsB2* impairs growth. MmΔMEV has similar growth kinetics (**C**) and doubling time (**D**) to WT Mm, demonstrating that the MEV pathway does not support standard growth in culture. The complemented strain lacking *idsB2* (ΔMEV + pMEV_-_*_idsB2_*) has a significantly slower growth rate (**D**) compared to the other strains (***P = 0.0001; one-way ANOVA and Dunnett’s multiple comparisons). pEmpty indicates empty vector control. Shown is mean ± SD. *N* = 3 biological replicates.

Unlike the genes of the MEP pathway, the entire MEV pathway is encoded in one operon (Fig. 3A). All six genes in the operon encode proteins that are clear homologs to known enzymes in the MEV pathway, and in particular, the rate-limiting enzyme HmgR shows sequence-level homology to known functional enzymes (Table S4 and File S2). Of note, *idsB2* is the last gene in the MEV operon and catalyzes an enzymatic step downstream of IPP production that is shared by both the MEP and MEV biosynthetic pathways (Fig. 1A). To test whether the MEV pathway is essential in Mm, we attempted to delete only the genes unique to the MEV pathway using oligonucleotide-mediated recombineering, followed by Bxb1 integrase targeting (ORBIT; Fig. 3A) (34). We successfully generated a knockout strain in which the MEV operon is deleted and then “cured” the knockout strain to avoid polar effects from the integrated ORBIT plasmid (Fig. S1A). The successful deletion of the MEV operon, hereby referred to as ΔMEV, demonstrates that the MEV pathway is not essential in Mm under standard culture conditions. We then measured the expression of *hmgR* and *dxr*, which represent the rate-limiting step of the MEV pathway and the first committed step in the MEP pathway, respectively. We observed that ΔMEV and WT had similar levels of *dxr* expression and confirmed that ΔMEV does not express *hmgR* (Fig. 3B). Importantly, this also demonstrates that the MEV operon is transcriptionally active in WT Mm.

To avoid disrupting global isoprenoid metabolism, we deleted the entire MEV operon, except for *idsB2*, which acts downstream of both pathways. Instead, the chromosomal *idsB2* was left intact, and we generated two complementation strains (ΔMEV + pMEV_+_*_idsB2_* and ΔMEV + pMEV_-_*_idsB2_*). We found that both ΔMEV and ΔMEV + pMEV_+_*_idsB2_* displayed WT growth kinetics with no significant difference in doubling time (Fig. 3C and D). However, ΔMEV + pMEV_-_*_idsB2_* had a longer doubling time and elongated log phase compared to all the other strains (Fig. 3C and D). Further, ΔMEV + pMEV_-_*_idsB2_* also formed smaller, pinpoint colonies that required 24–48 hours of extra incubation to become clearly visible (Fig. S1B). Of note, *idi* is a gene shared by both pathways, and the copy located in the MEV operon was deleted in these strains. However, Mm encodes an additional copy of *idi*. Thus, we reasoned that deleting the MEV-linked copy of *idi* would not affect the *idi* at the other chromosomal locus.

### Both pathways are functional and interact at the metabolic level

**Fig 4:**
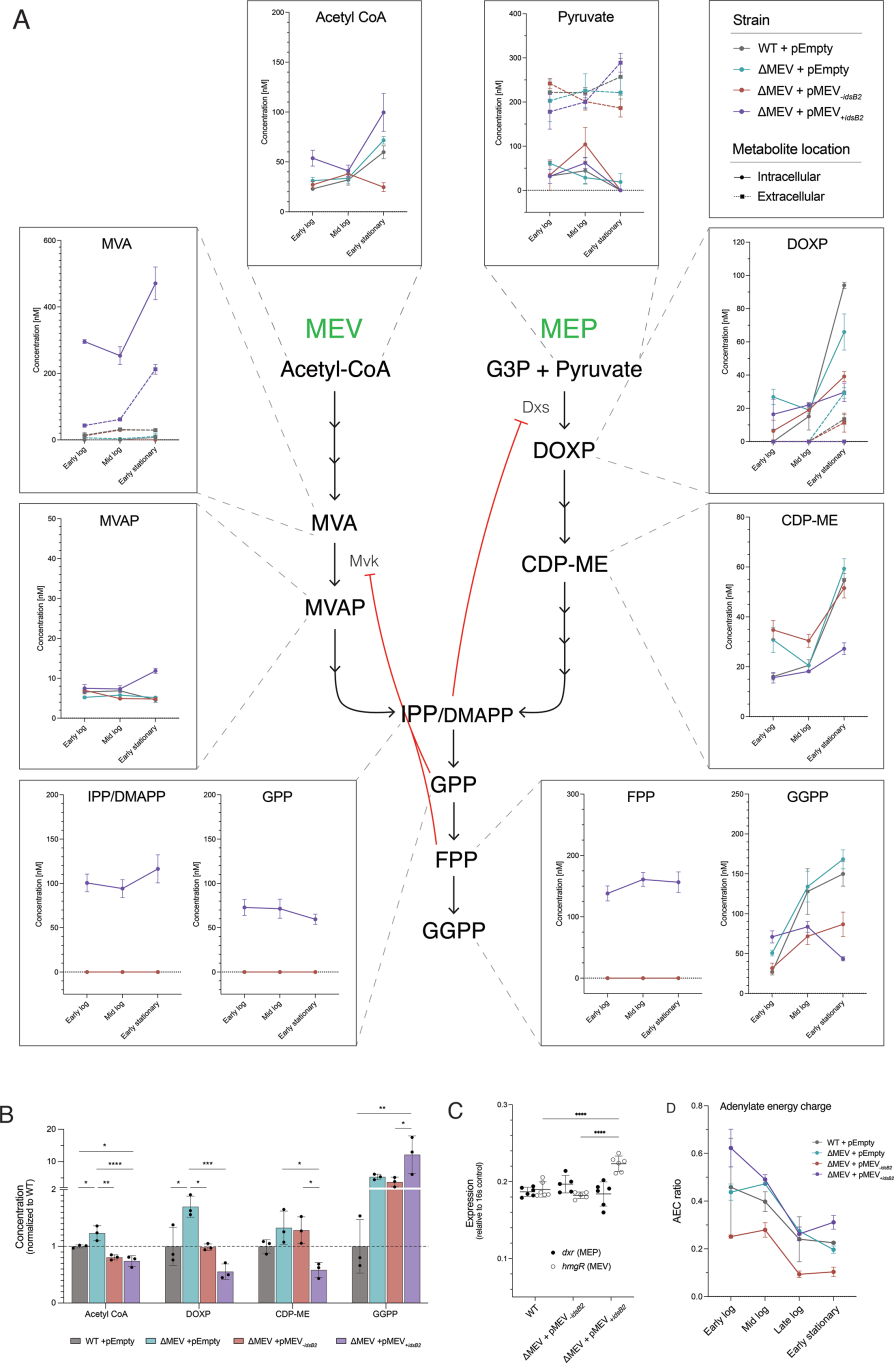
Modulating either isoprenoid biosynthesis pathway impacts metabolism in the other. (**A**) Metabolic flux of both isoprenoid biosynthesis pathways. Shown are starting reagents and intermediates of the MEV pathway, the MEP pathway, and polyprenyl metabolism downstream of both pathways at early logarithmic, mid logarithmic, or early stationary phase (OD_600_ 0.2, 0.5, and 2, respectively). Solid lines indicate intracellular metabolites; dotted lines indicate extracellular metabolites. Shown is mean ± SEM. *N* = 3 biological replicates per strain. (**B**) MEV expression impacts relative quantities of key metabolites. ΔMEV has significantly higher levels of acetyl-CoA, DOXP, and CDP-ME compared to WT, while ΔMEV + pMEV_+_*_idsB2_* has significantly lower levels of these metabolites. Dotted line indicates WT levels. Strains were grown to late logarithmic phase (OD_600_ 1) prior to analysis. Data were analyzed via one-way ANOVA followed by Tukey’s multiple comparisons. *P ≤ 0.05; **P ≤ 0.01; ***P ≤ 0.001; ****P ≤ 0.0001. Shown is mean ± SD. *N* = 3 biological replicates per strain. (**C**) Relative expression of *dxr* and *hmgR* in complemented strains. ΔMEV + pMEV_+_*_idsB2_* had significantly elevated expression of *hmgR*, the rate-limiting step of the MEV pathway (open circles; *P* ≤ 0.0001, two-way ANOVA + Dunnett’s multiple comparisons). Both of the complemented strains had WT expression of *dxr*, representing the MEP pathway (closed circles; *P* = n.s.). Data shown are mean relative to 16S mRNA ± SD. *N* ≥ 5 per strain. (**D**) Adenylate energy charge (AEC) ratio to evaluate cellular energetics. The AEC ratio is significantly lower in ΔMEV + pMEV_-_*_idsB2_* compared to all other strains, reflecting a low energy state in this strain across growth phases. Shown is mean ± SD. *N* = 3 biological replicates per strain.

The ability to delete MEV genes with no obvious defect prompted us to further interrogate the functionality of the MEV pathway. To directly measure intermediates and assess the contribution of the MEV pathway to prenyl phosphate metabolism, we performed metabolic profiling across growth phases (Fig. 4A; Fig. S2A). We observed that the ΔMEV deletion strain had significantly higher levels of acetyl-CoA compared to WT and both complementation strains, all of which encode the MEV pathway (Fig. 4B). Together, these data strongly support that the MEV pathway is functional and drawing on the cellular acetyl-CoA pool.

We detected significant accumulation of mevalonate (MVA) and mevalonate-5-phosphate (MVAP) in ΔMEV + pMEV_+_*_idsB2_* (Fig. 4A), suggesting that this strain has dysregulated flux through the MEV pathway. We did not detect MEV metabolites in the other strains, likely because intermediates of isoprenoid precursor biosynthesis and downstream prenyl pyrophosphate metabolism are normally maintained at very low levels in the cell due to efficient turnover. This dysregulation is also reflected in the expression of *hmgR*, which is significantly elevated in ΔMEV + pMEV_+_*_idsB2_* compared to either ΔMEV + pMEV_-_*_idsB2_* or WT (Fig. 4C). This strain also had significantly lower levels of acetyl-CoA compared to WT (Fig. 4B), demonstrating that MEV overexpression depletes starting substrates of the pathway.

Intracellular levels of isoprenoid intermediates are tightly regulated in both pathways (16, 52–54); thus, our manipulation of the MEV pathway may impact isoprenoid flux via feedback regulation. We measured polyprenyl intermediates downstream of both the MEV and MEP pathways and found that only ΔMEV + pMEV_+_*_idsB2_* had detectable levels of IPP/DMAPP, geranyl pyrophosphate (GPP), and farnesyl pyrophosphate (FPP) (Fig. 4A), further demonstrating dysregulated isoprenoid flux in this strain. Accumulation of FPP and GPP is known to feedback inhibit the enzyme mevalonate kinase (Mvk) (55–57), which is consistent with our observation of significantly lower levels of MVAP compared to MVA in the immediately preceding step (Fig. 4A), although the concomitant increase in GPP and FPP remains unexplained. While geranylgeranyl diphosphate (GGPP) was detected in all strains, it was highest in ΔMEV + pMEV_+_*_idsB2_* (Fig. 4B), supporting a role for *idsB2* in catalyzing the formation of GGPP.

Our metabolic profiling also demonstrated interplay between the MEV and MEP pathways. The ΔMEV mutant had elevated levels of MEP intermediates, particularly of 1-deoxy-D-xylulose-5-phosphate (DOXP; Fig. 4B), while ΔMEV + pMEV_+_*_idsB2_* had lower levels of both DOXP and 4-(cytidine 5′-diphospho)-2-C-methyl-erythritol (CDP-ME; Fig. 4B). This suggests a “seesaw” effect of modulating either pathway. A deletion in the MEV pathway causes a compensatory increase in MEP flux, and elevated MEV flux suppresses the MEP pathway.

To assess cellular energetics in our strains, we determined the adenylate energy charge (AEC) ratio, which measures cellular concentrations of ATP, ADP, and AMP to assess the energy stored in the cellular adenine nucleotide pool (41). In mycobacteria, reported AEC ratios range from 0.4 to 0.8 depending on the species and growth condition (58–60). We found that ΔMEV had a similar AEC ratio to WT and ΔMEV + pMEV_+_*_idsB2_* (Fig. 4D; Fig. S2B), indicating that deleting the MEV pathway does not significantly impact the energetic state of the cell. Interestingly, we found that the AEC of ΔMEV + pMEV*_-idsB2_* was an average twofold lower than WT across growth phases (SD ± 0.489; Fig. 4D), suggesting that the expression of the MEV pathway without *idsB2* induces a low energy state in Mm. Cellular concentrations of dinucleotide cofactors NADH and NADPH were comparable among the strains, indicating similar redox states (Fig. S1C and D). The NADH:NAD + ratio was inversely correlated with AEC ratio in all strains (Fig. 4D; Fig. S1C), likely due to NADH oxidation leading to the synthesis of ATP via the electron transport chain.

Cumulatively, these metabolic findings demonstrate that the MEV pathway is functional and draws from cellular acetyl-CoA pools, and that modulating flux of either pathway has metabolic consequences on the other pathway. Further, the differences observed between the complemented strains support a model in which *idsB2* plays a key role in the fate of prenyl phosphate metabolism.

### ΔMEV has a competitive defect, and complementation with MEV_-_*_idsB2_* provides a competitive advantage

**Fig 5:**
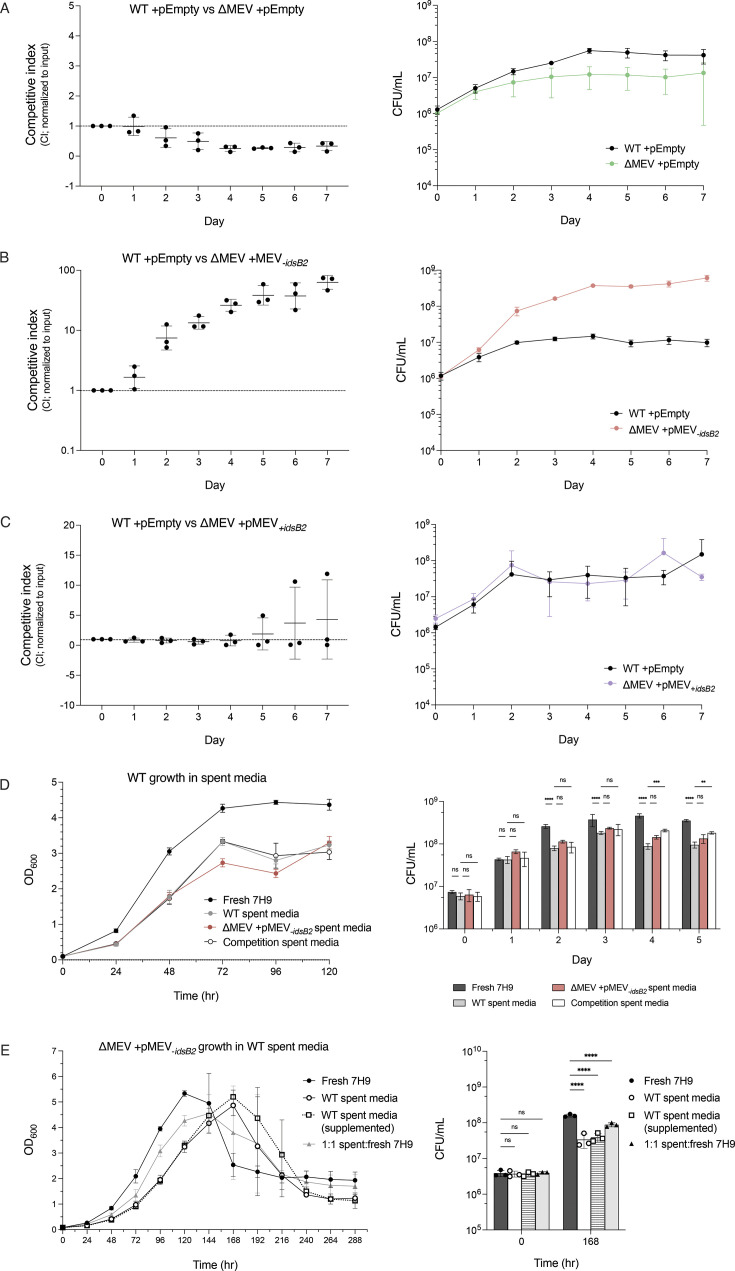
ΔMEV has a competitive defect compared to WT, but ΔMEV + pMEV**_-_***_idsB2_* outcompetes WT. (**A-C**) Competitive phenotypes of all strains compared to WT. WT, ΔMEV, and complemented strains were directly competed, revealing a competitive defect in ΔMEV (**A**) which can be rescued by complementation with pMEV_+_*_idsB2_* (**B**) Complementation with pMEV_-_*_idsB2_* resulted in strains that strongly outcompeted WT (**C**) Competitive index (CI) of 1 indicates strains performed comparably; CI > 1 indicates mutant performed better than WT; CI ≤ 1 indicates WT performed better. pEmpty indicates empty vector control. Shown is mean ± SD. *N* = 3 biological replicates. (**D**) ΔMEV + pMEV**_-_***_idsB2_* does not secrete factors that impair WT growth. There is no significant inhibition of WT growth by spent media from axenic or competition cultures, as measured by OD_600_ (left) or CFU (right). ‘Competition spent media’ indicates spent media isolated from WT vs ΔMEV + pMEV_-_*_idsB2_* competition cultures. **P ≤ 0.01, ****P < 0.0001 (two-way ANOVA + Dunnett’s multiple comparisons). Shown is mean ± SD. *N* = 3 biological replicates. (**E**) WT does not secrete factors that rescue ΔMEV + pMEV**_-_***_idsB2_* growth. There is no significant rescue of the slow growth of ΔMEV + pMEV_-_*_idsB2_* by WT spent media, as measured by OD_600_ (left) and CFU (right). **P ≤ 0.01, ****P < 0.0001 (two-way ANOVA + Sidak’s multiple comparisons). Shown is mean ± SD. *N* = 3 biological replicates.

Given that the MEV pathway is nonessential yet functional, we sought to test conditions in which its deletion may be consequential. Some dual pathway-encoding bacteria employ each pathway differentially in response to stresses which may not be represented in axenic culture, such as oxidative stress and community competition (8, 18). To more sensitively probe the role of the MEV pathway, we directly competed WT and the ΔMEV mutant, which can reveal subtle growth phenotypes that may not be detected in axenic culture. We found that ΔMEV was outcompeted by WT, and that complementation with pMEV_+_*_idsB2_* rescues this defect (Fig. 5A and B). Interestingly, complementation with pMEV_-_*_idsB2_* reverses the competitive defect of ΔMEV, resulting instead in a strong outcompetition of WT by ΔMEV + pMEV*_-idsB2_* (Fig. 5C). This competitive advantage is intriguing because of the longer doubling time and lower AEC ratio of ΔMEV + pMEV_-_*_idsB2_*. These competitive phenotypes remained consistent under hypoxic growth (Fig. S3A through C), suggesting that the presence of *idsB2* in the plasmid, rather than the environmental condition, is responsible for the observed competitive phenotypes.

To address whether WT was restricted in competition by a factor secreted by ΔMEV + pMEV_-_*_idsB2_*, we assessed the growth of WT Mm in spent media from either WT, ΔMEV + pMEV_-_*_idsB2_*, or a co-culture of the two strains. Although WT grew slower in spent compared to fresh media, we found no significant differences in WT growth among the spent media (Fig. 5D), suggesting that ΔMEV + pMEV_-_*_idsB2_* is not secreting a stable factor that restricts the growth of competing bacteria. To test whether ΔMEV + pMEV_-_*_idsB2_* outcompetes due to a stimulatory factor obtained from WT *in trans*, ΔMEV + pMEV_-_*_idsB2_* was inoculated into spent media from WT. We observed no rescue of the slow growth of ΔMEV + pMEV_-_*_idsB2_* by WT spent media (Fig. 5E), supporting that neither WT restriction nor ΔMEV + pMEV_-_*_idsB2_* outcompetition was mediated by a factor in the shared media during competition. Together, these data highlight a potential role for *idsB2* in steering the direction of downstream isoprenoid biosynthesis, the end products of which may influence success in a competitive environment.

### The MEV pathway is dispensable during acute infection

**Fig 6:**
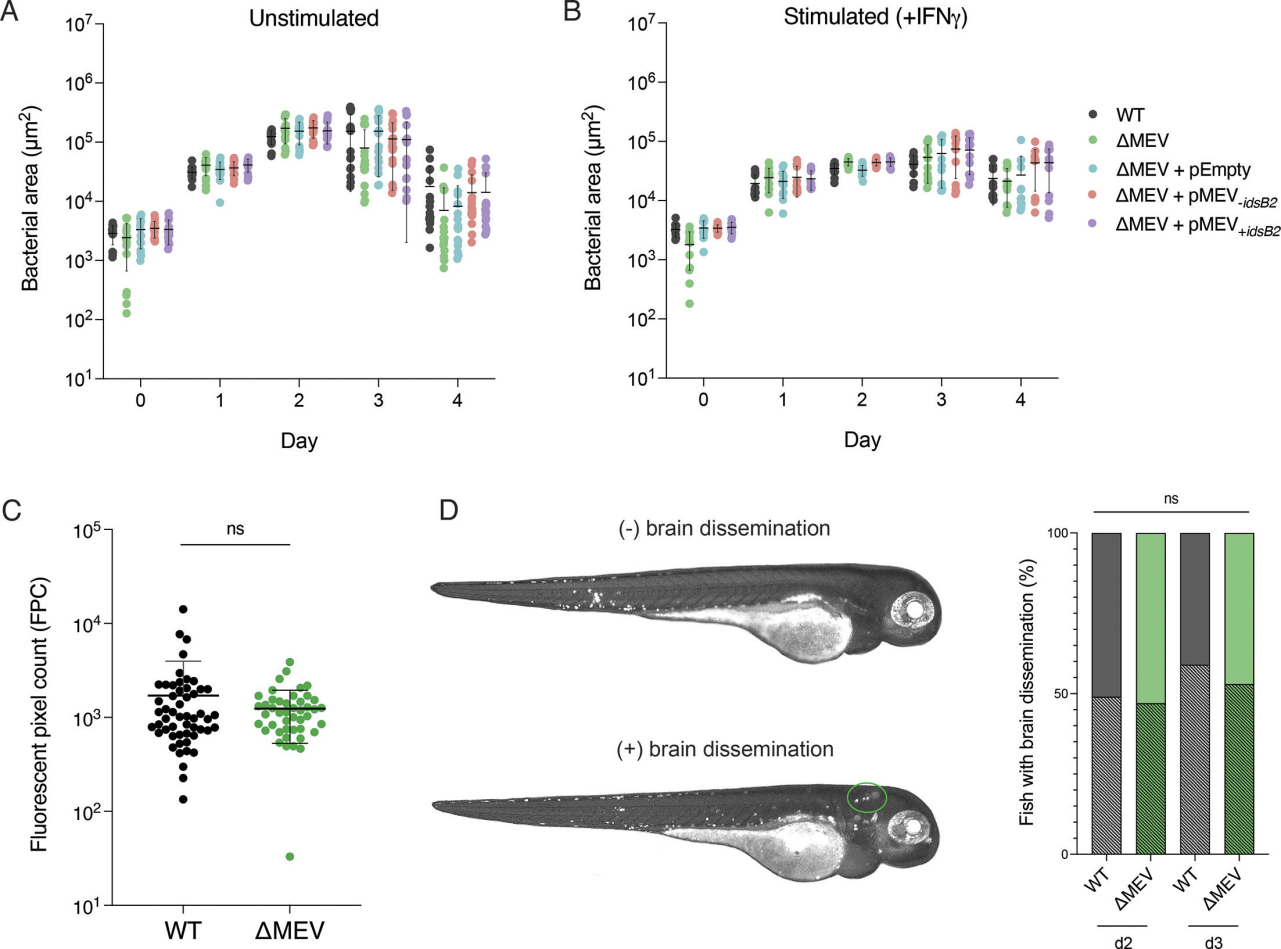
The MEV pathway does not contribute to survival during acute infection. (**A,B**) Growth and survival in macrophages are not significantly different between the strains. Wild-type BMMs were plated and grown with (**A**) or without (**B**) the activating cytokine IFNγ and subsequently infected at an MOI of 1 with various strains of Mm. For four days following infections, cells were fixed and imaged, and the bacterial fluorescence and nuclei count were measured. Data plotted are the mean area of bacterial fluorescence ± SD. *N* = 3 biological replicates. (**C,D**) ΔMEV exhibits WT growth and dissemination to the brain in zebrafish. There is no significant difference in *in vivo* growth (**C**) or dissemination to the brain (**D**) between WT and ΔMEV strains. Larval zebrafish were infected with various strains of Mm via caudal vein injection, and fluorescent pixel count (FPC) was measured on day 2 post-infection. Error bars represent mean FPC ± SD (**C**; *P* = 0.1803; *N* = 3 infections) or percent of disseminated bacteria (**D**; *P* = 0.3471; *N* = 3 infections). FPC comparisons were tested with an unpaired *t*-test and dissemination comparisons were tested with a Fisher’s exact test.

To test whether the MEV pathway plays a role in survival of host immunity, we tested the ΔMEV strain using two widely used models of infection: *ex vivo* macrophage and *in vivo* zebrafish infections (61). We found that survival of Mm in murine bone marrow-derived macrophages (BMMs) is not significantly different among the strains, as the ΔMEV mutant grew with WT kinetics in both resting and interferon gamma (IFNγ)-activated macrophages (Fig. 6A and B). Further, there were no significant differences in macrophage cell death, underscoring the similarity of the strains during infection (Fig. S4A and B). We then turned to the well-established larval zebrafish infection model to assess any strain differences and observed similar *in vivo* replication between WT and ΔMEV (Fig. 6C). Additionally, we found that both strains were able to disseminate to the brain in roughly half of the infections, indicating no overall difference in ability to colonize the host (Fig. 6D). Despite limitations of these models of infection, these data indicate that the MEV pathway is not required for growth in BMMs and dissemination during the acute phase of infection in zebrafish.

### MEV expression has functional consequences under environmental stresses

**Fig 7:**
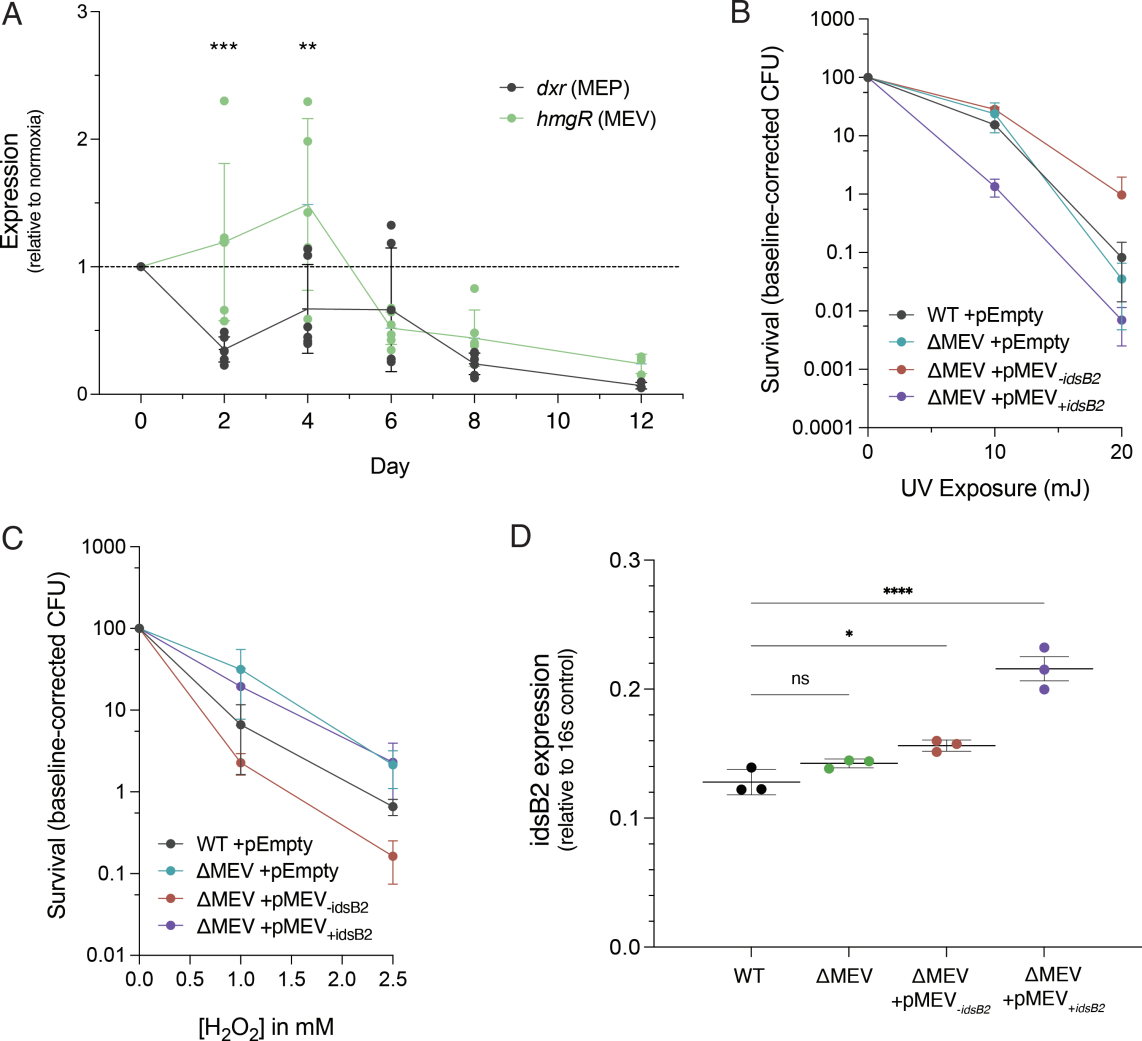
MEV pathway expression has functional consequences under environmental stresses. (**A**) The MEV pathway is significantly upregulated early in the shift down to hypoxia. There is a significant difference in overall pathway expression between time points (*P* = 0.0006; two-way ANOVA). On days 2 and 4 of hypoxia, we detected significantly more *hmgR* transcript compared to *dxr* (*P* = 0.0004 and 0.0010, respectively; Sidak’s multiple comparison). Data were normalized to 16S control, and then normalized expression during hypoxia was compared to normalized expression during hypoxia to generate a ratio. Shown is mean ± SD. *N* = 6 biological replicates. (**B,C**) Functional consequences of modulating MEV. ΔMEV has WT resistance to UV damage (**B**) but is more resistant to H_2_O_2_ (**C**). Complemented strains are differentially sensitive to UV (**B**) and H_2_O_2_ stress (**C**). Strains were grown to mid-log, OD-matched, diluted, and exposed to UV light doses ranging from 0 to 20 mJ (**B**) or to H_2_O_2_ concentrations ranging from 0 to 2.5 mM (**C**). pEmpty indicates empty vector control. Shown is mean ± SD. *N* = 3 biological replicates. (**D**) Relative expression of *idsB2* in all strains. The knockout ΔMEV had WT levels of *idsB2* expression (*P* = n.s.; one-way ANOVA + Tukey’s multiple comparison), while ΔMEV + pMEV_-_*_idsB2_* had slightly elevated expression (*P* = 0.0202), potentially due to the reintroduction of regulatory elements on the plasmid. ΔMEV + pMEV_+_*_idsB2_* had significantly more *idsB2* expression compared to WT (*P* ≤ 0.0001). Data shown are mean relative to 16S mRNA ± SD. *N* = 3 per strain.

Evidence from *Listeria* suggests that the MEP and MEV pathways may be used differently in dual pathway-encoding bacteria based on oxygen availability (18). To address whether this is true in Mm, we used the Wayne model of nonreplicating persistence (43) to induce hypoxia in our strains. We observed no difference in OD_600_ or CFU among the strains throughout hypoxia (Fig. S5A), although we did observe a qualitative difference in oxygen utilization among the strains using the oxygen indicator methylene blue (Fig. S5B), prompting us to more closely assess strain differences under hypoxia. We measured pathway expression during hypoxic adaptation and found significantly more *hmgR* mRNA, the rate-limiting step of the MEV pathway, early in hypoxia compared to *dxr*, particularly on days 2 and 4 (Fig. 7A). Further, published RNAseq data have identified that several MEV genes are upregulated in Mm during hypoxia and shortly after re-aeration, including *pksG*, *hmgR*, *idsB1*, *mpd*, and *erg12* (62, 63). Together, these data suggest that utilization of the MEV pathway may be linked to oxygen availability.

Mm is thought to protect itself from lethal photooxidation by producing β-carotene, a photoinducible isoprenoid that gives Mm its orange pigment (64–66). We observed WT levels of β-carotene in ΔMEV (Fig. S5C), suggesting that the MEV pathway does not play a critical role in its synthesis. However, we found that the complementation strains produced less β-carotene compared to WT and ΔMEV (Fig. S5C), suggesting that multicopy expression of the MEV pathway has a functional consequence on isoprenoid end products. To determine how differing levels of β-carotene may impact resistance to photooxidation from UV exposure, we exposed the strains to varying levels of UV and assayed for survival. We found no differences between WT and ΔMEV, in agreement with our observations of the strains producing equivalent levels of β-carotene; however, the two complemented strains were differentially sensitive to UV damage (Fig. 7B; Fig. S5D). These data suggest a role for *idsB2* copy number in determining the fate of isoprenoids, particularly of the β-carotene pigment.

To test whether the MEV pathway plays a role in survival of oxidative challenge, we exposed our strains to hydrogen peroxide (H_2_O_2_) and assessed survival after six hours. We found that both ΔMEV and ΔMEV + pMEV_+_*_idsB2_* were more resistant to peroxide stress, while ΔMEV + pMEV_-_*_idsB2_* was more sensitive compared to WT (Fig. 7C; Fig. S5E). This might be explained by an increase in the production of the oxidative stress signaling molecule MEcPP in ΔMEV, which has elevated flux through the MEP pathway (Fig. 4). As we observed with UV exposure, the complementation strains were differentially susceptible to H_2_O_2_, suggesting that *idsB2* directs isoprenoid synthesis toward certain end products that may be critical to surviving disparate environmental stresses. We observed that while ΔMEV had WT expression of *idsB2*, ΔMEV + pMEV_+_*_idsB2_* had significantly higher expression compared to WT, demonstrating that *idsB2* on the multicopy plasmid indeed results in more mRNA (Fig. 7D). Taken together, these findings suggest that regulation of the MEV pathway supports flexibility of Mm under variable environmental challenges, including hypoxic, oxidative, and UV stress. Further, it appears that the copy number of *idsB2* is important to direct downstream prenyl phosphate metabolism, and that the observed phenotypes among the strains may be driven by the identity of isoprenoid end products.

## DISCUSSION

In this study, we investigated the MEP and the MEV pathways of isoprenoid biosynthesis to probe the evolutionary pressures that led Mm to encode both. Using a reverse genetics approach, we manipulated each pathway to assess its contribution to growth and physiology, presenting the first study to investigate the functional role of both isoprenoid biosynthesis pathways in Mm. Prior to this study, it was unknown whether these pathways were redundant in Mm, which is one of the few mycobacterial species that encodes both the MEV and MEP pathways. We found that the MEP pathway is essential in Mm, providing critical functional validation of prior high-throughput essentiality experiments, which failed to identify several MEP genes as essential (23, 24). Further, we showed that the MEV pathway is nonessential and used targeted metabolomics to demonstrate that the MEV pathway is functional. Importantly, we found metabolic interplay between the two pathways in Mm.

Not only is MEV pathway usage consequential in certain conditions, but our data further suggest that *idsB2* plays an important role in MEV-derived isoprenoid biosynthesis. We found that the dosage of the *idsB2* gene had functional consequences on our strains with variable MEV flux, which were differentially susceptible to UV- and H_2_O_2_-mediated killing. Further, ΔMEV + pMEV_-_*_idsB2_* has a small colony phenotype and growth defect, consistent with its lower energetic state. IdsB2 is a polyprenyl synthetase whose exact function has not been experimentally demonstrated in Mm, although it is likely involved in the synthesis of a MEV-dependent isoprenoid due to its inclusion in the MEV operon. IdsB2 shares high amino acid (57.06% identity) and structural (0.822 Å root mean square deviation) homology with IdsB in *M. tuberculosis* (Fig. S5F, Table S5), which has been shown to catalyze the condensation of IPP with E,E-FPP to form GGPP (67). Mm encodes four IdsB homologs, likely due to functional diversification. The specific role of IdsB2 in downstream isoprenoid metabolism is not known; however, due to its high sequence and structural homology and its presence in the MEV operon, IdsB2 likely acts downstream of IPP synthesis in Mm. One possible explanation for why *idsB2* is encoded in the MEV operon may be to promote GGPP synthesis from FPP under conditions in which the MEV pathway is upregulated, thus relieving feedback inhibition of Mvk by FPP. Further, the MEV operon may contain *idsB2* to accumulate GGPP and feedback-inhibit the MEP pathway to more fully switch to MEV synthesis under certain conditions, although no feedback inhibition of the MEP pathway by GGPP has been reported to date.

Further, stress conditions may impact substrate availability, which may underlie differential pathway usage in this system. Each pathway uses different starting substrates, and certain stresses may perturb homeostatic metabolism such that using one pathway over the other is favored. A putative sigma factor F (SigF) binding motif can be found in the promoter region of the MEV operon, suggesting that the MEV pathway might indeed play a role in the stress response of Mm by expanding its metabolic repertoire. In support of such a model, SigF has been shown to regulate both carotenoid production and resistance to H_2_O_2_ in *M. smegmatis* (68). While the SigF regulons have been defined in *M. tuberculosis* and *M. smegmatis* (69, 70), these organisms do not encode the MEV pathway, so no functional information is available for the potential SigF regulation of the MEV operon at this time. Regulation of the MEV operon may not only provide a different means to generate IPP/DMAPP under stress but also influence the identity of mature isoprenoids by influencing downstream enzymatic steps. Taken together, these data support a model that the MEV pathway provides metabolic flexibility in Mm.

Evidence from previous bioengineering work further supports the model that the MEV pathway affords Mm metabolic flexibility. Engineering efforts in *E. coli* have demonstrated synergy between the two pathways (71), suggesting that perhaps dual-encoding bacteria like Mm harness this synergy to generate a higher yield of critical isoprenoids than could be achieved by a single pathway. Alternatively, dual-encoding bacteria may use the MEV pathway as a failsafe to ensure isoprenoid flux continues in the case of MEP blockage or prenyl phosphate intermediate accumulation. In addition to the presence of MEV as a metabolic failsafe, our data suggest that *idsB2* may serve as yet another layer of control over end-product fate. Given the divergent metabolism and phenotypes of the two complementation strains, which differ only in the copy number of *idsB2*, it appears that the level of *idsB2* expression may serve as a determinant of the biosynthetic fate of both MEV- and MEP-derived metabolites.

These findings highlight a contrast between the role of the MEV pathway in Mm and other bacteria. Recently, the MEP pathway has emerged as an oxidative stress signaling pathway (16), with striking evidence for this role in *Listeria* species that encode both pathways of isoprenoid biosynthesis. However, these *Listeria* primarily utilize the MEV pathway for IPP synthesis and encode incomplete MEP pathways. In contrast, Mm uses the MEP pathway for essential isoprenoid metabolism and encodes both pathways fully intact. Thus, it appears that these pathways play different roles in *Listeria* and Mm: oxidative stress sensing in *Listeria* and metabolic flexibility in Mm.

Our work provides novel and valuable insights about the interactions between both pathways at the functional and metabolic levels. Our data support that the essential MEP pathway facilitates central metabolism, while the dispensable MEV pathway may represent an inducible accessory system that supports metabolic flexibility in dynamic environmental niches. Further, we demonstrate for the first time that modulation of flux in either pathway causes a compensatory shift in the other, suggesting that the pathways are functional and interact at the metabolic level. Further work is needed to determine the mechanisms of feedback regulation across pathways within a single cell. Such insights are critical to understanding how Mm leverages the MEV pathway and further broaden our ability to engineer its close mycobacterial relatives, such as the important human pathogens *M. tuberculosis* and *M. avium*.

## SUPPLEMENTAL MATERIAL

File S1 jb.00287-25-s0001.rtf Mycobacterial tree alignment.

File S2 jb.00287-25-s0002.rtf HmgR alignment across species.

File S3 jb.00287-25-s0003.xlsx LC-MS metabolomics data.

Supplemental figures jb.00287-25-s0004.pdf Figures S1 to S5.

Tables S1 to S5 jb.00287-25-s0005.xlsx Primers, oligos, plasmids, sequences, and in silico analyses.

ASM does not own the copyrights to Supplemental Material that may be linked to, or accessed through, an article. The authors have granted ASM a non-exclusive, world-wide license to publish the Supplemental Material files. Please contact the corresponding author directly for reuse.
