# Supplementary material for: The mevalonate pathway of isoprenoid biosynthesis supports metabolic flexibility in Mycobacterium marinum
Source: J Bacteriol. 2025 Oct 30;207(11):e00287-25. doi: 10.1128/jb.00287-25 (PMC12632256; doi:10.1128/jb.00287-25)
Supplement: File S1 — Mycobacterial tree alignment. [file jb.00287-25-s0001.rtf]

>M._abscessus Strain ATCC 19977-----------------AGAGTTTGATCCTGGCTCAGGACGAACGCTGGCGGCGTGCTTAACACATGCAAGTCGAACGGAAAGGCCC-T------------T--C----------------GGGGTACTCGAGTGGCGAACGGGTGAGTAACACGTGGGTGATCTGCCCTGCACTCT-GGGATAAGCCTGGGAAACTGGGTCTAATACCGGATAGGACCA-C-ACACTTCATGGTG-A-GTGGTGCAAAG--CTT--T----T----GCGGTGTGGGATGAGCCCGCGGCCTATCAGCTTGTTGGTGGGGTAATGGCCCACCAAGGCGACGACGGGTAGCCGGCCTGAGAGGGTGACCGGCCACACTGGGACTGAGATACGGCCCAGACTCCTACGGGAGGCAGCAGTGGGGAATATTGCACAATGGGCGCAAGCCTGATGCAGCGACGCCGCGTGAGGGATGACGGCCTTCGGGTTGTAAACCTCTTTCAGTAGGGACGAAGCGA----------------AAGTGACGGTACCTACAGAAGAAGGACCGGCCAACTACGTGCCAGCAGCCGCGGTAATACGTAGGGTCCGAGCGTTGTCCGGAATTACTGGGCGTAAAGAGCTCGTAGGTGGTTTGTCGCGTTGTTCGTGAAAACTC-ACAGCTTAACTGTGGGCGTGCGGGCGATACGGGCAGACTAGAGTACTGCAGGGGAGACTGGAATTCCTGGTGTAGCGGTGGAATGCGCAGATATCAGGAGGAACACCGGTGGCGAAGGCGGGTCTCTGGGCAGTAACTGACGCTGAGGAGCGAAAGCGTGGGTAGCGAACAGGATTAGATACCCTGGTAGTCCACGCCGTAAACGGTGGGTACTAGGTGTGGGTTTCCTTCCTT--GGGATCCGTGCCGTAGCTAACGCATTAAGTACCCCGCCTGGGGAGTACGGTCGCAAGACTAAAACTCAAAGGAATTGACGGGGGCCCGCACAAGCGGCGGAGCATGTGGATTAATTCGATGCAACGCGAAGAACCTTACCTGGGTTTGACATGCACAGGACGTACCTAGAGATAGGTATTCCCTTGTGGCCTGTGTGCAGGTGGTGCATGGCTGTCGTCAGCTCGTGTCGTGAGATGTTGGGTTAAGTCCCGCAACGAGCGCAACCCTTGTCCTATGTTGCCAGCGGGTAATGCCGGGGACTCGTAGGAGACTGCCGGGGTCAACTCGGAGGAAGGTGGGGATGACGTCAAGTCATCATGCCCCTTATGTCCAGGGCTTCACACATGCTACAATGGCCAGTACAGAGGGCTGCGAAGCCGTAAGGTGGAGCGAATCCCT--TAAAGCTGGTCTCAGTTCGGATTGGGGTCTGCAACTCGACCCCATGAAGTCGGAGTCGCTAGTAATCGCAGATCAGCAACGCTGCGGTGAATACGTTCCCGGGCCTTGTACACACCGCCCGTCACGTCATGAAAGTCGGTAACACCCGAAGCCAGTGGCCTAACCTT----TTGGAGGGAGCTGTCGAAGGTGGGATCGGCGATTGGGACGAAGTCGTAACAAGGTAGCCGTACCGGAAGGTGCGGCTGGATCACCT------------->M._africanum Strain ATCC 25420-------------------------------------GACGAACGCTGGCGGCGTGCTTAACACATGCAAGTCGAACGGAAAGGTCTCT------------T--C---------------GGAGATACTCGAGTGGCGAACGGGTGAGTAACACGTGGGTGATCTGCCCTGCACTTC-GGGATAAGCCTGGGAAACTGGGTCTAATACCGGATAGGACCA-C-GGGATGCATGTCT-T-GTGGTGGAAAGCGCTT--T----A----GCGGTGTGGGATGAGCCCGCGGCCTATCAGCTTGTTGGTGGGGTGACGGCCTACCAAGGCGACGACGGGTAGCCGGCCTGAGAGGGTGTCCGGCCACACTGGGACTGAGATACGGCCCAGACTCCTACGGGAGGCAGCAGTGGGGAATATTGCACAATGGGCGCAAGCCTGATGCAGCGACGCCGCGTGGGGGATGACGGCCTTCGGGTTGTAAACCTCTTTCACCATCGACGAAGGTCCGG-GTTC--TCTC-GGATTGACGGTAGGTGGAGAAGAAGCACCGGCCAACTACGTGCCAGCAGCCGCGGTAATACGTAGGGTGCGAGCGTTGTCCGGAATTACTGGGCGTAAAGAGCTCGTAGGTGGTTTGTCGCGTTGTTCGTGAAATCTC-ACGGCTTAACTGTGAGCGTGCGGGCGATACGGGCAGACTAGAGTACTGCAGGGGAGACTGGAATTCCTGGTGTAGCGGTGGAATGCGCAGATATCAGGAGGAACACCGGTGGCGAAGGCGGGTCTCTGGGCAGTAACTGACGCTGAGGAGCGAAAGCGTGGGGAGCGAACAGGATTAGATACCCTGGTAGTCCACGCCGTAAACGGTGGGTACTAGGTGTGGGTTTCCTTCCTT--GGGATCCGTGCCGTAGCTAACGCATTAAGTACCCCGCCTGGGGAGTACGGCCGCAAGGCTAAAACTCAAAGGAATTGACGGGGGCCCGCACAAGCGGCGGAGCATGTGGATTAATTCGATGCAACGCGAAGAACCTTACCTGGGTTTGACATGCACAGGACGCGTCTAGAGATAGGCGTTCCCTTGTGGCCTGTGTGCAGGTGGTGCATGGCTGTCGTCAGCTCGTGTCGTGAGATGTTGGGTTAAGTCCCGCAACGAGCGCAACCCTTGTCTCATGTTGCCAGCACGTAATGGTGGGGACTCGTGAGAGACTGCCGGGGTCAACTCGGAGGAAGGTGGGGATGACGTCAAGTCATCATGCCCCTTATGTCCAGGGCTTCACACATGCTACAATGGCCGGTACAAAGGGCTGCGATGCCGCGAGGTTAAGCGAATCCTT--AAAAGCCGGTCTCAGTTCGGATCGGGGTCTGCAACTCGACCCCGTGAAGTCGGAGTCGCTAGTAATCGCAGATCAGCAACGCTGCGGTGAATACGTTCCCGGGCCTTGTACACACCGCCCGTCACGTCATGAAAGTCGGTAACACCCGAAGCCAGTGGCCTAACCCT----CGGGAGGGAGCTGTCGAAG-------------------------------------------------------------------------------->M._alsense----------TGTTTGGAGAGTTTGATCCTGGCTCAGGACGAACGCTGGCGGCGTGCTTAACACATGCAAGTCGAACGGAAAGGCCCCT------------T--C---------------GGGGGTACTCGAGTGGCGAACGGGTGAGTAACACGTGGGTAATCTGCCCTGCACTTC-GGGATAAGCCTGGGAAACTGGGTCTAATACCGAATAGGACCG-C-GAGGCGCATGCCT-T-GTGGTGGAAAG--CTT--T----T----GCGGTGTGGGATGGGCCCGCGGCCTATCAGCTTGTTGGTGGGGTGACGGCCTACCAAGGCGACGACGGGTAGCCGGCCTGAGAGGGTGTCCGGCCACACTGGGACTGAGATACGGCCCAGACTCCTACGGGAGGCAGCAGTGGGGAATATTGCACAATGGGCGCAAGCCTGATGCAGCGACGCCGCGTGGGGGATGACGGCCTTCGGGTTGTAAACCTCTTTCACCATCGACGAAGGTCCGG-GTTT--TCTC-GGATTGACGGTAGGTGGAGAAGAAGCACCGGCCAACTACGTGCCAGCAGCCGCGGTAATACGTAGGGTGCGAGCGTTGTCCGGAATTACTGGGCGTAAAGAGCTCGTAGGTGGTTTGTCGCGTTGTTCGTGAAATCTC-ACGGCTTAACTGTGAGCGTGCGGGCGATACGGGCAGACTGGAGTACTGCAGGGGAGACTGGAATTCCTGGTGTAGCGGTGGAATGCGCAGATATCAGGAGGAACACCGGTGGCGAAGGCGGGTCTCTGGGCAGTAACTGACGCTGAGGAGCGAAAGCGTGGGGAGCGAACAGGATTAGATACCCTGGTAGTCCACGCCGTAAACGGTGGGTACTAGGTGTGGGTTTCCTTCCTT--GGGATCCGTGCCGTAGCTAACGCATTAAGTACCCCGCCTGGGGAGTACGGCCGCAAGGCTAAAACTCAAAGGAATTGACGGGGGCCCGCACAAGCGGCGGAGCATGTGGATTAATTCGATGCAACGCGAAGAACCTTACCTGGGTTTGACATGCACAGGACGCCGGCAGAGATGTCGGTTCCCTTGTGGCCTGTGTGCAGGTGGTGCATGGCTGTCGTCAGCTCGTGTCGTGAGATGTTGGGTTAAGTCCCGCAACGAGCGCAACCCTTGTCTCATGTTGCCAGCGGGTAATGCCGGGGACTCGTGAGAGACTGCCGGGGTCAACTCGGAGGAAGGTGGGGATGACGTCAAGTCATCATGCCCCTTATGTCCAGGGCTTCACACATGCTACAATGGCCGGTACAAAGGGCTGCGATGCCGCAAGGTTAAGCGAATCCTT--TAAAGCCGGTCTCAGTTCGGATCGGGGTCTGCAACTCGACCCCGTGAAGTCGGAGTCGCTAGTAATCGCAGATCAGCAACGCTGCGGTGAATACGTTCCCGGGCCTTGTACACACCGCCCGTCACGTCATGAAAGTCGGTAACACCCGAAGCCAGTGGCCTAACCCTT---TGGGAGGGAGCTGTCGAAGGTGGGATCGGCGATTGGGACGAAGTCGTAACAAGGTAGCCGTACCGGAAGGTGCGGCTGGATCACCTCCTTT-------->M._arosiense----------TGTTTGGAGAGTTTGATCCTGGCTCAGGACGAACGCTGGCGGCGTGCTTAACACATGCAAGTCGAACGGAAAGGCCCCT------------T--C---------------GGGGGTACTCGAGTGGCGAACGGGTGAGTAACACGTGGGCAATCTGCCCTGCACTTC-GGGATAAGCCTGGGAAACTGGGTCTAATACCGGATAGGACCT-T-TAGGCGCATGTCT-A-TTGGTGGAAAG--CTT--T----T----GCGGTGTGGGATGGGCCCGCGGCCTATCAGCTTGTTGGTGGGGTGATGGCCTACCAAGGCGACGACGGGTAGCCGGCCTGAGAGGGTGTCCGGCCACACTGGGACTGAGATACGGCCCAGACTCCTACGGGAGGCAGCAGTGGGGAATATTGCACAATGGGCGCAAGCCTGATGCAGCGACGCCGCGTGGGGGATGACGGCCTTCGGGTTGTAAACCTCTTTCACCATCGACGAAGGTCCGG-GTTT--TCTC-GGATTGACGGTAGGTGGAGAAGAAGCACCGGCCAACTACGTGCCAGCAGCCGCGGTAATACGTAGGGTGCGAGCGTTGTCCGGAATTACTGGGCGTAAAGAGCTCGTAGGTGGTTTGTCGCGTTGTTCGTGAAATCTC-ACGGCTTAACTGTGAGCGTGCGGGCGATACGGGCAGACTAGAGTACTGCAGGGGAGACTGGAATTCCTGGTGTAGCGGTGGAATGCGCAGATATCAGGAGGAACACCGGTGGCGAAGGCGGGTCTCTGGGCAGTAACTGACGCTGAGGAGCGAAAGCGTGGGGAGCGAACAGGATTAGATACCCTGGTAGTCCACGCCGTAAACGGTGGGTACTAGGTGTGGGTTTCCTTCCTT--GGGATCCGTGCCGTAGCTAACGCATTAAGTACCCCGCCTGGGGAGTACGGCCGCAAGGCTAAAACTCAAAGGAATTGACGGGGGCCCGCACAAGCGGCGGAGCATGTGGATTAATTCGATGCAACGCGAAGAACCTTACCTGGGTTTGACATGCACAGGACGCGTCTAGAGATAGGCGTTCCCTTGTGGCCTGTGTGCAGGTGGTGCATGGCTGTCGTCAGCTCGTGTCGTGAGATGTTGGGTTAAGTCCCGCAACGAGCGCAACCCTTGTCTCATGTTGCCAGCGGGTAATGCCGGGGACTCGTGAGAGACTGCCGGGGTCAACTCGGAGGAAGGTGGGGATGACGTCAAGTCATCATGCCCCTTATGTCCAGGGCTTCACACATGCTACAATGGCCGGTACAAAGGGCTGCGATGCCGTAAGGTTAAGCGAATCCTT-TTAAAGCCGGTCTCAGTTCGGATTGGGGTCTGCAACTCGACCCCATGAAGTCGGAGTCGCTAGTAATCGCAGATCAGCAACGCTGCGGTGAATACGTTCCCGGGCCTTGTACACACCGCCCGTCACGTCATGAAAGTCGGTAACACCCGAAGCCAGTGGCCTAACCTT----TTGGAGGGAGCTGTCGAAGGTGGGATCGGCGATTGGGACGAAGTCGTAACAAGGTAGCCGTACCGGAAGGTGCGGCTGGATCACCTCCTTT-------->M._asiaticum---------TTGTTTGGAGAGTTTGATCCTGGCTCAGGACGAACGCTGGCGGCGTGCTTAACACATGCAAGTCGAACGGAAAGGCCCCT------------T--C---------------GGGGGTACTCGAGTGGCGAACGGGTGAGTAACACGTGGGTGATCTGCCCTGCACTTC-GGGATAAGCCTGGGAAACTGGGTCTAATACCGGATAGGACCA-C-GGGATGCATGTCC-T-GTGGTGGAAAG--CTT--T----T----GCGGTGTGGGATGGGCCCGCGGCCTATCAGCTTGTTGGTGGGGTGACGGCCTACCAAGGCGACGACGGGTAGCCGGCCTGAGAGGGTGTCCGGCCACACTGGGACTGAGATACGGCCCAGACTCCTACGGGAGGCAGCAGTGGGGAATATTGCACAATGGGCGCAAGCCTGATGCAGCGACGCCGCGTGGGGGATGACGGCCTTCGGGTTGTAAACCTCTTTCACCATCGACGAAGGTCCGG-GTTT--TCTC-GGATTGACGGTAGGTGGAGAAGAAGCACCGGCCAACTACGTGCCAGCAGCCGCGGTAATACGTAGGGTGCGAGCGTTGTCCGGAATTACTGGGCGTAAAGAGCTCGTAGGTGGTTTGTCGCGTTGTTCGTGAAATCTC-ACGGCTTAACTGTGAGCGTGCGGGCGATACGGGCAGACTGGAGTACTGCAGGGGAGACTGGAATTCCTGGTGTAGCGGTGGAATGCGCAGATATCAGGAGGAACACCGGTGGCGAAGGCGGGTCTCTGGGCAGTAACTGACGCTGAGGAGCGAAAGCGTGGGGAGCGAACAGGATTAGATACCCTGGTAGTCCACGCCGTAAACGGTGGGTACTAGGTGTGGGTTTCCTTCCTT--GGGATCCGTGCCGTAGCTAACGCATTAAGTACCCCGCCTGGGGAGTACGGCCGCAAGGCTAAAACTCAAAGGAATTGACGGGGGCCCGCACAAGCGGCGGAGCATGTGGATTAATTCGATGCAACGCGAAGAACCTTACCTGGGTTTGACATGCACAGGACGCCGGCAGAGATGTCGGTTCCCTTGTGGCCTGTGTGCAGGTGGTGCATGGCTGTCGTCAGCTCGTGTCGTGAGATGTTGGGTTAAGTCCCGCAACGAGCGCAACCCTTGTCTCATGTTGCCAGCGGGTAATGCCGGGGACTCGTGAGAGACTGCCGGGGTCAACTCGGAGGAAGGTGGGGATGACGTCAAGTCATCATGCCCCTTATGTCCAGGGCTTCACACATGCTACAATGGCCGGTACAAAGGGCTGCGATGCCGCGAGGTTAAGCGAATCCTT-TTAAAGCCGGTCTCAGTTCGGATCGGGGTCTGCAACTCGACCCCGTGAAGTCGGAGTCGCTAGTAATCGCAGATCAGCAACGCTGCGGTGAATACGTTCCCGGGCCTTGTACACACCGCCCGTCACGTCATGAAAGTCGGTAACACCCGAAGCCAGTGGCCTAACCCTT---TGGGAGGGAGCTGTCGAAGGTGGGATCGGCGATTGGGACGAAGTCGTAACAAGGTAGCCGTACCGGAAGGTGCGGCTGGATCACCTCCTTTCTAAGGAG>M._avium----------TGTTTGGAGAGTTTGATCCTGGCTCAGGACGAACGCTGGCGGCGTGCTTAACACATGCAAGTCGAACGGAAAGGCCTCT------------T--C---------------GGAGGTACTCGAGTGGCGAACGGGTGAGTAACACGTGGGCAATCTGCCCTGCACTTC-GGGATAAGCCTGGGAAACTGGGTCTAATACCGGATAGGACCT-C-AAGACGCATGTCT-T-CTGGTGGAAAG--CTT--T----T----GCGGTGTGGGATGGGCCCGCGGCCTATCAGCTTGTTGGTGGGGTGACGGCCTACCAAGGCGACGACGGGTAGCCGGCCTGAGAGGGTGTCCGGCCACACTGGGACTGAGATACGGCCCAGACTCCTACGGGAGGCAGCAGTGGGGAATATTGCACAATGGGCGCAAGCCTGATGCAGCGACGCCGCGTGGGGGATGACGGCCTTCGGGTTGTAAACCTCTTTCACCATCGACGAAGGTCCGG-GTTT--TCTC-GGATTGACGGTAGGTGGAGAAGAAGCACCGGCCAACTACGTGCCAGCAGCCGCGGTAATACGTAGGGTGCGAGCGTTGTCCGGAATTACTGGGCGTAAAGAGCTCGTAGGTGGTTTGTCGCGTTGTTCGTGAAATCTC-ACGGCTTAACTGTGAGCGTGCGGGCGATACGGGCAGACTAGAGTACTGCAGGGGAGACTGGAATTCCTGGTGTAGCGGTGGAATGCGCAGATATCAGGAGGAACACCGGTGGCGAAGGCGGGTCTCTGGGCAGTAACTGACGCTGAGGAGCGAAAGCGTGGGGAGCGAACAGGATTAGATACCCTGGTAGTCCACGCCGTAAACGGTGGGTACTAGGTGTGGGTTTCCTTCCTT--GGGATCCGTGCCGTAGCTAACGCATTAAGTACCCCGCCTGGGGAGTACGGCCGCAAGGCTAAAACTCAAAGGAATTGACGGGGGCCCGCACAAGCGGCGGAGCATGTGGATTAATTCGATGCAACGCGAAGAACCTTACCTGGGTTTGACATGCACAGGACGCGTCTAGAGATAGGCGTTCCCTTGTGGCCTGTGTGCAGGTGGTGCATGGCTGTCGTCAGCTCGTGTCGTGAGATGTTGGGTTAAGTCCCGCAACGAGCGCAACCCTTGTCTCATGTTGCCAGCGGGTAATGCCGGGGACTCGTGAGAGACTGCCGGGGTCAACTCGGAGGAAGGTGGGGATGACGTCAAGTCATCATGCCCCTTATGTCCAGGGCTTCACACATGCTACAATGGCCGGTACAAAGGGCTGCGATGCCGTAAGGTTAAGCGAATCCTT-TTAAAGCCGGTCTCAGTTCGGATTGGGGTCTGCAACTCGACCCCATGAAGTCGGAGTCGCTAGTAATCGCAGATCAGCAACGCTGCGGTGAATACGTTCCCGGGCCTTGTACACACCGCCCGTCACGTCATGAAAGTCGGTAACACCCGAAGCCAGTGGCCTAACCCTT--TTGGGAGGGAGCTGTCGAAGGTGGGATCGGCGATTGGGACGAAGTCGTAACAAGGTAGCCGTACCGGAAGGTGCGGCTGGATCACCTCCTTT-------->M._avium_hominissuis---------TTGTTTGGAGAGTTTGATCCTGGCTCAGGACGAACGCTGGCGGCGTGCTTAACACATGCAAGTCGAACGGAAAGGCCTCT------------T--C---------------GGAGGTACTCGAGTGGCGAACGGGTGAGTAACACGTGGGCAATCTGCCCTGCACTTC-GGGATAAGCCTGGGAAACTGGGTCTAATACCGGATAGGACCT-C-AAGACGCATGTCT-T-CTGGTGGAAAG--CTT--T----T----GCGGTGTGGGATGGGCCCGCGGCCTATCAGCTTGTTGGTGGGGTGACGGCCTACCAAGGCGACGACGGGTAGCCGGCCTGAGAGGGTGTCCGGCCACACTGGGACTGAGATACGGCCCAGACTCCTACGGGAGGCAGCAGTGGGGAATATTGCACAATGGGCGCAAGCCTGATGCAGCGACGCCGCGTGGGGGATGACGGCCTTCGGGTTGTAAACCTCTTTCACCATCGACGAAGGTCCGG-GTTT--TCTC-GGATTGACGGTAGGTGGAGAAGAAGCACCGGCCAACTACGTGCCAGCAGCCGCGGTAATACGTAGGGTGCGAGCGTTGTCCGGAATTACTGGGCGTAAAGAGCTCGTAGGTGGTTTGTCGCGTTGTTCGTGAAATCTC-ACGGCTTAACTGTGAGCGTGCGGGCGATACGGGCAGACTAGAGTACTGCAGGGGAGACTGGAATTCCTGGTGTAGCGGTGGAATGCGCAGATATCAGGAGGAACACCGGTGGCGAAGGCGGGTCTCTGGGCAGTAACTGACGCTGAGGAGCGAAAGCGTGGGGAGCGAACAGGATTAGATACCCTGGTAGTCCACGCCGTAAACGGTGGGTACTAGGTGTGGGTTTCCTTCCTT--GGGATCCGTGCCGTAGCTAACGCATTAAGTACCCCGCCTGGGGAGTACGGCCGCAAGGCTAAAACTCAAAGGAATTGACGGGGGCCCGCACAAGCGGCGGAGCATGTGGATTAATTCGATGCAACGCGAAGAACCTTACCTGGGTTTGACATGCACAGGACGCGTCTAGAGATAGGCGTTCCCTTGTGGCCTGTGTGCAGGTGGTGCATGGCTGTCGTCAGCTCGTGTCGTGAGATGTTGGGTTAAGTCCCGCAACGAGCGCAACCCTTGTCTCATGTTGCCAGCGGGTAATGCCGGGGACTCGTGAGAGACTGCCGGGGTCAACTCGGAGGAAGGTGGGGATGACGTCAAGTCATCATGCCCCTTATGTCCAGGGCTTCACACATGCTACAATGGCCGGTACAAAGGGCTGCGATGCCGTAAGGTTAAGCGAATCCTT-TTAAAGCCGGTCTCAGTTCGGATTGGGGTCTGCAACTCGACCCCATGAAGTCGGAGTCGCTAGTAATCGCAGATCAGCAACGCTGCGGTGAATACGTTCCCGGGCCTTGTACACACCGCCCGTCGCGTCATGAAAGTCGGTAACACCCGAAGCCAGTGGCCTAACCCTT--TTGGGAGGGAGCTGTCGAAGGTGGGATCGGCGATTGGGACGAAGTCGTAACAAGGTAGCCGTACCGGAAGGTGCGGCTGGATCACCTCCTTTCTAAGGAG>M._avium_paratuberculosis----------TGTTTGGAGAGTTTGATCCTGGCTCAGGACGAACGCTGGCGGCGTGCTTAACACATGCAAGTCGAACGGAAAGGCCTCT------------T--C---------------GGAGGTACTCGAGTGGCGAACGGGTGAGTAACACGTGGGCAATCTGCCCTGCACTTC-GGGATAAGCCTGGGAAACTGGGTCTAATACCGGATAGGACCT-C-AAGACGCATGTCT-T-CTGGTGGAAAG--CTT--T----T----GCGGTGTGGGATGGGCCCGCGGCCTATCAGCTTGTTGGTGGGGTGACGGCCTACCAAGGCGACGACGGGTAGCCGGCCTGAGAGGGTGTCCGGCCACACTGGGACTGAGATACGGCCCAGACTCCTACGGGAGGCAGCAGTGGGGAATATTGCACAATGGGCGCAAGCCTGATGCAGCGACGCCGCGTGGGGGATGACGGCCTTCGGGTTGTAAACCTCTTTCACCATCGACGAAGGTCCGG-GTTT--TCTC-GGATTGACGGTAGGTGGAGAAGAAGCACCGGCCAACTACGTGCCAGCAGCCGCGGTAATACGTAGGGTGCGAGCGTTGTCCGGAATTACTGGGCGTAAAGAGCTCGTAGGTGGTTTGTCGCGTTGTTCGTGAAATCTC-ACGGCTTAACTGTGAGCGTGCGGGCGATACGGGCAGACTAGAGTACTGCAGGGGAGACTGGAATTCCTGGTGTAGCGGTGGAATGCGCAGATATCAGGAGGAACACCGGTGGCGAAGGCGGGTCTCTGGGCAGTAACTGACGCTGAGGAGCGAAAGCGTGGGGAGCGAACAGGATTAGATACCCTGGTAGTCCACGCCGTAAACGGTGGGTACTAGGTGTGGGTTTCCTTCCTT--GGGATCCGTGCCGTAGCTAACGCATTAAGTACCCCGCCTGGGGAGTACGGCCGCAAGGCTAAAACTCAAAGGAATTGACGGGGGCCCGCACAAGCGGCGGAGCATGTGGATTAATTCGATGCAACGCGAAGAACCTTACCTGGGTTTGACATGCACAGGACGCGTCTAGAGATAGGCGTTCCCTTGTGGCCTGTGTGCAGGTGGTGCATGGCTGTCGTCAGCTCGTGTCGTGAGATGTTGGGTTAAGTCCCGCAACGAGCGCAACCCTTGTCTCATGTTGCCAGCGGGTAATGCCGGGGACTCGTGAGAGACTGCCGGGGTCAACTCGGAGGAAGGTGGGGATGACGTCAAGTCATCATGCCCCTTATGTCCAGGGCTTCACACATGCTACAATGGCCGGTACAAAGGGCTGCGATGCCGTAAGGTTAAGCGAATCCTT-TTAAAGCCGGTCTCAGTTCGGATTGGGGTCTGCAACTCGACCCCATGAAGTCGGAGTCGCTAGTAATCGCAGATCAGCAACGCTGCGGTGAATACGTTCCCGGGCCTTGTACACACCGCCCGTCACGTCATGAAAGTCGGTAACACCCGAAGCCAGTGGCCTAACCCTT--TTGGGAGGGAGCTGTCGAAGGTGGGATCGGCGATTGGGACGAAGTCGTAACAAGGTAGCCGTACCGGAAGGTGCGGCTGGATCACCTCCTTT-------->M._basiliense---------TTGTTTGGAGAGTTTGATCCTGGCTCAGGACGAACGCTGGCGGCGTGCTTAACACATGCAAGTCGAACGGAAAGGTCTCT------------T--C---------------GGAGATACTCGAGTGGCGAACGGGTGAGTAACACGTGGGCGATCTGCCCTGCACTTC-GGGATAAGCCTGGGAAACTGGGTCTAATACCGGATAGGACCA-C-GGGATTCATGTCC-T-GTGGTGGAAAG--CTT--T----T----GCGGTGTGGGATGGGCCCGCGGCCTATCAGCTTGTTGGTGGGGTAACGGCCTACCAAGGCGACGACGGGTAGCCGGCCTGAGAGGGTGTCCGGCCACACTGGGACTGAGATACGGCCCAGACTCCTACGGGAGGCAGCAGTGGGGAATATTGCACAATGGGCGCAAGCCTGATGCAGCGACGCCGCGTGGGGGATGACGGCCTTCGGGTTGTAAACCTCTTTCACCATCGACGAAGGTCCGG-T--T--TCCC-GGATTGACGGTAGGTGGAGAAGAAGCACCGGCCAACTACGTGCCAGCAGCCGCGGTAATACGTAGGGTGCGAGCGTTGTCCGGAATTACTGGGCGTAAAGAGCTCGTAGGTGGTTTGTCGCGTTGTTCGTGAAATCTC-ACGGCTTAACTGTGAGCGTGCGGGCGATACGGGCAGACTAGAGTACTGCAGGGGAGACTGGAATTCCTGGTGTAGCGGTGGAATGCGCAGATATCAGGAGGAACACCGGTGGCGAAGGCGGGTCTCTGGGCAGTAACTGACGCTGAGGAGCGAAAGCGTGGGGAGCGAACAGGATTAGATACCCTGGTAGTCCACGCCGTAAACGGTGGGTACTAGGTGTGGGTTTCCTTCCTT--GGGATCCGTGCCGTAGCTAACGCATTAAGTACCCCGCCTGGGGAGTACGGCCGCAAGGCTAAAACTCAAAGGAATTGACGGGGGCCCGCACAAGCGGCGGAGCATGTGGATTAATTCGATGCAACGCGAAGAACCTTACCTGGGTTTGACATGCACAGGACGCGTCTAGAGATAGGCGTTCCCTTGTGGCCTGTGTGCAGGTGGTGCATGGCTGTCGTCAGCTCGTGTCGTGAGATGTTGGGTTAAGTCCCGCAACGAGCGCAACCCTTGTCTCATGTTGCCAGCACGTAATGGTGGGGACTCGTGAGAGACTGCCGGGGTCAACTCGGAGGAAGGTGGGGATGACGTCAAGTCATCATGCCCCTTATGTCCAGGGCTTCACACATGCTACAATGGCCGGTACAAAGGGCTGCGATGCCGCGAGGTTAAGCGAATCCTT-TTAAAGCCGGTCTCAGTTCGGATCGGGGTCTGCAACTCGACCCCGTGAAGTCGGAGTCGCTAGTAATCGCAGATCAGCAACGCTGCGGTGAATACGTTCCCGGGCCTTGTACACACCGCCCGTCACGTCATGAAAGTCGGTAACACCCGAAGCCAGTGGCCTAACCCTT---TGGGAGGGAGCTGTCGAAGGTGGGATCGGCGATTGGGACGAAGTCGTAACAAGGTAGCCGTACCGGAAGGTGCGGCTGGATCACCTCCTTTCTAA---->M._bohemicum----------TGTTTGGAGAGTTTGATCCTGGCTCAGGACGAACGCTGGCGGCGTGCTTAACACATGCAAGTCGAACGGAAAGGTCTCT------------T--C---------------GGAGATACTCGAGTGGCGAACGGGTGAGTAACACGTGGGCAATCTGCCCTGCACTTC-GGGATAAGCCTGGGAAACTGGGTCTAATACCGGATATGACCT-C-GAGGCGCATGCCT-T-GTGGTGGAAAG--CTT--T----T----GCGGTGTGGGATGGGCCCGCGGCCTATCAGCTTGTTGGTGGGGTGATGGCCTACCAAGGCGACGACGGGTAGCCGGCCTGAGAGGGTGTCCGGCCACACTGGGACTGAGATACGGCCCAGACTCCTACGGGAGGCAGCAGTGGGGAATATTGCACAATGGGCGCAAGCCTGATGCAGCGACGCCGCGTGGGGGATGACGGCCTTCGGGTTGTAAACCTCTTTCACCATCGACGAAGGTCCGG-GGTT--TCTC-GGATTGACGGTAGGTGGAGAAGAAGCACCGGCCAACTACGTGCCAGCAGCCGCGGTAATACGTAGGGTGCGAGCGTTGTCCGGAATTACTGGGCGTAAAGAGCTCGTAGGTGGTTTGTCGCGTTGTTCGTGAAATCTC-ACGGCTTAACTGTGAGCGTGCGGGCGATACGGGCAGACTAGAGTACTGCAGGGGAGACTGGAATTCCTGGTGTAGCGGTGGAATGCGCAGATATCAGGAGGAACACCGGTGGCGAAGGCGGGTCTCTGGGCAGTAACTGACGCTGAGGAGCGAAAGCGTGGGGAGCGAACAGGATTAGATACCCTGGTAGTCCACGCCGTAAACGGTGGGTACTAGGTGTGGGTTTCCTTCCTT--GGGATCCGTGCCGTAGCTAACGCATTAAGTACCCCGCCTGGGGAGTACGGCCGCAAGGCTAAAACTCAAAGGAATTGACGGGGGCCCGCACAAGCGGCGGAGCATGTGGATTAATTCGATGCAACGCGAAGAACCTTACCTGGGTTTGACATGCACAGGACGCGTCTAGAGATAGGCGTTCCCTTGTGGCCTGTGTGCAGGTGGTGCATGGCTGTCGTCAGCTCGTGTCGTGAGATGTTGGGTTAAGTCCCGCAACGAGCGCAACCCTTGTCTCATGTTGCCAGCGGGTAATGCCGGGGACTCGTGAGAGACTGCCGGGGTCAACTCGGAGGAAGGTGGGGATGACGTCAAGTCATCATGCCCCTTATGTCCAGGGCTTCACACATGCTACAATGGCCGGTACAAAGGGCTGCGATGCCGCAAGGTTAAGCGAATCCTT-TTAAAGCCGGTCTCAGTTCGGATCGGGGTCTGCAACTCGACCCCGTGAAGTCGGAGTCGCTAGTAATCGCAGATCAGCAACGCTGCGGTGAATACGTTCCCGGGCCTTGTACACACCGCCCGTCACGTCATGAAAGTCGGTAACACCCGAAGCCAGTGGCCTAACCCTT---TGGGAGGGAGCTGTCGAAGGTGGGATCGGCGATTGGGACGAAGTCGTAACAAGGTAGCCGTACCGGAAGGTGCGGCTGGATCACCTCCTTT-------->M._bovis_BCG Strain PasteurT-------TTTGTTTGGAGAGTTTGATCCTGGCTCAGGACGAACGCTGGCGGCGTGCTTAACACATGCAAGTCGAACGGAAAGGTCTCT------------T--C---------------GGAGATACTCGAGTGGCGAACGGGTGAGTAACACGTGGGTGATCTGCCCTGCACTTC-GGGATAAGCCTGGGAAACTGGGTCTAATACCGGATAGGACCA-C-GGGATGCATGTCT-T-GTGGTGGAAAGCGCTT--T----A----GCGGTGTGGGATGAGCCCGCGGCCTATCAGCTTGTTGGTGGGGTGACGGCCTACCAAGGCGACGACGGGTAGCCGGCCTGAGAGGGTGTCCGGCCACACTGGGACTGAGATACGGCCCAGACTCCTACGGGAGGCAGCAGTGGGGAATATTGCACAATGGGCGCAAGCCTGATGCAGCGACGCCGCGTGGGGGATGACGGCCTTCGGGTTGTAAACCTCTTTCACCATCGACGAAGGTCCGG-GTTC--TCTC-GGATTGACGGTAGGTGGAGAAGAAGCACCGGCCAACTACGTGCCAGCAGCCGCGGTAATACGTAGGGTGCGAGCGTTGTCCGGAATTACTGGGCGTAAAGAGCTCGTAGGTGGTTTGTCGCGTTGTTCGTGAAATCTC-ACGGCTTAACTGTGAGCGTGCGGGCGATACGGGCAGACTAGAGTACTGCAGGGGAGACTGGAATTCCTGGTGTAGCGGTGGAATGCGCAGATATCAGGAGGAACACCGGTGGCGAAGGCGGGTCTCTGGGCAGTAACTGACGCTGAGGAGCGAAAGCGTGGGGAGCGAACAGGATTAGATACCCTGGTAGTCCACGCCGTAAACGGTGGGTACTAGGTGTGGGTTTCCTTCCTT--GGGATCCGTGCCGTAGCTAACGCATTAAGTACCCCGCCTGGGGAGTACGGCCGCAAGGCTAAAACTCAAAGGAATTGACGGGGGCCCGCACAAGCGGCGGAGCATGTGGATTAATTCGATGCAACGCGAAGAACCTTACCTGGGTTTGACATGCACAGGACGCGTCTAGAGATAGGCGTTCCCTTGTGGCCTGTGTGCAGGTGGTGCATGGCTGTCGTCAGCTCGTGTCGTGAGATGTTGGGTTAAGTCCCGCAACGAGCGCAACCCTTGTCTCATGTTGCCAGCACGTAATGGTGGGGACTCGTGAGAGACTGCCGGGGTCAACTCGGAGGAAGGTGGGGATGACGTCAAGTCATCATGCCCCTTATGTCCAGGGCTTCACACATGCTACAATGGCCGGTACAAAGGGCTGCGATGCCGCGAGGTTAAGCGAATCCTT--AAAAGCCGGTCTCAGTTCGGATCGGGGTCTGCAACTCGACCCCGTGAAGTCGGAGTCGCTAGTAATCGCAGATCAGCAACGCTGCGGTGAATACGTTCCCGGGCCTTGTACACACCGCCCGTCACGTCATGAAAGTCGGTAACACCCGAAGCCAGTGGCCTAACCCT----CGGGAGGGAGCTGTCGAAGGTGGGATCGGCGATTGGGACGAAGTCGTAACAAGGTAGCCGTACCGGAAGGTGCGGCTGGATCACCTCCTTTCT------>M._branderi---------TTGTTTGGAGAGTTTGATCCTGGCTCAGGACGAACGCTGGCGGCGTGCTTAACACATGCAAGTCGAACGGAAAGGTTCTT------------T--C---------------GGGAACACTCGAGTGGCGAACGGGTGAGTAACACGTGGGTGATCTGCCCTGCACTTC-GGGATAAGCTTGGGAAACTGGGTCTAATACCGGATAGGACCA-C-ATGCTGCATGGTG-T-GTGGTGGAAAG--CTTTTT----T----GCGGTGTGGGATGGGCCCGCGGCCTATCAGCTTGTTGGTGGGGTGATGGCCTACCAAGGCGACGACGGGTAGCCGGCCTGAGAGGGTGTCCGGCCACACTGGGACTGAGATACGGCCCAGACTCCTACGGGAGGCAGCAGTGGGGAATATTGCACAATGGGCGCAAGCCTGATGCAGCGACGCCGCGTGGGGGATGACGGCCTTCGGGTTGTAAACCTCTTTCACCATCGACGAAGCTTCAGCGTGT--TGTT-GGGGTGACGGTAGGTGGAGAAGAAGCACCGGCCAACTACGTGCCAGCAGCCGCGGTAATACGTAGGGTGCGAGCGTTGTCCGGAATTACTGGGCGTAAAGAGCTCGTAGGTGGTTTGTCGCGTTGTTCGTGAAATTCC-CTGGCTTAACTGGGGGCGTGCGGGCGATACGGGCAGACTGGAGTACTGCAGGGGAGACTGGAATTCCTGGTGTAGCGGTGGAATGCGCAGATATCAGGAGGAACACCGGTGGCGAAGGCGGGTCTCTGGGCAGTAACTGACGCTGAGGAGCGAAAGCGTGGGGAGCGAACAGGATTAGATACCCTGGTAGTCCACGCCGTAAACGGTGGGTACTAGGTGTGGGTTTCCTTCCTT--GGGATCCGTGCCGTAGCTAACGCATTAAGTACCCCGCCTGGGGAGTACGGCCGCAAGGCTAAAACTCAAAGGAATTGACGGGGGCCCGCACAAGCGGCGGAGCATGTGGATTAATTCGATGCAACGCGAAGAACCTTACCTGGGTTTGACATGCACAGGACGGCATCAGAGATGGTGCTTCCCTTGTGGTCTGTGTGCAGGTGGTGCATGGCTGTCGTCAGCTCGTGTCGTGAGATGTTGGGTTAAGTCCCGCAACGAGCGCAACCCTTGTCTCATGTTGCCAGCGCGTGATGGCGGGGACTCGTGAGAGACTGCCGGGGTCAACTCGGAGGAAGGTGGGGATGACGTCAAGTCATCATGCCCCTTATGTCCAGGGCTTCACACATGCTACAATGGCCGGTACAAAGGGCTGCGATGCCGTGAGGTTTAGCGAATCCTT-GTAAAGCCGGTCTCAGTTCGGATCGGGGTCTGCAACTCGACCCCGTGAAGTCGGAGTCGCTAGTAATCGCAGATCAGCAATGCTGCGGTGAATACGTTCCCGGGCCTTGTACACACCGCCCGTCACGTCATGAAAGTCGGTAACACCCGAAGCCCATGGCCTAACCCGC--AAGGGAGGGAGTGGTCGAAGGTGGGATCGGCGATTGGGACGAAGTCGTAACAAGGTAGCCGTACCGGAAGGTGCGGCTGGATCACCTCCTTTCTAAGGAG>M._canettii---------TTGGTTGGAGAGTTTGATCCTGGCTCAGGACGAACGCTGGCGGCGTGCTTAACACATGCAAGTCGAACGGAAAGGTCTCT------------T--C---------------GGAGATACTCGAGTGGCGAACGGGTGAGTAACACGTGGGTGATCTGCCCTGCACTTC-GGGATAAGCCTGGGAAACTGGGTCTAATACCGGATAGGACCA-C-GGGATGCATGTCT-T-GTGGTGGAAAGCGCTT--T----A----GCGGTGTGGGATGAGCCCGCGGCCTATCAGCTTGTTGGTGGGGTGACGGCCTACCAAGGCGACGACGGGTAGCCGGCCTGAGAGGGTGTCCGGCCACACTGGGACTGAGATACGGCCCAGACTCCTACGGGAGGCAGCAGTGGGGAATATTGCACAATGGGCGCAAGCCTGATGCAGCGACGCCGCGTGGGGGATGACGGCCTTCGGGTTGTAAACCTCTTTCACCATCGACGAAGGTCCGG-GTTC--TCTC-GGATTGACGGTAGGTGGAGAAGAAGCACCGGCCAACTACGTGCCAGCAGCCGCGGTAATACGTAGGGTGCGAGCGTTGTCCGGAATTACTGGGCGTAAAGAGCTCGTAGGTGGTTTGTCGCGTTGTTCGTGAAATCTC-ACGGCTTAACTGTGAGCGTGCGGGCGATACGGGCAGACTAGAGTACTGCAGGGGAGACTGGAATTCCTGGTGTAGCGGTGGAATGCGCAGATATCAGGAGGAACACCGGTGGCGAAGGCGGGTCTCTGGGCAGTAACTGACGCTGAGGAGCGAAAGCGTGGGGAGCGAACAGGATTAGATACCCTGGTAGTCCACGCCGTAAACGGTGGGTACTAGGTGTGGGTTTCCTTCCTT--GGGATCCGTGCCGTAGCTAACGCATTAAGTACCCCGCCTGGGGAGTACGGCCGCAAGGCTAAAACTCAAAGGAATTGACGGGGGCCCGCACAAGCGGCGGAGCATGTGGATTAATTCGATGCAACGCGAAGAACCTTACCTGGGTTTGACATGCACAGGACGCGTCTAGAGATAGGCGTTCCCTTGTGGCCTGTGTGCAGGTGGTGCATGGCTGTCGTCAGCTCGTGTCGTGAGATGTTGGGTTAAGTCCCGCAACGAGCGCAACCCTTGTCTCATGTTGCCAGCACGTAATGGTGGGGACTCGTGAGAGACTGCCGGGGTCAACTCGGAGGAAGGTGGGGATGACGTCAAGTCATCATGCCCCTTATGTCCAGGGCTTCACACATGCTACAATGGCCGGTACAAAGGGCTGCGATGCCGCGAGGTTAAGCGAATCCTT--AAAAGCCGGTCTCAGTTCGGATCGGGGTCTGCAACTCGACCCCGTGAAGTCGGAGTCGCTAGTAATCGCAGATCAGCAACGCTGCGGTGAATACGTTCCCGGGCCTTGTACACACCGCCCGTCACGTCATGAAAGTCGGTAACACCCGAAGCCAGTGGCCTAACCCT----CGGGAGGGAGCTGTCGAAGGTGGGATCGGCGATTGGGACGAAGTCGTAACAAGGTAGCCGTACCGGAAGGTGCGGCTGGATCACCTCCTTTCTAAGGAG>M._colombiense----------TGTTTGGAGAGTTTGATCCTGGCTCAGGACGAACGCTGGCGGCGTGCTTAACACATGCAAGTCGAACGGAAAGGCCTCT------------T--C---------------GGAGGTACTCGAGTGGCGAACGGGTGAGTAACACGTGGGCAATCTGCCCTGCACTTC-GGGATAAGCCTGGGAAACTGGGTCTAATACCGGATAGGACCT-T-TAGACGCATGTCT-T-TTGGTGGAAAG--CTT--T----T----GCGGTGTGGGATGGGCCCGCGGCCTATCAGCTTGTTGGTGGGGTGATGGCCTACCAAGGCGACGACGGGTAGCCGGCCTGAGAGGGTGTCCGGCCACACTGGGACTGAGATACGGCCCAGACTCCTACGGGAGGCAGCAGTGGGGAATATTGCACAATGGGCGCAAGCCTGATGCAGCGACGCCGCGTGGGGGATGACGGCCTTCGGGTTGTAAACCTCTTTCACCATCGACGAAGGTCCGG-GTTT--TCTC-GGATTGACGGTAGGTGGAGAAGAAGCACCGGCCAACTACGTGCCAGCAGCCGCGGTAATACGTAGGGTGCGAGCGTTGTCCGGAATTACTGGGCGTAAAGAGCTCGTAGGTGGTTTGTCGCGTTGTTCGTGAAATCTC-ACGGCTTAACTGTGAGCGTGCGGGCGATACGGGCAGACTAGAGTACTGCAGGGGAGACTGGAATTCCTGGTGTAGCGGTGGAATGCGCAGATATCAGGAGGAACACCGGTGGCGAAGGCGGGTCTCTGGGCAGTAACTGACGCTGAGGAGCGAAAGCGTGGGGAGCGAACAGGATTAGATACCCTGGTAGTCCACGCCGTAAACGGTGGGTACTAGGTGTGGGTTTCCTTCCTT--GGGATCCGTGCCGTAGCTAACGCATTAAGTACCCCGCCTGGGGAGTACGGCCGCAAGGCTAAAACTCAAAGGAATTGACGGGGGCCCGCACAAGCGGCGGAGCATGTGGATTAATTCGATGCAACGCGAAGAACCTTACCTGGGTTTGACATGCACAGGACGCGTCTAGAGATAGGCGTTCCCTTGTGGCCTGTGTGCAGGTGGTGCATGGCTGTCGTCAGCTCGTGTCGTGAGATGTTGGGTTAAGTCCCGCAACGAGCGCAACCCTTGTCTCATGTTGCCAGCGGGTAATGCCGGGGACTCGTGAGAGACTGCCGGGGTCAACTCGGAGGAAGGTGGGGATGACGTCAAGTCATCATGCCCCTTATGTCCAGGGCTTCACACATGCTACAATGGCCGGTACAAAGGGCTGCGATGCCGTAAGGTTAAGCGAATCCTT-TTAAAGCCGGTCTCAGTTCGGATTGGGGTCTGCAACTCGACCCCATGAAGTCGGAGTCGCTAGTAATCGCAGATCAGCAACGCTGCGGTGAATACGTTCCCGGGCCTTGTACACACCGCCCGTCACGTCATGAAAGTCGGTAACACCCGAAGCCAGTGGCCTAACCTT----TTGGAGGGAGCTGTCGAAGGTGGGATCGGCGATTGGGACGAAGTCGTAACAAGGTAGCCGTACCGGAAGGTGCGGCTGGATCACCTCCTTT-------->M._conspicuum---------TTGTTTGGAGAGTTTGATCCTGGCTCAGGACGAACGCTGGCGGCGTGCTTAACACATGCAAGTCGAACGGAAAGGCCCCT------------T--C---------------GGGGGTACTCGAGTGGCGAACGGGTGAGTAACACGTGGGTGATCTGCCCTGCACTTC-GGGATAAGCCTGGGAAACTGGGTCTAATACCGGATAGGACCA-C-TTGGCGCATGCCT-T-GTGGTGGAAAG--CTT--T----T----GCGGTGTGGGATGGGCCCGCGGCCTATCAGCTTGTTGGTGGGGTCACGGCCTACCAAGGCTCCGACGGGTAGCCGGCCTGAGAGGGTGTCCGGCCACACTGGGACTGAGATACGGCCCAGACTCCTACGGGAGGCAGCAGTGGGGAATATTGCACAATGGGCGCAAGCCTGATGCAGCGACGCCGCGTGGGGGATGACGGCCTTCGGGTTGTAAACCTCTTTCACCATCGACGAAGGTCCGG-GTTT--TCTC-GGATTGACGGTAGGTGGAGAAGAAGCACCGGCCAACTACGTGCCAGCAGCCGCGGTAATACGTAGGGTGCGAGCGTTGTCCGGAATTACTGGGCGTAAAGAGCTCGTAGGTGGTTTGTCGCGTTGTTCGTGAAAACTC-ACGGCTTAACTGTGAGCGTGCGGGCGATACGGGCAGACTGGAGTACTGCAGGGGAGACTGGAATTCCTGGTGTAGCGGTGGAATGCGCAGATATCAGGAGGAACACCGGTGGCGAAGGCGGGTCTCTGGGCAGTAACTGACGCTGAGGAGCGAAAGCGTGGGGAGCGAACAGGATTAGATACCCTGGTAGTCCACGCCGTAAACGGTGGGTACTAGGTGTGGGTTTCCTTCCTT--GGGATCCGTGCCGTAGCTAACGCATTAAGTACCCCGCCTGGGGAGTACGGCCGCAAGGCTAAAACTCAAAGGAATTGACGGGGGCCCGCACAAGCGGCGGAGCATGTGGATTAATTCGATGCAACGCGAAGAACCTTACCTGGGTTTGACATGCACAGGACGCTGGTAGAGATATCAGTTCCCTTGTGGCCTGTGTGCAGGTGGTGCATGGCTGTCGTCAGCTCGTGTCGTGAGATGTTGGGTTAAGTCCCGCAACGAGCGCAACCCTTGTCTCATGTTGCCAGCGGGTAATGCCGGGGACTCGTGAGAGACTGCCGGGGTCAACTCGGAGGAAGGTGGGGATGACGTCAAGTCATCATGCCCCTTATGTCCAGGGCTTCACACATGCTACAATGGCCGGTACAAAGGGCTGCGATGCCGCAAGGTTAAGCGAATCCTT--TAAAGCCGGTCTCAGTTCGGATCGGGGTCTGCAACTCGACCCCGTGAAGTCGGAGTCGCTAGTAATCGCAGATCAGCAACGCTGCGGTGAATACGTTCCCGGGCCTTGTACACACCGCCCGTCACGTCATGAAAGTCGGTAACACCCGAAGCCAGTGGCCTAACCCTT---TGGGAGGGAGCTGTCGAAGGTGGGATCGGCGATTGGGACGAAGTCGTAACAAGGTAGCCGTACCGGAAGGTGCGGCTGGATCACCTCCTTTCTAAGGAG>M._cookii---------TTGTTTGGAGAGTTTGATCCTGGCTCAGGACGAACGCTGGCGGCGTGCTTAACACATGCAAGTCGAACGGAAAGGCCCTT------------T--C---------------GGGGGTACTCGAGTGGCGAACGGGTGAGTAACACGTGGGTGATCTGCCCTGCACTTT-GGGATAAGCCTGGGAAACTGGGTCTAATACCGGATAGGACTA-C-GGACTGCATGGTC-T-GTGGTGGAAAG--CTT-GT----T----GCGGTGTGGGATGGGCCCGCGGCCTATCAGCTTGTTGGTGGGGTGATGGCCTACCAAGGCGACGACGGGTAGCCGGCCTGAGAGGGTGTCCGGCCACACTGGGACTGAGATACGGCCCAGACTCCTACGGGAGGCAGCAGTGGGGAATATTGCACAATGGGCGCAAGCCTGATGCAGCGACGCCGCGTGGGGGATGACGGCCTTCGGGTTGTAAACCTCTTTCACCATCGGCGAAGCCGGGA-GCTTTTGTTT-CTGGTGACGGTAGGTGGAGAAGAAGCACCGGCCAACTACGTGCCAGCAGCCGCGGTAATACGTAGGGTGCGAGCGTTGTCCGGAATTACTGGGCGTAAAGAGCTCGTAGGTGGTTTGTCGCGTTGTCCGTGAAATTCC-CTGGCTTAACTGGGGGCGTGCGGGCGATACGGGCAGACTGGAGTACTGCAGGGGAGACTGGAATTCCTGGTGTAGCGGTGGAATGCGCAGATATCAGGAGGAACACCGGTGGCGAAGGCGGGTCTCTGGGCAGTAACTGACGCTGAGGAGCGAAAGCGTGGGGAGCGAACAGGATTAGATACCCTGGTAGTCCACGCCGTAAACGGTGGGTACTAGGTGTGGGTTTCCTTCCTT--GGGATCCGTGCCGTAGCTAACGCATTAAGTACCCCGCCTGGGGAGTACGGCCGCAAGGCTAAAACTCAAAGGAATTGACGGGGGCCCGCACAAGCGGCGGAGCATGTGGATTAATTCGATGCAACGCGAAGAACCTTACCTGGGTTTGACATGCACAGGACGCCGGCAGAGATGTCGGTTCCCTTGTGGCCTGTGTGCAGGTGGTGCATGGCTGTCGTCAGCTCGTGTCGTGAGATGTTGGGTTAAGTCCCGCAACGAGCGCAACCCTTGTCTCATGTTGCCAGCGCGTTATGGCGGGGACTCGTGAGAGACTGCCGGGGTCAACTCGGAGGAAGGTGGGGATGACGTCAAGTCATCATGCCCCTTATGTCCAGGGCTTCACACATGCTACAATGGCCGGTACAAAGGGCTGCGATGCCGTGAGGCTTAGCGAATCCTT--GAAAGCCGGTCTCAGTTCGGATCGGGGTCTGCAACTCGACCCCGTGAAGTCGGAGTCGCTAGTAATCGCAGATCAGCAACGCTGCGGTGAATACGTTCCCGGGCCTTGTACACACCGCCCGTCACGTCATGAAAGTCGGTAACACCCGAAGCCAGTGGCCTAACCCTT---GTGGGGGGAGCTGTCGAAGGTGGGATCGGCGATTGGGACGAAGTCGTAACAAGGTAGCCGTACCGGAAGGTGCGGCTGGATCACCTCCTTTCTAAGGAG>M._dioxanotrophicus----------TGTTTGGAGAGTTTGATCCTGGCTCAGGACGAACGCTGGCGGCGTGCTTAACACATGCAAGTCGAACGGAAAGGCCC-T------------T--C----------------GGGGTGCTCGAGTGGCGAACGGGTGAGTAACACGTGGGTGATCTGCCCTGCACTTT-GGGATAAGCCTGGGAAACTGGGTCTAATACCGGATAGGACCA-T-GGCCTTCATGGGT-T-GTGGTGGAAAG--CTT--T----T----GCGGTGTGGGATGGGCCCGCGGCCTATCAGCTTGTTGGTGGGGTAATGGCCTACCAAGGCGACGACGGGTAGCCGGCCTGAGAGGGTGACCGGCCACACTGGGACTGAGATACGGCCCAGACTCCTACGGGAGGCAGCAGTGGGGAATATTGCACAATGGGCGCAAGCCTGATGCAGCGACGCCGCGTGGGGGATGACGGCCTTCGGGTTGTAAACCTCTTTCAGCACAGACGAAGCGT----------------GAGTGACGGTATGTGCAGAAGAAGCACCGGCCAACTACGTGCCAGCAGCCGCGGTAATACGTAGGGTGCGAGCGTTGTCCGGAATTACTGGGCGTAAAGAGCTCGTAGGTGGTTTGTCGCGTTGTTCGTGAAAACTC-ACAGCTTAACTGTGGGCGTGCGGGCGATACGGGCAGACTGGAGTACTGCAGGGGAGACTGGAATTCCTGGTGTAGCGGTGGAATGCGCAGATATCAGGAGGAACACCGGTGGCGAAGGCGGGTCTCTGGGCAGTAACTGACGCTGAGGAGCGAAAGCGTGGGGAGCGAACAGGATTAGATACCCTGGTAGTCCACGCCGTAAACGGTGGGTACTAGGTGTGGGTTTCCTTCCTT--GGGATCCGTGCCGTAGCTAACGCATTAAGTACCCCGCCTGGGGAGTACGGCCGCAAGGCTAAAACTCAAAGGAATTGACGGGGGCCCGCACAAGCGGCGGAGCATGTGGATTAATTCGATGCAACGCGAAGAACCTTACCTGGGTTTGACATGCACAGGACGCTGGTAGAGATATCAGTTCCCTTGTGGCCTGTGTGCAGGTGGTGCATGGCTGTCGTCAGCTCGTGTCGTGAGATGTTGGGTTAAGTCCCGCAACGAGCGCAACCCTTGTCCTATGTTGCCAGCGGGTTATGCCGGGGACTCGTAGGAGACTGCCGGGGTCAACTCGGAGGAAGGTGGGGATGACGTCAAGTCATCATGCCCCTTATGTCCAGGGCTTCACACATGCTACAATGGCCGGTACAAAGGGCTGCGATACCGTGAGGTGGAGCGAATCCTT-TCAAAGCCGGTCTCAGTTCGGATCGGGGTCTGCAACTCGACCCCGTGAAGTCGGAGTCGCTAGTAATCGCAGATCAGCAACGCTGCGGTGAATACGTTCCCGGGCCTTGTACACACCGCCCGTCACGTCATGAAAGTCGGTAACACCCGAAGCCGGTGGCCTAACCCCT-TGTGGGAGGGAGCCGTCGAAGGTGGGATCGGCGATTGGGACGAAGTCGTAACAAGGTAGCCGTACCGGAAGGTGCGGCTGGATCACCTCCTTT-------->M._europaeum----------TGTTTGGAGAGTTTGATCCTGGCTCAGGACGAACGCTGGCGGCGTGCTTAACACATGCAAGTCGAACGGAAAGGCCCCT------------T--C---------------GGGGGTACTCGAGTGGCGAACGGGTGAGTAACACGTGGGCAATCTGCCCTGCACTTC-GGGATAAGCCTGGGAAACTGGGTCTAATACCGGATAGGACCA-C-TTGGCGCATGCCT-T-GTGGTGGAAAG--CTT--T----T----GCGGTGTGGGATGGGCCCGCGGCCTATCAGCTTGTTGGTGGGGTGATGGCCTACCAAGGCGACGACGGGTAGCCGGCCTGAGAGGGTGTCCGGCCACACTGGGACTGAGATACGGCCCAGACTCCTACGGGAGGCAGCAGTGGGGAATATTGCACAATGGGCGCAAGCCTGATGCAGCGACGCCGCGTGGGGGATGACGGCCTTCGGGTTGTAAACCTCTTTCAGCAGGGACGAAGCGA----------------AAGTGACGGTACCTGCAGAAGAAGCACCGGCCAACTACGTGCCAGCAGCCGCGGTAATACGTAGGGTGCGAGCGTTGTCCGGAATTACTGGGCGTAAAGAGCTCGTAGGTGGTTTGTCGCGTTGTTCGTGAAAACCG-GGGGCTTAACCCTCGGCGTGCGGGCGATACGGGCAGACTAGAGTACTGCAGGGGAGACTGGAATTCCTGGTGTAGCGGTGGAATGCGCAGATATCAGGAGGAACACCGGTGGCGAAGGCGGGTCTCTGGGCAGTAACTGACGCTGAGGAGCGAAAGCGTGGGGAGCGAACAGGATTAGATACCCTGGTAGTCCACGCCGTAAACGGTGGGTACTAGGTGTGGGTTTCCTTCCTT--GGGATCCGTGCCGTAGCTAACGCATTAAGTACCCCGCCTGGGGAGTACGGCCGCAAGGCTAAAACTCAAAGGAATTGACGGGGGCCCGCACAAGCGGCGGAGCATGTGGATTAATTCGATGCAACGCGAAGAACCTTACCTGGGTTTGACATGCACAGGACGCGTCTAGAGATAGGCGTTCCCTTGTGGCCTGTGTGCAGGTGGTGCATGGCTGTCGTCAGCTCGTGTCGTGAGATGTTGGGTTAAGTCCCGCAACGAGCGCAACCCTTGTCTCATGTTGCCAGCGGGTAATGCCGGGGACTCGTGAGAGACTGCCGGGGTCAACTCGGAGGAAGGTGGGGATGACGTCAAGTCATCATGCCCCTTATGTCCAGGGCTTCACACATGCTACAATGGCCGGTACAAAGGGCTGCGATGCCGCAAGGTTAAGCGAATCCTT-TTAAAGCCGGTCTCAGTTCGGATCGGGGTCTGCAACTCGACCCCGTGAAGTCGGAGTCGCTAGTAATCGCAGATCAGCAACGCTGCGGTGAATACGTTCCCGGGCCTTGTACACACCGCCCGTCACGTCATGAAAGTCGGTAACACCCGAAGCCAGTGGCCTAACCCT----TGGGAGGGAGCTGTCGAAGGTGGGATCGGCGATTGGGACGAAGTCGTAACAAGGTAGCCGTACCGGAAGGTGCGGCTGGATCACCTCCTTT-------->M._florentinum---------TTGTTTGGAGAGTTTGATCCTGGCTCAGGACGAACGCTGGCGGCGTGCTTAACACATGCAAGTCGAACGGAAAGGCCCCT------------T--C---------------GGGGGTACTCGAGTGGCGAACGGGTGAGTAACACGTGGGTAATCTGCCCTGCACTTC-GGGATAAGCCTGGGAAACTGGGTCTAATACCGGATATGACCA-C-GAGACGCATGTCT-T-GTGGTGGAAAG--CTT--T----T----GCGGTGTGGGATGGGCCCGCGGCCTATCAGCTTGTTGGTGGGGTGATGGCCTACCAAGGCGACGACGGGTAGCCGGCCTGAGAGGGTGTCCGGCCACACTGGGACTGAGATACGGCCCAGACTCCTACGGGAGGCAGCAGTGGGGAATATTGCACAATGGGCGCAAGCCTGATGCAGCGACGCCGCGTGGGGGATGACGGCCTTCGGGTTGTAAACCTCTTTCAGCAGGGACGAAGCGC----------------AAGTGACGGTACCTGCAGAAGAAGCACCGGCCAACTACGTGCCAGCAGCCGCGGTAATACGTAGGGTGCGAGCGTTGTCCGGAATTACTGGGCGTAAAGAGCTCGTAGGTGGTTTGTCGCGTTGTTCGTGAAAACCG-GGGGCTTAACCCTCGGCGTGCGGGCGATACGGGCAGACTGGAGTACTGCAGGGGAGACTGGAATTCCTGGTGTAGCGGTGGAATGCGCAGATATCAGGAGGAACACCGGTGGCGAAGGCGGGTCTCTGGGCAGTAACTGACGCTGAGGAGCGAAAGCGTGGGGAGCGAACAGGATTAGATACCCTGGTAGTCCACGCCGTAAACGGTGGGTACTAGGTGTGGGTTTCCTTCCTT--GGAATCCGTGCCGTAGCTAACGCATTAAGTACCCCGCCTGGGGAGTACGGCCGCAAGGCTAAAACTCAAAGGAATTGACGGGGGCCCGCACAAGCGGCGGAGCATGTGGATTAATTCGATGCAACGCGAAGAACCTTACCTGGGTTTGACATGCACAGGACGCCGGCAGAGATGTCGGTTCCCTTGTGGCCTGTGTGCAGGTGGTGCATGGCTGTCGTCAGCTCGTGTCGTGAGATGTTGGGTTAAGTCCCGCAACGAGCGCAACCCTTGTCTCATGTTGCCAGCACGTAATGGTGGGGACTCGTGAGAGACTGCCGGGGTCAACTCGGAGGAAGGTGGGGATGACGTCAAGTCATCATGCCCCTTATGTCCAGGGCTTCACACATGCTACAATGGCCGGTACAAAGGGCTGCGATGCCGTAAGGTTAAGCGAATCCTT-TTAAAGCCGGTCTCAGTTCGGATCGGGGTCTGCAACTCGACCCCGTGAAGTCGGAGTCGCTAGTAATCGCAGATCAGCAACGCTGCGGTGAATACGTTCCCGGGCCTTGTACACACCGCCCGTCACGTCATGAAAGTCGGTAACACCCGAAGCCAGTGGCCTAACCTT----TTGGAGGGAGCTGTCGAAGGTGGGATCGGCGATTGGGACGAAGTCGTAACAAGGTAGCCGTACCGGAAGGTGCGGCTGGATCACCTCCTTTCTAA---->M._fragae----------TGTTTGGAGAGTTTGATCCTGGCTCAGGACGAACGCTGGCGGCGTGCTTAACACATGCAAGTCGAACGGAAAGGCTCTC------------TT-CG--------------GAGGGTGCTCGAGTGGCGAACGGGTGAGTAACACGTGGGTGATCTGCCCTGCACTTC-GGGATAAGCTTGGGAAACTGGGTCTAATACCGGATAGGACCA-T-GGGATGCATGTCT-T-GTGGTGGAAAG--CTT--T---TT----GCGGTGTGGGATGGGCCCGCGGCCTATCAGCTTGTTGGTGGGGTGATGGCCTACCAAGGCGACGACGGGTAGCCGGCCTGAGAGGGTGTCCGGCCACACTGGGACTGAGATACGGCCCAGACTCCTACGGGAGGCAGCAGTGGGGAATATTGCACAATGGGCGCAAGCCTGATGCAGCGACGCCGCGTGGGGGATGACGGCCTTCGGGTTGTAAACCTCTTTCACCATCGACGAAGCTTTGG-GTAA----CC-GGGGTGACGGTAGGTGGAGAAGAAGCACCGGCCAACTACGTGCCAGCAGCCGCGGTAATACGTAGGGTGCGAGCGTTGTCCGGAATTACTGGGCGTAAAGAGCTCGTAGGTGGTTTGTCGCGTTGTTCGTGAAATTCC-CTGGCTTAACTGGGGGCGTGCGGGCGATACGGGCAGACTGGAGTACTGCAGGGGAGACTGGAATTCCTGGTGTAGCGGTGGAATGCGCAGATATCAGGAGGAACACCGGTGGCGAAGGCGGGTCTCTGGGCAGTAACTGACGCTGAGGAGCGAAAGCGTGGGGAGCGAACAGGATTAGATACCCTGGTAGTCCACGCCGTAAACGGTGGGTACTAGGTGTGGGTTTCCTTCCTT--GGGATCCGTGCCGTAGCTAACGCATTAAGTACCCCGCCTGGGGAGTACGGCCGCAAGGCTAAAACTCAAAGGAATTGACGGGGGCCCGCACAAGCGGCGGAGCATGTGGATTAATTCGATGCAACGCGAAGAACCTTACCTGGGTTTGACATGCACAGGACGACCGTAGAGATGCGGTTTCCCTTGTGGCCTGTGTGCAGGTGGTGCATGGCTGTCGTCAGCTCGTGTCGTGAGATGTTGGGTTAAGTCCCGCAACGAGCGCAACCCTTGTCTCATGTTGCCAGCACGTGATGGTGGGGACTCGTGAGAGACTGCCGGGGTCAACTCGGAGGAAGGTGGGGATGACGTCAAGTCATCATGCCCCTTATGTCCAGGGCTTCACACATGCTACAATGGCCGGTACAAAGGGCTGCGATGCCGTGAGGTTAAGCGAATCCTTGGTAAAGCCGGTCTCAGTTCGGATCGGGGTCTGCAACTCGACCCCGTGAAGTCGGAGTCGCTAGTAATCGCAGATCAGCAACGCTGCGGTGAATACGTTCCCGGGCCTTGTACACACCGCCCGTCACGTCATGAAAGTCGGTAACACCCGAAGCCCATGGCCCAACCCTT-TTTGGGAGGGAGTGGTCGAAGGTGGGATCGGCGATTGGGACGAAGTCGTAACAAGGTAGCCGTACCGGAAGGTGCGGCTGGATCACCTCCTTT-------->M._gallinarum---------TTGTTTGGAGAGTTTGATTCTGGCTCAGGACGAACGCTGGCGGCGTGCTTAACACATGCAAGTCGAACGGAAAGGCCCTT------------T--C---------------GGGGGTACTCGAGTGGCGAACGGGTGAGTAACACGTGGGTGATCTGCCCTGCACTTT-GGGATAAGCCTGGGAAACTGGGTCTAATACCGGATAGGACTA-C-GCGATGCATGTCG-T-GTGGTGGAAAG--CTT--T----T----GCGGTGTGGGATGGGCCCGCGGCCTATCAGCTTGTTGGTGGGGTGATGGCCTACCAAGGCGACGACGGGTAGCCGGCCTGAGAGGGTGACCGGCCACACTGGGACTGAGATACGGCCCAGACTCCTACGGGAGGCAGCAGTGGGGAATATTGCACAATGGGCGCAAGCCTGATGCAGCGACGCCGCGTGAGGGATGACGGCCTTCGGGTTGTAAACCTCTTTCAGCTCCGACGAAGCGC----------------AAGTGACGGTAGG-GTAGAAGAAGGACCGGCCAACTACGTGCCAGCAGCCGCGGTAATACGTAGGGTCCGAGCGTTGTCCGGAATTACTGGGCGTAAAGAGCTCGTAGGTGGTTTGTCGCGTTGTTCGTGAAAACTC-ACAGCTCAACTGTGGGCGTGCGGGCGATACGGGCAGACTTGAGTACTGCAGGGGAGACTGGAATTCCTGGTGTAGCGGTGGAATGCGCAGATATCAGGAGGAACACCGGTGGCGAAGGCGGGTCTCTGGGCAGTAACTGACGCTGAGGAGCGAAAGCGTGGGGAGCGAACAGGATTAGATACCCTGGTAGTCCACGCCGTAAACGGTGGGTACTAGGTGTGGGTTTCCTTCCTT--GGGATCCGTGCCGTAGCTAACGCATTAAGTACCCCGCCTGGGGAGTACGGCCGCAAGGCTAAAACTCAAAGAAATTGACGGGGGCCCGCACAAGCGGCGGAGCATGTGGATTAATTCGATGCAACGCGAAGAACCTTACCTGGGTTTGACATGCACAGGACGTGCCTAGAGATAGGTATTCCCTTGTGGCCTGTGTGCAGGTGGTGCATGGCTGTCGTCAGCTCGTGTCGTGAGATGTTGGGTTAAGTCCCGCAACGAGCGCAACCCTTGTCTCATGTTGCCAGCACGTTATGGTGGGGACTCGTGAGAGACTGCCGGGGTCAACTCGGAGGAAGGTGGGGATGACGTCAAGTCATCATGCCCCTTATGTCCAGGGCTTCACACATGCTACAATGGCCGGTACAAAGGGCTGCGATGCCGTGAGGTGGAGCGAATCCTT-TCAAAGCCGGTCTCAGTTCGGATCGGGGTCTGCAACTCGACCCCGTGAAGTCGGAGTCGCTAGTAATCGCAGATCAGCAACGCTGCGGTGAATACGTTCCCGGGCCTTGTACACACCGCCCGTCACGTCATGAAAGTCGGTAACACCCGAAGCCGGTGGCCTAACCCCT-TGTGGGAGGGAGCCGTCGAAGGTGGGATCGGCGATTGGGACGAAGTCGTAACAAGGTAGCCGTACCGGAAGGTGCGGCTGGATCACCTCCTTTCTAA---->M._genavense---------TTGTTTGGAGAGTTTGATCCTGGCTCAGGACGAACGCTGGCGGCGTGCTTAACACATGCAAGTCGAACGGAAAGGCCTCT------------T--C---------------GGAGGTACTCGAGTGGCGAACGGGTGAGTAACACGTGGGTAATCTGCCCTGCACTTC-GGGATAAGCCTGGGAAACTGGGTCTAATACCGGATATGACCA-C-GGAACGCATGTTT-T-GTGGTGGAAAG--CTT--T----T----GCGGTGTGGGATGGGCCCGCGGCCTATCAGCTTGTTGGTGGGGTGACGGCCTACCAAGGCGACGACGGGTAGCCGGCCTGAGAGGGTGTCCGGCCACACTGGGACTGAGATACGGCCCAGACTCCTACGGGAGGCAGCAGTGGGGAATATTGCACAATGGGCGCAAGCCTGATGCAGCGACGCCGCGTGGGGGATGACGGCCTTCGGGTTGTAAACCTCTTTCAGCAGGGACGAAGCGC----------------AAGTGACGGTACCTGCAGAAGAAGCACCGGCCAACTACGTGCCAGCAGCCGCGGTAATACGTAGGGTGCGAGCGTTGTCCGGAATTACTGGGCGTAAAGAGCTCGTAGGTGGTTTGTCGCGTTGTTCGTGAAAACCG-GGGGCTTAACCCTCGGCGTGCGGGCGATACGGGCAGACTGGAGTACTGCAGGGGAGACTGGAATTCCTGGTGTAGCGGTGGAATGCGCAGATATCAGGAGGAACACCGGTGGCGAAGGCGGGTCTCTGGGCAGTAACTGACGCTGAGGAGCGAAAGCGTGGGGAGCGAACAGGATTAGATACCCTGGTAGTCCACGCCGTAAACGGTGGGTACTAGGTGTGGGTTTCCTTCCTT--GGAATCCGTGCCGTAGCTAACGCATTAAGTACCCCGCCTGGGGAGTACGGCCGCAAGGCTAAAACTCAAAGGAATTGACGGGGGCCCGCACAAGCGGCGGAGCATGTGGATTAATTCGATGCGACGCGAAGAACCTTACCTGGGTTTGACATGCACAGGACGCCGGCAGAGATGTCGGTTCCCTTGTGGCCTGTGTGCAGGTGGTGCATGGCTGTCGTCAGCTCGTGTCGTGAGATGTTGGGTTAAGTCCCGCAACGAGCGCAACCCTTGTCTCATGTTGCCAGCGGGTAATGCCGGGGACTCGTGAGAGACTGCCGGGGTCAACTCGGAGGAAGGTGAGGATGACGTCAAGTCATCATGCCCCTTATGTCCAGGGCTTCACACATGCTACAATGGCCAGTACAAAGGGCTGCGATGCCGTAAGGTTAAGCGAATCCTT-TTAAAGCCGGTCTCAGTTCGGATCGGGGTCTGCAACTCGACCCCGTGAAGTCGGAGTCGCTAGTAATCGCAGATCAGCAACGCTGCGGTGAATACGTTCCCGGGCCTTGTACACACCGCCCGTCACGTCATGAAAGTCGGTAACACCCGAAGCCAGTGGCCTAACCTT----TTGGAGGGAGCTGTCGAAGGTGGGATCGGCGATTGGGACGAAGTCGTAACAAGGTAGCCGTACCGGAAGGTGCGGCTGGATCACCTCCTTTCTAA---->M._gordonae---------TTGTTTGGAGAGTTTGATTCTGGCTCAGGACGAACGCTGGCGGCGTGCTTAACACATGCAAGTCGAACGGTAAGGCCC-T------------T--C----------------GGGGTACACGAGTGGCGAACGGGTGAGTAACACGTGGGTAATCTGCCCTGCACATC-GGGATAAGCCTGGGAAACTGGGTCTAATACCGAATAGGACCA-C-AGAACACATGTCC-T-GTGGTGGAAAG--CTT--T----T----GCGGTGTGGGATGGGCCCGCGGCCTATCAGCTTGTTGGTGGGGTGATGGCCCACCAAGGCGACGACGGGTAGCCGGCCTGAGAGGGTGTCCGGCCACACTGGGACTGAGATACGGCCCAGACTCCTACGGGAGGCAGCAGTGGGGAATATTGCACAATGGGCGAAAGCCTGATGCAGCGACGCCGCGTGGGGGATGACGGCCTTCGGGTTGTAAACCTCTTTCACCATCGACGAAGGTCCGG-GTGT--TCTC-GGGCTGACGGTAGGTGGAGAAGAAGCACCGGCCAACTACGTGCCAGCAGCCGCGGTAATACGTAGGGTGCGAGCGTTGTCCGGAATTACTGGGCGTAAAGAGCTCGTAGGTGGTTTGTCGCGTTGTTCGTGAAATCTC-ACGGCTTAACTGTGAGCGTGCGGGCGATACGGGCAGACTTGAGTACTGCAGGGGAGACTGGAATTCCTGGTGTAGCGGTGGAATGCGCAGATATCAGGAGGAACACCGGTGGCGAAGGCGGGTCTCTGGGCAGTAACTGACGCTGAGGAGCGAAAGCGTGGGGAGCGAACAGGATTAGATACCCTGGTAGTCCACGCCGTAAACGGTGGGTACTAGGTGTGGGTTTCCTTCCTT--GGGATCCGTGCCGTAGCTAACGCATTAAGTACCCCGCCTGGGGAGTACGGCCGCAAGGCTAAAACTCAAAGAAATTGACGGGGGCCCGCACAAGCGGCGGAGCATGTGGATTAATTCGATGCAACGCGAAGAACCTTACCTGGGTTTGACATGCACAGGACGCCGGCAGAGATGTCGGTTCCCTTGTGGCCTGTGTGCAGGTGGTGCATGGCTGTCGTCAGCTCGTGTCGTGAGATGTTGGGTTAAGTCCCGCAACGAGCGCAACCCTTGTCTCATGTTGCCAGCGGGTAATGCCGGGGACTCGTGAGAGACTGCCGGGGTCAACTCGGAGGAAGGTGGGGATGACGTCAAGTCATCATGCCCCTTATGTCCAGGGCTTCACACATGCTACAATGGCCGGTACAAAGGGCTGCGATGCCGCGAGGTTAAGCGAATCCTT-TTAAAGCCGGTCTCAGTTCGGATCGGGGTCTGCAACTCGACCCCGTGAAGTCGGAGTCGCTAGTAATCGCAGATCAGCAACGCTGCGGTGAATACGTTCCCGGGCCTTGTACACACCGCCCGTCACGTCATGAAAGTCGGTAACACCCGAAGCCAGTGGCCTAACCCTT---TGGGAGGGAGCTGTCGAAGGTGGGATCGGCGATTGGGACGAAGTCGTAACAAGGTAGCCGTACCGGAAGGTGCGGCTGGATCACCTCCTTTCTAAGGAG>M._haemophilum---------TTGTTTGGAGAGTTTGATCCTGGCTCAGGACGAACGCTGGCGGCGTGCTTAACACATGCAAGTCGAACGGAAAGGTCTCT------------T--C---------------GGAGATACTCGAGTGGCGAACGGGTGAGTAACACGTGGGTAATCTGCCCTGCACTTC-GGGATAAGCCTGGGAAACTGGGTCTAATACCGGATAGGACCT-C-AAGGCGCATGCCTTT-GTGGTGGAAAG--CTT--T----T----GCGGTGTGGGATGGGCCCGCGGCCTATCAGCTTGTTGGTGGGGTGACGGCCTACCAAGGCGACGACGGGTAGCCGGCCTGAGAGGGTGTCCGGCCACACTGGGACTGAGATACGGCCCAGACTCCTACGGGAGGCAGCAGTGGGGAATATTGCACAATGGGCGCAAGCCTGATGCAGCGACGCCGCGTGGGGGATGACGGCCTTCGGGTTGTAAACCTCTTTCACCATCGACGAAGGTTCGG-GTTT--TCTC-GGATTGACGGTAGGTGGAGAAGAAGCACCGGCCAACTACGTGCCAGCAGCCGCGGTAATACGTAGGGTGCGAGCGTTGTCCGGAATTACTGGGCGTAAAGAGCTCGTAGGTGGTTTGTCGCGTTGTTCGTGAAATCTC-ACGGCTTAACTGTGAGCGTGCGGGCGATACGGGCAGACTGGAGTACTGCAGGGGAGACTGGAATTCCTGGTGTAGCGGTGGAATGCGCAGATATCAGGAGGAACACCGGTGGCGAAGGCGGGTCTCTGGGCAGTAACTGACGCTGAGGAGCGAAAGCGTGGGGAGCGAACAGGATTAGATACCCTGGTAGTCCACGCCGTAAACGGTGGGTACTAGGTGTGGGTTTCCTTCCTT--GGGATCCGTGCCGTAGCTAACGCATTAAGTACCCCGCCTGGGGAGTACGGCCGCAAGGCTAAAACTCAAAGGAATTGACGGGGGCCCGCACAAGCGGCGGAGCATGTGGATTAATTCGATGCAACGCGAAGAACCTTACCTGGGTTTGACATGCACAGGACGCGTCTAGAGATAGGCGTTCCCTTGTGGCCTGTGTGCAGGTGGTGCATGGCTGTCGTCAGCTCGTGTCGTGAGATGTTGGGTTAAGTCCCGCAACGAGCGCAACCCTTATCTTATGTTGCCAGCGGGTAATGCCGGGGACTCGTGAGAGACTGCCGGGGTCAACTCGGAGGAAGGTGGGGATGACGTCAAGTCATCATGCCCCTTATGTCCAGGGCTTCACACATGCTACAATGGCCGGTACAAAGGGCTGCGATGCCGCAAGGTTAAGCGAATCCTT-TTAAAGCCGGTCTCAGTTCGGATCGGGGTCTGCAACTCGACCCCGTGAAGTCGGAGTCGCTAGTAATCGCAGATCAGCAACGCTGCGGTGAATACGTTCCCGGGCCTTGTACACACCGCCCGTCACGTCATGAAAGTCGGTAACACCCGAAGCCAGTGGCCTAACCCT----TGGGAGGGAGCTGTCGAAGGTGGGATCGGCGATTGGGACGAAGTCGTAACAAGGTAGCCGTACCGGAAGGTGCGGCTGGATCACCTCCTTTCTAAGGAG>M._heidelbergense---------TTGTTTGGAGAGTTTGATCCTGGCTCAGGACGAACGCTGGCGGCGTGCTTAACACATGCAAGTCGAACGGAAAGGTCTCT------------T--C---------------GGAGATACTCGAGTGGCGAACGGGTGAGTAACACGTGGGTAATCTGCCCTGCACATC-GGGATAAGCCTGGGAAACTGGGTCTAATACCGAATAGGACCT-C-GAGGCGCATGCCT-T-GTGGTGGAAAG--CTT--T----T----GCGGTGTGGGATGGGCCCGCGGCCTATCAGCTTGTTGGTGGGGTGACGGCCTACCAAGGCGACGACGGGTAGCCGGCCTGAGAGGGTGTCCGGCCACACTGGGACTGAGATACGGCCCAGACTCCTACGGGAGGCAGCAGTGGGGAATATTGCACAATGGGCGCAAGCCTGATGCAGCGACGCCGCGTGGGGGATGACGGCCTTCGGGTTGTAAACCTCTTTCAGCAGGGACGAAGCGC----------------AAGTGACGGTACCTGCAGAAGAAGCACCGGCCAACTACGTGCCAGCAGCCGCGGTAATACGTAGGGTGCGAGCGTTGTCCGGAATTACTGGGCGTAAAGAGCTCGTAGGTGGTTTGTCGCGTTGTTCGTGAAAACCG-GGGGCTTAACCCTCGGCGTGCGGGCGATACGGGCAGACTGGAGTACTGCAGGGGAGACTGGAATTCCTGGTGTAGCGGTGGAATGCGCAGATATCAGGAGGAACACCGGTGGCGAAGGCGGGTCTCTGGGCAGTAACTGACGCTGAGGAGCGAAAGCGTGGGGAGCGAACAGGATTAGATACCCTGGTAGTCCACGCCGTAAACGGTGGGTACTAGGTGTGGGTTTCCTTCCTT--GGGATCCGTGCCGTAGCTAACGCATTAAGTACCCCGCCTGGGGAGTACGGCCGCAAGGCTAAAACTCAAAGGAATTGACGGGGGCCCGCACAAGCGGCGGAGCATGTGGATTAATTCGATGCAACGCGAAGAACCTTACCTGGGTTTGACATGCACAGGACGCCGGCAGAGATGTCGGTTCCCTTGTGGCCTGTGTGCAGGTGGTGCATGGCTGTCGTCAGCTCGTGTCGTGAGATGTTGGGTTAAGTCCCGCAACGAGCGCAACCCTTGTCTCATGTTGCCAGCGGGTAATGCCGGGGACTCGTGAGAGACTGCCGGGGTCAACTCGGAGGAAGGTGGGGATGACGTCAAGTCATCATGCCCCTTATGTCCAGGGCTTCACACATGCTACAATGGCCGGTACAAAGGGCTGCGATGCCGCAAGGTTAAGCGAATCCTT-TTAAAGCCGGTCTCAGTTCGGATCGGGGTCTGCAACTCGACCCCGTGAAGTCGGAGTCGCTAGTAATCGCAGATCAGCAACGCTGCGGTGAATACGTTCCCGGGCCTTGTACACACCGCCCGTCACGTCATGAAAGTCGGTAACACCCGAAGCCAGTGGCCTAACCCTT---TGGGAGGGAGCTGTCGAAGGTGGGATCGGCGATTGGGACGAAGTCGTAACAAGGTAGCCGTACCGGAAGGTGCGGCTGGATCACCTCCTTTCTAA---->M._interjectum----------TGTTTGGAGAGTTTGATCCTGGCTCAGGACGAACGCTGGCGGCGTGCTTAACACATGCAAGTCGAACGGAAAGGCCCCT------------T--C---------------GGGGGTACTCGAGTGGCGAACGGGTGAGTAACACGTGGGTAATCTGCCCTGCACTTC-GGGATAAGCCTGGGAAACTGGGTCTAATACCGGATAGGACCT-C-GAGGCGCATGCCT-T-GTGGTGGAAAG--CTT--T----T----GCGGTGTGGGATGGGCCCGCGGCCTATCAGCTAGTTGGTGGGGTGACGGCCTACCAAGGCGACGACGGGTAGCCGGCCTGAGAGGGTGTCCGGCCACACTGGGACTGAGATACGGCCCAGACTCCTACGGGAGGCAGCAGTGGGGAATATTGCACAATGGGCGCAAGCCTGATGCAGCGACGCCGCGTGGGGGATGACGGCCTTCGGGTTGTAAACCTCTTTCAGCAGGGACGAAGCGC----------------AAGTGACGGTACCTGCAGAAGAAGCACCGGCCAACTACGTGCCAGCAGCCGCGGTAATACGTAGGGTGCGAGCGTTGTCCGGAATTACTGGGCGTAAAGAGCTCGTAGGTGGTTTGTCGCGTTGTTCGTGAAATCTC-ACGGCTTAACTGTGAGCGTGCGGGCGATACGGGCAGACTGGAGTACTGCAGGGGAGACTGGAATTCCTGGTGTAGCGGTGGAATGCGCAGATATCAGGAGGAACACCGGTGGCGAAGGCGGGTCTCTGGGCAGTAACTGACGCTGAGGAGCGAAAGCGTGGGGAGCGAACAGGATTAGATACCCTGGTAGTCCACGCCGTAAACGGTGGGTACTAGGTGTGGGTTTCCTTCCTT--GGGATCCGTGCCGTAGCTAACGCATTAAGTACCCCGCCTGGGGAGTACGGCCGCAAGGCTAAAACTCAAAGGAATTGACGGGGGCCCGCACAAGCGGCGGAGCATGTGGATTAATTCGATGCAACGCGAAGAACCTTACCTGGGTTTGACATGCACAGGACGCCGGCAGAGATGTCGGTTCCCTTGTGGCCTGTGTGCAGGTGGTGCATGGCTGTCGTCAGCTCGTGTCGTGAGATGTTGGGTTAAGTCCCGCAACGAGCGCAACCCTTATTTCATGTTGCCAGCGGGTAATGCCGGGGACTCGTGAGAGACTGCCGGGGTCAACTCGGAGGAAGGTGGGGATGACGTCAAGTCATCATGCCCCTTATGTCCAGGGCTTCACACATGCTACAATGGCCGGTACAAAGGGCTGCGATGCCGCAAGGTTAAGCGAATCCTT-TTAAAGCCGGTCTCAGTTCGGATCGGGGTCTGCAACTCGACCCCGTGAAGTCGGAGTCGCTAGTAATCGCAGATCAGCAACGCTGCGGTGAATACGTTCCCGGGCCTTGTACACACCGCCCGTCACGTCATGAAAGTCGGTAACACCCGAAGCCAGTGGCCTAACCCTT---TGGGAGGGAGCTGTCGAAGGTGGGATCGGCGATTGGGACGAAGTCGTAACAAGGTAGCCGTACCGGAAGGTGCGGCTGGATCACCTCCTTT-------->M._intermedium----------TGTTTGGAGAGTTTGATCCTGGCTCAGGACGAACGCTGGCGGCGTGCTTAACACATGCAAGTCGAACGGAAAGGCCCCT------------T--C---------------GGGGGTACTCGAGTGGCGAACGGGTGAGTAACACGTGGGTAATCTGCCCTGCACACT-GGGATAAGCCTGGGAAACTGGGTCTAATACCGGATAGGACCT-C-TCGGCGCATGCCT-AGGAGGTGGAAAG--CTT--T----T----GCGGTGTGGGATGGGCCCGCGGCCTATCAGCTTGTTGGTGGGGTGACGGCCTACCAAGGCGACGACGGGTAGCCGGCCTGAGAGGGTGTCCGGCCACACTGGGACTGAGATACGGCCCAGACTCCTACGGGAGGCAGCAGTGGGGAATATTGCACAATGGGCGCAAGCCTGATGCAGCGACGCCGCGTGGGGGATGACGGCCTTCGGGTTGTAAACCTCTTTCAGCAGGGACGAAGCGC----------------AAGTGACGGTACCTGCAGAAGAAGCACCGGCCAACTACGTGCCAGCAGCCGCGGTAATACGTAGGGTGCGAGCGTTGTCCGGAATTACTGGGCGTAAAGAGCTCGTAGGTGGTTTGTCGCGTTGTTCGTGAAATCTC-ACGGCTTAACTGTGAGCGTGCGGGCGATACGGGCAGACTAGAGTACTGCAGGGGAGACTGGAATTCCTGGTGTAGCGGTGGAATGCGCAGATATCAGGAGGAACACCGGTGGCGAAGGCGGGTCTCTGGGCAGTAACTGACGCTGAGGAGCGAAAGCGTGGGGAGCGAACAGGATTAGATACCCTGGTAGTCCACGCCGTAAACGGTGGGTACTAGGTGTGGGTTTCCTTCCTT--GGGATCCGTGCCGTAGCTAACGCATTAAGTACCCCGCCTGGGGAGTACGGCCGCAAGGCTAAAACTCAAAGGAATTGACGGGGGCCCGCACAAGCGGCGGAGCATGTGGATTAATTCGATGCAACGCGAAGAACCTTACCTGGGTTTGACATGCACAGGACGCGTCTAGAGATAGGCGTTCCCTTGTGGCCTGTGTGCAGGTGGTGCATGGCTGTCGTCAGCTCGTGTCGTGAGATGTTGGGTTAAGTCCCGCAACGAGCGCAACCCTTGTCTCATGTTGCCAGCGGGTAATGCCGGGGACTCGTGAGAGACTGCCGGGGTCAACTCGGAGGAAGGTGGGGATGACGTCAAGTCATCATGCCCCTTATGTCCAGGGCTTCACACATGCTACAATGGCCGGTACAAAGGGCTGCGATGCCGCGAGGTTAAGCGAATCCTT-TTAAAGCCGGTCTCAGTTCGGATCGGGGTCTGCAACTCGACCCCGTGAAGTCGGAGTCGCTAGTAATCGCAGATCAGCAACGCTGCGGTGAATACGTTCCCGGGCCTTGTACACACCGCCCGTCACGTCATGAAAGTCGGTAACACCCGAAGCCAGTGGCCTAACCCTT---TGGGAGGGAGCTGTCGAAGGTGGGATCGGCGATTGGGACGAAGTCGTAACAAGGTAGCCGTACCGGAAGGTGCGGCTGGATCACCTCCTTT-------->M._intracellulare---------TTGTTTGGAGAGTTTGATCCTGGCTCAGGACGAACGCTGGCGGCGTGCTTAACACATGCAAGTCGAACGGAAAGGCCCCT------------T--C---------------GGGGGTACTCGAGTGGCGAACGGGTGAGTAACACGTGGGCAATCTGCCCTGCACTTC-GGGATAAGCCTGGGAAACTGGGTCTAATACCGGATAGGACCT-T-TAGGCGCATGTCT-T-TAGGTGGAAAG--CTT--T----T----GCGGTGTGGGATGGGCCCGCGGCCTATCAGCTTGTTGGTGGGGTGATGGCCTACCAAGGCGACGACGGGTAGCCGGCCTGAGAGGGTGTCCGGCCACACTGGGACTGAGATACGGCCCAGACTCCTACGGGAGGCAGCAGTGGGGAATATTGCACAATGGGCGCAAGCCTGATGCAGCGACGCCGCGTGGGGGATGACGGCCTTCGGGTTGTAAACCTCTTTCACCATCGACGAAGGTCCGG-GTTT--TCTC-GGATTGACGGTAGGTGGAGAAGAAGCACCGGCCAACTACGTGCCAGCAGCCGCGGTAATACGTAGGGTGCGAGCGTTGTCCGGAATTACTGGGCGTAAAGAGCTCGTAGGTGGTTTGTCGCGTTGTTCGTGAAATCTC-ACGGCTTAACTGTGAGCGTGCGGGCGATACGGGCAGACTAGAGTACTGCAGGGGAGACTGGAATTCCTGGTGTAGCGGTGGAATGCGCAGATATCAGGAGGAACACCGGTGGCGAAGGCGGGTCTCTGGGCAGTAACTGACGCTGAGGAGCGAAAGCGTGGGGAGCGAACAGGATTAGATACCCTGGTAGTCCACGCCGTAAACGGTGGGTACTAGGTGTGGGTTTCCTTCCTT--GGGATCCGTGCCGTAGCTAACGCATTAAGTACCCCGCCTGGGGAGTACGGCCGCAAGGCTAAAACTCAAAGGAATTGACGGGGGCCCGCACAAGCGGCGGAGCATGTGGATTAATTCGATGCAACGCGAAGAACCTTACCTGGGTTTGACATGCACAGGACGCGTCTAGAGATAGGCGTTCCCTTGTGGCCTGTGTGCAGGTGGTGCATGGCTGTCGTCAGCTCGTGTCGTGAGATGTTGGGTTAAGTCCCGCAACGAGCGCAACCCTTGTCTCATGTTGCCAGCGGGTAATGCCGGGGACTCGTGAGAGACTGCCGGGGTCAACTCGGAGGAAGGTGGGGATGACGTCAAGTCATCATGCCCCTTATGTCCAGGGCTTCACACATGCTACAATGGCCGGTACAAAGGGCTGCGATGCCGCAAGGTTAAGCGAATCCTT-TTAAAGCCGGTCTCAGTTCGGATTGGGGTCTGCAACTCGACCCCATGAAGTCGGAGTCGCTAGTAATCGCAGATCAGCAACGCTGCGGTGAATACGTTCCCGGGCCTTGTACACACCGCCCGTCACGTCATGAAAGTCGGTAACACCCGAAGCCAGTGGCCTAACCCT----TGGGAGGGAGCTGTCGAAGGTGGGATCGGCGATTGGGACGAAGTCGTAACAAGGTAGCCGTACCGGAAGGTGCGGCTGGATCACCTCCTTTCTAAGGAG>M._kansasii----------TGTTTGGAGAGTTTGATCCTGGCTCAGGACGAACGCTGGCGGCGTGCTTAACACATGCAAGTCGAACGGAAAGGTCTCT------------T--C---------------GGAGACACTCGAGTGGCGAACGGGTGAGTAACACGTGGGCAATCTGCCCTGCACACC-GGGATAAGCCTGGGAAACTGGGTCTAATACCGGATAGGACCA-C-TTGGCGCATGCCT-T-GTGGTGGAAAG--CTT--T----T----GCGGTGTGGGATGGGCCCGCGGCCTATCAGCTTGTTGGTGGGGTGACGGCCTACCAAGGCGACGACGGGTAGCCGGCCTGAGAGGGTGTCCGGCCACACTGGGACTGAGATACGGCCCAGACTCCTACGGGAGGCAGCAGTGGGGAATATTGCACAATGGGCGCAAGCCTGATGCAGCGACGCCGCGTGGGGGATGACGGCCTTCGGGTTGTAAACCTCTTTCACCATCGACGAAGGTCCGG-GTTC--TCTC-GGATTGACGGTAGGTGGAGAAGAAGCACCGGCCAACTACGTGCCAGCAGCCGCGGTAATACGTAGGGTGCGAGCGTTGTCCGGAATTACTGGGCGTAAAGAGCTCGTAGGTGGTTTGTCGCGTTGTTCGTGAAATCTC-ACGGCTTAACTGTGAGCGTGCGGGCGATACGGGCAGACTAGAGTACTGCAGGGGAGACTGGAATTCCTGGTGTAGCGGTGGAATGCGCAGATATCAGGAGGAACACCGGTGGCGAAGGCGGGTCTCTGGGCAGTAACTGACGCTGAGGAGCGAAAGCGTGGGGAGCGAACAGGATTAGATACCCTGGTAGTCCACGCCGTAAACGGTGGGTACTAGGTGTGGGTTTCCTTCCTT--GGGATCCGTGCCGTAGCTAACGCATTAAGTACCCCGCCTGGGGAGTACGGCCGCAAGGCTAAAACTCAAAGGAATTGACGGGGGCCCGCACAAGCGGCGGAGCATGTGGATTAATTCGATGCAACGCGAAGAACCTTACCTGGGTTTGACATGCACAGGACGCGTCTAGAGATAGGCGTTCCCTTGTGGCCTGTGTGCAGGTGGTGCATGGCTGTCGTCAGCTCGTGTCGTGAGATGTTGGGTTAAGTCCCGCAACGAGCGCAACCCTTGTCTCATGTTGCCAGCGGGTAATGCCGGGGACTCGTGAGAGACTGCCGGGGTCAACTCGGAGGAAGGTGGGGATGACGTCAAGTCATCATGCCCCTTATGTCCAGGGCTTCACACATGCTACAATGGCCGGTACAAAGGGCTGCGATGCCGCGAGGTTAAGCGAATCCTT-TTAAAGCCGGTCTCAGTTCGGATCGGGGTCTGCAACTCGACCCCGTGAAGTCGGAGTCGCTAGTAATCGCAGATCAGCAACGCTGCGGTGAATACGTTCCCGGGCCTTGTACACACCGCCCGTCACGTCATGAAAGTCGGTAACACCCGAAGCCAGTGGCCTAACCCT----CGGGAGGGAGCTGTCGAAGGTGGGATCGGCGATTGGGACGAAGTCGTAACAAGGTAGCCGTACCGGAAGGTGCGGCTGGATCACCTCCTTT-------->M._kubicae---------TTGTTTGGAGAGTTTGATCCTGGCTCAGGACGAACGCTGGCGGCGTGCTTAACACATGCAAGTCGAACGGAAAGGCCCCT------------T--C---------------GGGGGTACTCGAGTGGCGAACGGGTGAGTAACACGTGGGTGATCTACCCTGCACTTC-GGGATAAGCCTGGGAAACTGGGTCTAATACCGGATAGGACCA-T-GAGATGCATGTCT-T-ATGGTGGAAAG--CTT--T----T----GCGGTGTGGGATGGGCCCGCGGCCTATCAGCTTGTTGGTGGGGTGACGGCCTACCAAGGCGACGACGGGTAGCCGGCCTGAGAGGGTGTCCGGCCACACTGGGACTGAGATACGGCCCAGACTCCTACGGGAGGCAGCAGTGGGGAATATTGCACAATGGGCGCAAGCCTGATGCAGCGACGCCGCGTGGGGGATGACGGCCTTCGGGTTGTAAACCTCTTTCAGCAGGGACGAAGCGC----------------AAGTGACGGTACCTGCAGAAGAAGCACCGGCCAACTACGTGCCAGCAGCCGCGGTAATACGTAGGGTGCGAGCGTTGTCCGGAATTACTGGGCGTAAAGAGCTCGTAGGTGGTTTGTCGCGTTGTTCGTGAAAACCG-GGGGCTTAACCCTCGGCGTGCGGGCGATACGGGCAGACTGGAGTACTGCAGGGGAGACTGGAATTCCTGGTGTAGCGGTGGAATGCGCAGATATCAGGAGGAACACCGGTGGCGAAGGCGGGTCTCTGGGCAGTAACTGACGCTGAGGAGCGAAAGCGTGGGGAGCGAACAGGATTAGATACCCTGGTAGTCCACGCCGTAAACGGTGGGTACTAGGTGTGGGTTTCCTTCCTT--GGGATCCGTGCCGTAGCTAACGCATTAAGTACCCCGCCTGGGGAGTACGGCCGCAAGGCTAAAACTCAAAGGAATTGACGGGGGCCCGCACAAGCGGCGGAGCATGTGGATTAATTCGATGCAACGCGAAGAACCTTACCTGGGTTTGACATGCACAGGACGCGTCTAGAGATAGGCGTTCCCTTGTGGCCTGTGTGCAGGTGGTGCATGGCTGTCGTCAGCTCGTGTCGTGAGATGTTGGGTTAAGTCCCGCAACGAGCGCAACCCTTGTCTCATGTTGCCAGCGGGTAATGCCGGGGACTCGTGAGAGACTGCCGGGGTCAACTCGGAGGAAGGTGGGGATGACGTCAAGTCATCATGCCCCTTATGTCCAGGGCTTCACACATGCTACAATGGCCGGTACAAAGGGCTGCGATGCCGCGAGGTTAAGCGAATCCTT-TTAAAGCCGGTCTCAGTTCGGATCGGGGTCTGCAACTCGACCCCGTGAAGTCGGAGTCGCTAGTAATCGCAGATCAGCAACGCTGCGGTGAATACGTTCCCGGGCCTTGTACACACCGCCCGTCACGTCATGAAAGTCGGTAACACCCGAAGCCAGTGGCCTAACCCTT---TGGGAGGGAGCTGTCGAAGGTGGGATCGGCGATTGGGACGAAGTCGTAACAAGGTAGCCGTACCGGAAGGTGCGGCTGGATCACCTCCTTTCTAA---->M._kyorinense----------TGTTTGGAGAGTTTGATCCTGGCTCAGGACGAACGCTGGCGGCGTGCTTAACACATGCAAGTCGAACGGAAAGGCCCCT------------TT-CG--------------GGGGGTGCTCGAGTGGCGAACGGGTGAGTAACACGTGGGTGATCTGCCCTGCACTTC-GGGATAAGCTTGGGAAACTGGGTCTAATACCGGATAGGACTC-C-GAGATGCATGTCT-T-GGGGTGGAAAG--CTT--T----T----GCGGTGTGGGATGGGCCCGCGGCCTATCAGCTTGTTGGTGGGGTGATGGCCTACCAAGGCGACGACGGGTAGCCGGCCTGAGAGGGTGTCCGGCCACACTGGGACTGAGATACGGCCCAGACTCCTACGGGAGGCAGCAGTGGGGAATATTGCACAATGGGCGCAAGCCTGATGCAGCGACGCCGCGTGGGGGATGACGGCCTTCGGGTTGTAAACCTCTTTCACCATCGACGAAGCTTCGG-GTTT--TCTC-GGGGTGACGGTAGGTGGAGAAGAAGCACCGGCCAACTACGTGCCAGCAGCCGCGGTAATACGTAGGGTGCGAGCGTTGTCCGGAATTACTGGGCGTAAAGAGCTCGTAGGTGGTTTGTCGCGTTGTTCGTGAAATTCC-CTGGCTTAACTGGGGGCGTGCGGGCGATACGGGCAGACTGGAGTACTGCAGGGGAGACTGGAATTCCTGGTGTAGCGGTGGAATGCGCAGATATCAGGAGGAACACCGGTGGCGAAGGCGGGTCTCTGGGCAGTAACTGACGCTGAGGAGCGAAAGCGTGGGGAGCGAACAGGATTAGATACCCTGGTAGTCCACGCCGTAAACGGTGGGTACTAGGTGTGGGTTTCCTTCCTT--GGGATCCGTGCCGTAGCTAACGCATTAAGTACCCCGCCTGGGGAGTACGGCCGCAAGGCTAAAACTCAAAGGAATTGACGGGGGCCCGCACAAGCGGCGGAGCATGTGGATTAATTCGATGCAACGCGAAGAACCTTACCTGGGTTTGACATGCACAGGACGCCGGCAGAGATGTCGGTTCCCTTGTGGTCTGTGTGCAGGTGGTGCATGGCTGTCGTCAGCTCGTGTCGTGAGATGTTGGGTTAAGTCCCGCAACGAGCGCAACCCTTGTCTCATGTTGCCAGCGCGTGATGGCGGGGACTCGTGAGAGACTGCCGGGGTCAACTCGGAGGAAGGTGGGGATGACGTCAAGTCATCATGCCCCTTATGTCCAGGGCTTCACACATGCTACAATGGCCGGTACAAAGGGCTGCGATGCCGTGAGGTTTAGCGAATCCTT--TAAAGCCGGTCTCAGTTCGGATCGGGGTCTGCAACTCGACCCCGTGAAGTCGGAGTCGCTAGTAATCGCAGATCAGCAATGCTGCGGTGAATACGTTCCCGGGCCTTGTACACACCGCCCGTCACGTCATGAAAGTCGGTAACACCCGAAGCCCATGGCCTAACCCGC--AAGGGAGGGAGTGGTCGAAGGTGGGATCGGCGATTGGGACGAAGTCGTAACAAGGTAGCCGTACCGGAAGGTGCGGCTGGATCACCTCCTTT-------->M._lacus---------TTGTTTGGAGAGTTTGATCCTGGCTCAGGACGAACGCTGGCGGCGTGCTTAACACATGCAAGTCGAACGGAAAGGTCTCT------------T--C---------------GGAGATACTCGAGTGGCGAACGGGTGAGTAACACGTGGGTGATCTGCCCTGCACTTC-GGGATAAGCCTGGGAAACTGGGTCTAATACCGGATAGGACCA-T-GAGATTCATGTCT-T-GTGGTGGAAAG--CTT--T----T----GCGGTGTGGGATGAGCCCGCGGCCTATCAGCTTGTTGGTGGGGTGACGGCCTACCAAGGCGACGACGGGTAGCCGGCCTGAGAGGGTGTCCGGCCACACTGGGACTGAGATACGGCCCAGACTCCTACGGGAGGCAGCAGTGGGGAATATTGCACAATGGGCGCAAGCCTGATGCAGCGACGCCGCGTGGGGGATGACGGCCTTCGGGTTGTAAACCTCTTTCACCATCGACGAAGGTCCGG-GTTT--TCTC-GGATTGACGGTAGATGGAGAAGAAGCACCGGCCAACTACGTGCCAGCAGCCGCGGTAATACGTAGGGTGCGAGCGTTGTCCGGAATTACTGGGCGTAAAGAGCTCGTAGGTGGTTTGTCGCGTTGTTCGTGAAATCTC-ACGGCTTAACTGTGAGCGTGCGGGCGATACGGGCAGACTGGAGTACTGCAGGGGAGACTGGAATTCCTGGTGTAGCGGTGGAATGCGCAGATATCAGGAGGAACACCGGTGGCGAAGGCGGGTCTCTGGGCAGTAACTGACGCTGAGGAGCGAAAGCGTGGGGAGCGAACAGGATTAGATACCCTGGTAGTCCACGCCGTAAACGGTGGGTACTAGGTGTGGGTTTCCTTCCTT--GGGATCCGTGCCGTAGCTAACGCATTAAGTACCCCGCCTGGGGAGTACGGCCGCAAGGCTAAAACTCAAAGGAATTGACGGGGGCCCGCACAAGCGGCGGAGCATGTGGATTAATTCGATGCAACGCGAAGAACCTTACCTGGGTTTGACATGCACAGGACGCGTCTAGAGATAGGCGTTCCCTTGTGGCCTGTGTGCAGGTGGTGCATGGCTGTCGTCAGCTCGTGTCGTGAGATGTTGGGTTAAGTCCCGCAACGAGCGCAACCCTTGTCTCATGTTGCCAGCGGGTAATGCCGGGGACTCGTGAGAGACTGCCGGGGTCAACTCGGAGGAAGGTGGGGATGACGTCAAGTCATCATGCCCCTTATGTCCAGGGCTTCACACATGCTACAATGGCCGGTACAAAGGGCTGCGATGCCGCGAGGTTAAGCGAATCCTT-TTAAAGCCGGTCTCAGTTCGGATCGGGGTCTGCAACTCGACCCCGTGAAGTCGGAGTCGCTAGTAATCGCAGATCAGCAACGCTGCGGTGAATACGTTCCCGGGCCTTGTACACACCGCCCGTCACGTCATGAAAGTCGGTAACACCCGAAGCCAGTGGCCTAACCCTT---TGGGAGGGAGCTGTCGAAGGTGGGATCGGCGATTGGGACGAAGTCGTAACAAGGTAGCCGTACCGGAAGGTGCGGCTGGATCACCTCCTTTCTAAGGAG>M._lentiflavum----------TGTTTGGAGAGTTTGATCCTGGCTCAGGACGAACGCTGGCGGCGTGCTTAACACATGCAAGTCGAACGGAAAGGCCTCT------------T--C---------------GGAGGTACTCGAGTGGCGAACGGGTGAGTAACACGTGGGTAATCTGCCCTGCACTTC-GGGATAAGCCTGGGAAACTGGGTCTAATACCGGATAGGACCT-T-TTGGCGCATGCCT-T-TTGGTGGAAAG--CTT--T----T----GCGGTGTGGGATGGGCCCGCGGCCTATCAGCTTGTTGGTGGGGTGACGGCCTACCAAGGCGACGACGGGTAGCCGGCCTGAGAGGGTGTCCGGCCACACTGGGACTGAGATACGGCCCAGACTCCTACGGGAGGCAGCAGTGGGGAATATTGCACAATGGGCGCAAGCCTGATGCAGCGACGCCGCGTGGGGGATGACGGCCTTCGGGTTGTAAACCTCTTTCAGCAGGGACGAAGCGC----------------AAGTGACGGTACCTGCAGAAGAAGCACCGGCCAACTACGTGCCAGCAGCCGCGGTAATACGTAGGGTGCGAGCGTTGTCCGGAATTACTGGGCGTAAAGAGCTCGTAGGTGGTTTGTCGCGTTGTTCGTGAAAACCG-GGGGCTTAACCCTCGGCGTGCGGGCGATACGGGCAGACTGGAGTACTGCAGGGGAGACTGGAATTCCTGGTGTAGCGGTGGAATGCGCAGATATCAGGAGGAACACCGGTGGCGAAGGCGGGTCTCTGGGCAGTAACTGACGCTGAGGAGCGAAAGCGTGGGGAGCGAACAGGATTAGATACCCTGGTAGTCCACGCCGTAAACGGTGGGTACTAGGTGTGGGTTTCCTTCCTT--GGAATCCGTGCCGTAGCTAACGCATTAAGTACCCCGCCTGGGGAGTACGGCCGCAAGGCTAAAACTCAAAGGAATTGACGGGGGCCCGCACAAGCGGCGGAGCATGTGGATTAATTCGATGCAACGCGAAGAACCTTACCTGGGTTTGACATGCACAGGACGCCGGCAGAGATGTCGGTTCCCTTGTGGCCTGTGTGCAGGTGGTGCATGGCTGTCGTCAGCTCGTGTCGTGAGATGTTGGGTTAAGTCCCGCAACGAGCGCAACCCTTGTCTCATGTTGCCAGCGGGTAATGCCGGGGACTCGTGAGAGACTGCCGGGGTCAACTCGGAGGAAGGTGGGGATGACGTCAAGTCATCATGCCCCTTATGTCCAGGGCTTCACACATGCTACAATGGCCGGTACAAAGGGCTGCGATGCCGTAAGGTTAAGCGAATCCTT-TTAAAGCCGGTCTCAGTTCGGATCGGGGTCTGCAACTCGACCCCGTGAAGTCGGAGTCGCTAGTAATCGCAGATCAGCAACGCTGCGGTGAATACGTTCCCGGGCCTTGTACACACCGCCCGTCACGTCATGAAAGTCGGTAACACCCGAAGCCAGTGGCCTAACCTT----TTGGAGGGAGCTGTCGAAGGTGGGATCGGCGATTGGGACGAAGTCGTAACAAGGTAGCCGTACCGGAAGGTGCGGCTGGATCACCTCCTTT-------->M._leprae---------TTGTTTGGAGAGTTTGATCCTGGCTCAGGACGAACGCTGGCGGCGTGCTTAACACATGCAAGTCGAACGGAAAGGTCTCTAAAAAATCTTTTT--T---------------AGAGATACTCGAGTGGCGAACGGGTGAGTAACACGTGGGTAATCTGCCCTGCACTTCAGGGATAAGCTTGGGAAACTGGGTCTAATACCGGATAGGACTT-C-AAGGCGCATGTCT-T-GTGGTGGAAAG--CTT-TT----T----GCGGTGCAGGATGGGCCCGCGGCTTATCAGCTTGTTGGTGGGGTGACGGCCTACCAAGGCGACGACGGGTAGCCGGCCTGAGAGGGTGTACGGCCACACTGGGACTGAGATACGGCCCAGACTCCTACGGGAGGCAGCAGTGGGGAATATTGCACAATGGGCGCAAGCCTGATGCAGCGACGCCGCGTGGGGGATGACGGCTTTCGGGTTGTAAACCTCTTTCACCATCGACGAAGGTCTGG-GTTT--TCTC-GGATTGACGGTAGGTGGAGAAGAAGCACCGGCCAACTACGTGCCAGCAGCCGCGGTAATACGTAGGGTGCGAGCGTTGTCCGGAATTACTGGGCGTAAAGAGCTCGTAGGTGGTTTGTCGCGTTGTTCGTGAAATCTC-ACGGCTTAACTGTGAGCGTGCGGGCGATACGGGCAGACTGGAGTACTGCAGGGGAGACTGGAATTCCTGGTGTAGCGGTGGAATGCGCAGATATCAGGAGGAACACCAGTGGCGAAGGCGGGTCTCTGGGCAGTAACTGACGCTGAGGAGCGAAAGCGTGGGGAGCAAACAGGATTAGATACCCTGGTAGTCCACGCCGTAAACGGTGGGTACTAGGTGTGGGTTTCCTTCCTT--GGGATCCGTGCCGTAGCTAACGCATTAAGTACCCCGCCTGGGGAGTACGGCCGCAAGGCTAAAACTCAAAGGAATTGACGGGGGCCCGCACAAGCGGCGGAGCATGTGGATTAATTCGATGCAACGCGAAGAACCTTACCTGGGTTTGACATGCACAGGATGCGTCTAGAGATAGGCACTCCCTTGTGGCCTGTGTGCAGGTGGTGCATGGCTGTCGTCAGCTCGTGTCGTGAGATGTTGGGTTAAGTCCCGCAACGAGCGCAACCCTTGTCTCATGTTGCCAGCACGTAATGGTGGGGACTCGTGAGAGACTGCCGCGGTCAACTCGGAGGAAGGTGGGGATGACGTCAAGTCATCATGCCCCTTATGTCCAGGGCTTCACACATGCTACAATGGCCGGTACAAAGGGCTGCGATGCCGCAAGGTTAAGCGAATCCTT-TTAAAGCCGGTCTCAGTTCGGATCGGGGTCTGCAACTCGACCCCGTGAAGTCGGAGTCGCTAGTAATCGCAGATCAGCAACGCTGCGGTGAATACGTTCCCGGGCCTTGTACACACCGCCCGTCACGTCATGAAAGTCGGTAACACCCGAAGCCAGTGGCCTAACCCT----CGGGAGGGAGCTGTCCAAGGTGGGATCGGCGATTGGGACGAAGTCGTAACAAGGTAGCCGTACCGGAAGGTGCGGCTGGATCACCTCCTTTCTAAGGAG>M._lepraemurium---------TTGCTTGGAGAGTTTGATCCTGGCTCAGGACGAACGCTGGCGGCGTGCTTAACACATGCAAGTCGAACGGAAAGGCCTCT------------T--C---------------GGAGGTACTCGAGTGGCGAACGGGTGAGTAACACGTGGGCAATCTGCCCTGCACTTC-GGGATAAGCCTGGGAAACTGGGTCTAATACCGGATAGGACCT-C-AAGACGCATGTCT-T-CTGGTGGAAAG--CTT--T----T----GCGGTGTGGGATGGGCCCGCGGCCTATCAGCTTGTTGGTGGGGTGACGGCCTACCAAGGCGACGACGGGTAGCCGGCCTGAGAGGGTGTCCGGCCACACTGGGACTGAGATACGGCCCAGACTCCTACGGGAGGCAGCAGTGGGGAATATTGCACAATGGGCGCAGGCCTGATGCAGCGACGCCGCGTGGGGGATGACGGCCTTCGGGTTGTAAACCTCTTTCACTATCGACGAAGGTCCGG-GTTT--TCTC-GGATTGACGGTAGGTGGAGAAGAAGCACCGGCCAACTACGTGCCAGCAGCCGCGGTAATACGTAGGGTGCGAGCGTTGTCCGGAATTACTGGGCGTAAAGAGCTCGTAGGTGGTTTGTCGCGTTGTTCGTGAAATCTC-ACGGCTTAACTGTGAGCGTGCGGGCGATACGGGCAGACTAGAGTACTGCAGGGGGGACTGGAATTCCTGGTGTAGCGGTGGAATGCGCAGATATCAGGAGGAACACCGGTGGCGAAGGCGGGTCTCTGGGCAGTAACTGACGCTGAGGAGCGAAAGCGTGGGGAGCGAACAGGATTAGATACCCTGGTAGTCCACGCCGTAAACGGTGGGTACTAGGTGTGGGTTTCCTTCCTT--GGGATCCGTGCCGTAGCTAACGCATTAAGTACCCCGCCTGGGGAGTACGGCCGCAAGGCTAAAACTCAAAGGAATTGACGGGGGCCCGCACAAGCGGCGGAGCATGTGGATTAATTCGATGCAACGCGAAGAACCTTACCTGGGTTTGACATGCACAGGACGCGTCTAGAGATAGGCGTTCCCTTGTGGCCTGTGTGCAGGTGGTGCATGGCTGTCGTCAGCTCGTGTCGTGAGATGTTGGGTTAAGTCCCGCAACGAGCGCAACCCTTGTCTCATGTTGCCAGCGGGTAATGCCGGGGACTCGTGAGAGACTGCCGGGGTCAACTCGGAGGAAGGTGGGGATGACGTCAAGTCATCATGCCCCTTATGTCCAGGGCTTCACACATGCTACAATGGCCGGTACAAAGGGCTGCGATGCCGTAAGGTTAAGCGAATCCTT-TTAAAGCCGGTCTCAGTTCGGATTGGGGTCTGCAACTCGACCCCATGAAGTCGGAGTCGCTAGTAATCGCAGATCAGCAACGCTGCGGTGAATACGTTCCCGGGCCTTGTACACACCGCCCGTCACGTCATGAAAGTCGGTAACACCCGAAGCCAGTCGCCTAACCCTT--TTGGGAGGGAGCTGTCGAAGGTGGGATCGGCGATTGGGACGAAGTCGTAACAAGGTAGCCGTACCGGAAGGTGCGGCTGGATCACCTCCTTTCTAAGGAG>M._lepromatosis---------TTGTTTGGAGAGTTTGATCCTGGCTCAGGACGAACGCTGGCGGCGTGCTTAACACATGCAAGTCGAACGGAAAGGTCTCT------------T--AATACTTAAACCTATTAAAGATACTCGAGTGGCGAACGGGTGAGTAACACGTGGGTAATCTACCCTGCACTTCAGGGATAAGCCTGGGAAACCGGGTCTAATACCGGATAGGACTT-C-AAGGCGCATGTCT-T-GTGGTGGAAAG--CTT--T----T----GCGGTGCGGGATGGGCCCGCGGCCTATCAGCTTGTTGGTGGGGTGACGGCCTACCAAGGCGACGACGGGTAGCCGGCCTGAGAGGGTGTCCGGCCACACTGGGACTGAGATACGGCCCAGACTCCTACGGGAGGCAGCAGTGGGGAATATTGCACAATGGGCGCAAGCCTGATGCAGCGACGCCGCGTGGGGGATGACGGCTTTCGGGTTGTAAACCTCTTTCACCATTGACGAAGATCTGG-GTTT--TCTC-GGATTGACGGTAGGTGGAGAAGAAGCACCGGCCAACTACGTGCCAGCAGCCGCGGTAATACGTAGGGTGCGAGCGTTGTCCGGAATTACTGGGCGTAAAGAGCTCGTAGGTGGTTTGTCGCGTTGTTCGTGAAATCTC-ACGGCTTAACTGTGAGCGTGCGGGCGATACGGGCAGACTGGAGTACTGCAGGGGAGACTGGAATTCCTGGTGTAGCGGTGGAATGCGCAGATATCAGGAGGAACACCGGTGGCGAAGGCGGGTCTCTGGGCAGTAACTGACGCTGAGGAGCGAAAGCGTGGGGAGCGAACAGGATTAGATACCCTGGTAGTCCACGCCGTAAACGGTGGGTACTAGGTGTGGGTTTCCTTCCTT--GGGATCCGTGCCGTAGCTAACGCATTAAGTACCCCGCCTGGGGAGTACGGCCGCAAGGCTAAAACTCAAAGGAATTGACGG-GGCCCGCACAAGCGGCGGAGCATGTGGATTAATTCGATGCAACGCGAAGAACCTTACCTGGGTTTGACATGCACAGGACGCGTCTAGAGATAGGCGTTCCTTTTTGGCCTGTGTGCAGGTGGTGCATGGCTGTCGTCAGCTCGTGTCGTGAGATGTTGGGTTAAGTCCCGCAACGAGCGCAACCCTTGTCTCATGTTGCCAGCACGTAATGGTGGGGACTCGTGAGAGACTGCCGCGGTCAACTCGGAGGAAGGTGGGGATGACGTCAAGTCATCATGCCCCTTATGTCCAGGGCTTCACACATGCTACAATGGCCGGTACAAAGGGCTGCGATGCCGCAAGGTTAAGCGAATCCTTATTAAAGCCGGTCTCAGTTCGGATCGGGGTCTGCAACTCGACCCCGTGAAGCCGGAGTCGCTAGTAATCGCAGATCAGCAACGCTGCGGTGAATACGTTCCCGGGCCTTGTACACACCGCCCGTCACGTCATGAAAGTCGGTAACACCCGAAGCCAGTGGCCTAACCCT----CGGGAGGGAGCTGTCGAAGGTGGGATCGGCGATTGGGACGAAGTCGTAACAAGGTAGCCGTACCGGAAGGTGCGGCTGGATCACCTCCTTTCTAAGGAG>M._liflandiiT------TTTTGTTTGGAGAGTTTGATCCTGGCTCAGGACGAACGCTGGCGGCGTGCTTAACACATGCAAGTCGAACGGAAAGGTCTCT------------T--C---------------GGAGACACTCGAGTGGCGAACGGGTGAGTAACACGTGGGCGATCTGCCCTGCACTTC-GGGATAAGCCTGGGAAACTGGGTCTAATACCGGATAGGACCA-C-GGGATTCATGTCC-T-GTGGTGGAAAG--CTT--T----T----GCGGTGTGGGATGGGCCCGCGGCCTATCAGCTTGTTGGTGGGGTAACGGCCTACCAAGGCGACGACGGGTAGCCGGCCTGAGAGGGTGTCCGGCCACACTGGGACTGAGATACGGCCCAGACTCCTACGGGAGGCAGCAGTGGGGAATATTGCACAATGGGCGGAAGCCTGATGCAGCGACGCCGCGTGGGGGATGACGGCCTTCGGGTTGTAAACCTCTTTCACCATCGACGAAGGTTCGG-GTTT--TCTC-GGATTGACGGTAGATGGAGAAGAAGCACCGGCCAACTACGTGCCAGCAGCCGCGGTAATACGTAGGGTGCGAGCGTTGTCCGGAATTACTGGGCGTAAAGAGCTCGTAGGTGGTTTGTCGCGTTGTTCGTGAAATCTC-ACGGCTTAACTGTGAGCGTGCGGGCGATACGGGCAGACTAGAGTACTGCAGGGGAGACTGGAATTCCTGGTGTAGCGGTGGAATGCGCAGATATCAGGAGGAACACCGGTGGCGAAGGCGGGTCTCTGGGCAGTAACTGACGCTGAGGAGCGAAAGCGTGGGGAGCGAACAGGATTAGATACCCTGGTAGTCCACGCCGTAAACGGTGGGTACTAGGTGTGGGTTTCCTTCCTT--GGGATCCGTGCCGTAGCTAACGCATTAAGTACCCCGCCTGGGGAGTACGGCCGCAAGGCTAAAACTCAAAGGAATTGACGGGGGCCCGCACAAGCGGCGGAGCATGTGGATTAATTCGATGCAACGCGAAGAACCTTACCTGGGTTTGACATGCACAGGACGCGTCTAGAGATAGGCGTTCCCTTGTGGCCTGTGTGCAGGTGGTGCATGGCTGTCGTCAGCTCGTGTCGTGAGATGTTGGGTTAAGTCCCGCAACGAGCGCAACCCTTGTCTCATGTTGCCAGCACGTAATGGTGGGGACTCGTGAGAGACTGCCGGGGTCAACTCGGAGGAAGGTGGGGATGACGTCAAGTCATCATGCCCCTTATGTCCAGGGCTTCACACATGCTACAATGGCCGGTACAAAGGGCTGCGATGCCGCGAGGTTAAGCGAATCCTT--TAAAGCCGGTCTCAGTTCGGATCGGGGTCTGCAACTCGACCCCGTGAAGTCGGAGTCGCTAGTAATCGCAGATCAGCAACGCTGCGGTGAATACGTTCCCGGGCCTTGTACACACCGCCCGTCACGTCATGAAAGTCGGTAACACCCGAAGCCAGTGGCCTAACCCTT---TGGGAGGGAGCTGTCGAAGGTGGGATCGGCGATTGGGACGAAGTCGTAACAAGGTAGCCGTACCGGAAGGTGCGGCTGGATCACCTCCTTTCTAAGGAG>M._malmoenseT------TTTTGTTTGGAGAGTTTGATCCTGGCTCAGGACGAACGCTGGCGGCGTGCTTAACACATGCAAGTCGAACGGAAAGGTCTCT------------T--C---------------GGAGATACTCGAGTGGCGAACGGGTGAGTAACACGTGGGTAATCTGCCCTGCACTTC-GGGATAAGCCTGGGAAACTGGGTCTAATACCGAATAGGACCC-C-GAGGCGCATGCCT-T-GGGGTGGAAAG--CTT--T----T----GCGGTGTGGGATGGGCCCGCGGCCTATCAGCTTGTTGGTGGGGTGACGGCCTACCAAGGCGACGACGGGTAGCCGGCCTGAGAGGGTGTCCGGCCACACTGGGACTGAGATACGGCCCAGACTCCTACGGGAGGCAGCAGTGGGGAATATTGCACAATGGGCGCAAGCCTGATGCAGCGACGCCGCGTGGGGGATGACGGCCTTCGGGTTGTAAACCTCTTTCACCATCGACGAAGGTCCGG-GTTT--TCTC-GGATTGACGGTAGGTGGAGAAGAAGCACCGGCCAACTACGTGCCAGCAGCCGCGGTAATACGTAGGGTGCGAGCGTTGTCCGGAATTACTGGGCGTAAAGAGCTCGTAGGTGGTTTGTCGCGTTGTTCGTGAAATCTC-ACGGCTTAACTGTGAGCGTGCGGGCGATACGGGCAGACTGGAGTACTGCAGGGGAGACTGGAATTCCTGGTGTAGCGGTGGAATGCGCAGATATCAGGAGGAACACCGGTGGCGAAGGCGGGTCTCTGGGCAGTAACTGACGCTGAGGAGCGAAAGCGTGGGGAGCGAACAGGATTAGATACCCTGGTAGTCCACGCCGTAAACGGTGGGTACTAGGTGTGGGTTTCCTTCCTT--GGGATCCGTGCCGTAGCTAACGCATTAAGTACCCCGCCTGGGGAGTACGGCCGCAAGGCTAAAACTCAAAGGAATTGACGGGGGCCCGCACAAGCGGCGGAGCATGTGGATTAATTCGATGCAACGCGAAGAACCTTACCTGGGTTTGACATGCACAGGACGCGTCTAGAGATAGGCGTTCCCTTGTGGCCTGTGTGCAGGTGGTGCATGGCTGTCGTCAGCTCGTGTCGTGAGATGTTGGGTTAAGTCCCGCAACGAGCGCAACCCTTGTCTCATGTTGCCAGCGGGTAATGCCGGGGACTCGTGAGAGACTGCCGGGGTCAACTCGGAGGAAGGTGGGGATGACGTCAAGTCATCATGCCCCTTATGTCCAGGGCTTCACACATGCTACAATGGCCGGTACAAAGGGCTGCGATGCCGCAAGGTTAAGCGAATCCTT-TTAAAGCCGGTCTCAGTTCGGATCGGGGTCTGCAACTCGACCCCGTGAAGTCGGAGTCGCTAGTAATCGCAGATCAGCAACGCTGCGGTGAATACGTTCCCGGGCCTTGTACACACCGCCCGTCACGTCATGAAAGTCGGTAACACCCGAAGCCAGTGGCCTAACCCT----CGGGAGGGAGCTGTCGAAGGTGGGATCGGCGATTGGGACGAAGTCGTAACAAGGTAGCCGTACCGGAAGGTGCGGCTGGATCACCTCCTTTCTAAGGAG>M._mantenii---------TTGTTTGGAGAGTTTGATCCTGGCTCAGGACGAACGCTGGCGGCGTGCTTAACACATGCAAGTCGAACGGAAAGGCCTCT------------T--C---------------GGAGGTACTCGAGTGGCGAACGGGTGAGTAACACGTGGGCAATCTGCCCTGCACTTC-GGGATAAGCCTGGGAAACTGGGTCTAATACCGAATAGGACCA-C-TTGGCGCATGCCT-T-GTGGTGGAAAG--CTT--T----T----GCGGTGTGGGATGGGCCCGCGGCCTATCAGCTTGTTGGTGGGGTGATGGCCTACCAAGGCGACGACGGGTAGCCGGCCTGAGAGGGTGTCCGGCCACACTGGGACTGAGATACGGCCCAGACTCCTACGGGAGGCAGCAGTGGGGAATATTGCACAATGGGCGCAAGCCTGATGCAGCGACGCCGCGTGGGGGATGACGGCCTTCGGGTTGTAAACCTCTTTCACCATCGACGAAGGCTCAC-TTT-----GT-GGGTTGACGGTAGGTGGAGAAGAAGCACCGGCCAACTACGTGCCAGCAGCCGCGGTAATACGTAGGGTGCGAGCGTTGTCCGGAATTACTGGGCGTAAAGAGCTCGTAGGTGGTTTGTCGCGTTGTTCGTGAAATCTC-ACGGCTTAACTGTGAGCGTGCGGGCGATACGGGCAGACTAGAGTACTGCAGGGGAGACTGGAATTCCTGGTGTAGCGGTGGAATGCGCAGATATCAGGAGGAACACCGGTGGCGAAGGCGGGTCTCTGGGCAGTAACTGACGCTGAGGAGCGAAAGCGTGGGGAGCGAACAGGATTAGATACCCTGGTAGTCCACGCCGTAAACGGTGGGTACTAGGTGTGGGTTTCCTTCCTT--GGGATCCGTGCCGTAGCTAACGCATTAAGTACCCCGCCTGGGGAGTACGGCCGCAAGGCTAAAACTCAAAGGAATTGACGGGGGCCCGCACAAGCGGCGGAGCATGTGGATTAATTCGATGCAACGCGAAGAACCTTACCTGGGTTTGACATGCACAGGACGCGTCTAGAGATAGGCGTTCCCTTGTGGCCTGTGTGCAGGTGGTGCATGGCTGTCGTCAGCTCGTGTCGTGAGATGTTGGGTTAAGTCCCGCAACGAGCGCAACCCTTGTCTCATGTTGCCAGCGGGTAATGCCGGGGACTCGTGAGAGACTGCCGGGGTCAACTCGGAGGAAGGTGGGGATGACGTCAAGTCATCATGCCCCTTATGTCCAGGGCTTCACACATGCTACAATGGCCGGTACAAAGGGCTGCGATGCCGTAAGGTTAAGCGAATCCTT-TTAAAGCCGGTCTCAGTTCGGATTGGGGTCTGCAACTCGACCCCATGAAGTCGGAGTCGCTAGTAATCGCAGATCAGCAACGCTGCGGTGAATACGTTCCCGGGCCTTGTACACACCGCCCGTCACGTCATGAAAGTCGGTAACACCCGAAGCCAGTGGCCTAACCCTT---TGGGAGGGAGCTGTCGAAGGTGGGATCGGCGATTGGGACGAAGTCGTAACAAGGTAGCCGTACCGGAAGGTGCGGCTGGATCACCTCCTTTCTAA---->M._marinum---------TTGTTTGGAGAGTTTGATCCTGGCTCAGGACGAACGCTGGCGGCGTGCTTAACACATGCAAGTCGAACGGAAAGGTCTCT------------T--C---------------GGAGATACTCGAGTGGCGAACGGGTGAGTAACACGTGGGCGATCTGCCCTGCACTTC-GGGATAAGCCTGGGAAACTGGGTCTAATACCGGATAGGACCA-C-GGGATTCATGTCC-T-GTGGTGGAAAG--CTT--T----T----GCGGTGTGGGATGGGCCCGCGGCCTATCAGCTTGTTGGTGGGGTAACGGCCTACCAAGGCGACGACGGGTAGCCGGCCTGAGAGGGTGTCCGGCCACACTGGGACTGAGATACGGCCCAGACTCCTACGGGAGGCAGCAGTGGGGAATATTGCACAATGGGCGCAAGCCTGATGCAGCGACGCCGCGTGGGGGATGACGGCCTTCGGGTTGTAAACCTCTTTCACCATCGACGAAGGTTCGG-GTTT--TCTC-GGATTGACGGTAGGTGGAGAAGAAGCACCGGCCAACTACGTGCCAGCAGCCGCGGTAATACGTAGGGTGCGAGCGTTGTCCGGAATTACTGGGCGTAAAGAGCTCGTAGGTGGTTTGTCGCGTTGTTCGTGAAATCTC-ACGGCTTAACTGTGAGCGTGCGGGCGATACGGGCAGACTAGAGTACTGCAGGGGAGACTGGAATTCCTGGTGTAGCGGTGGAATGCGCAGATATCAGGAGGAACACCGGTGGCGAAGGCGGGTCTCTGGGCAGTAACTGACGCTGAGGAGCGAAAGCGTGGGGAGCGAACAGGATTAGATACCCTGGTAGTCCACGCCGTAAACGGTGGGTACTAGGTGTGGGTTTCCTTCCTT--GGGATCCGTGCCGTAGCTAACGCATTAAGTACCCCGCCTGGGGAGTACGGCCGCAAGGCTAAAACTCAAAGGAATTGACGGGGGCCCGCACAAGCGGCGGAGCATGTGGATTAATTCGATGCAACGCGAAGAACCTTACCTGGGTTTGACATGCACAGGACGCGTCTAGAGATAGGCGTTCCCTTGTGGCCTGTGTGCAGGTGGTGCATGGCTGTCGTCAGCTCGTGTCGTGAGATGTTGGGTTAAGTCCCGCAACGAGCGCAACCCTTGTCTCATGTTGCCAGCACGTAATGGTGGGGACTCGTGAGAGACTGCCGGGGTCAACTCGGAGGAAGGTGGGGATGACGTCAAGTCATCATGCCCCTTATGTCCAGGGCTTCACACATGCTACAATGGCCGGTACAAAGGGCTGCGATGCCGCGAGGTTAAGCGAATCCTT--TAAAGCCGGTCTCAGTTCGGATCGGGGTCTGCAACTCGACCCCGTGAAGTCGGAGTCGCTAGTAATCGCAGATCAGCAACGCTGCGGTGAATACGTTCCCGGGCCTTGTACACACCGCCCGTCACGTCATGAAAGTCGGTAACACCCGAAGCCAGTGGCCTAACCCTT---TGGGAGGGAGCTGTCGAAGGTGGGATCGGCGATTGGGACGAAGTCGTAACAAGGTAGCCGTACCGGAAGGTGCGGCTGGATCACCTCCTTTCTAAGGAG>M._marseillense----------TGTTTGGAGAGTTTGATCCTGGCTCAGGACGAACGCTGGCGGCGTGCTTAACACATGCAAGTCGAACGGAAAGGCCCCT------------T--C---------------GGGGGTACTCGAGTGGCGAACGGGTGAGTAACACGTGGGCAATCTGCCCTGCACTTC-GGGATAAGCCTGGGAAACTGGGTCTAATACCGGATAGGACCT-T-TAGACGCATGTCT-T-TTGGTGGAAAG--CTT--T----T----GCGGTGTGGGATGGGCCCGCGGCCTATCAGCTTGTTGGTGGGGTGATGGCCTACCAAGGCGACGACGGGTAGCCGGCCTGAGAGGGTGTCCGGCCACACTGGGACTGAGATACGGCCCAGACTCCTACGGGAGGCAGCAGTGGGGAATATTGCACAATGGGCGCAAGCCTGATGCAGCGACGCCGCGTGGGGGATGACGGCCTTCGGGTTGTAAACCTCTTTCACCATCGACGAAGGTTCGG-GTTT--TCTC-GGATTGACGGTAGGTGGAGAAGAAGCACCGGCCAACTACGTGCCAGCAGCCGCGGTAATACGTAGGGTGCGAGCGTTGTCCGGAATTACTGGGCGTAAAGAGCTCGTAGGTGGTTTGTCGCGTTGTTCGTGAAATCTC-ACGGCTTAACTGTGAGCGTGCGGGCGATACGGGCAGACTAGAGTACTGCAGGGGAGACTGGAATTCCTGGTGTAGCGGTGGAATGCGCAGATATCAGGAGGAACACCGGTGGCGAAGGCGGGTCTCTGGGCAGTAACTGACGCTGAGGAGCGAAAGCGTGGGGAGCGAACAGGATTAGATACCCTGGTAGTCCACGCCGTAAACGGTGGGTACTAGGTGTGGGTTTCCTTCCTT--GGGATCCGTGCCGTAGCTAACGCATTAAGTACCCCGCCTGGGGAGTACGGCCGCAAGGCTAAAACTCAAAGGAATTGACGGGGGCCCGCACAAGCGGCGGAGCATGTGGATTAATTCGATGCAACGCGAAGAACCTTACCTGGGTTTGACATGCACAGGACGCGTCTAGAGATAGGCGTTCCCTTGTGGCCTGTGTGCAGGTGGTGCATGGCTGTCGTCAGCTCGTGTCGTGAGATGTTGGGTTAAGTCCCGCAACGAGCGCAACCCTTGTCTCATGTTGCCATCGGGTAATGCCGGGGACTCGTGAGAGACTGCCGGGGTCAACTCGGAGGAAGGTGGGGATGACGTCAAGTCATCATGCCCCTTATGTCCAGGGCTTCACACATGCTACAATGGCCGGTACAAAGGGCTGCGATGCCGCAAGGTTAAGCGAATCCTT-TTAAAGCCGGTCTCAGTTCGGATTGGGGTCTGCAACTCGACCCCATGAAGTCGGAGTCGCTAGTAATCGCAGATCAGCAACGCTGCGGTGAATACGTTCCCGGGCCTTGTACACACCGCCCGTCACGTCATGAAAGTCGGTAACACCCGAAGCCAGTGGCCTAACCCT----TGGGAGGGAGCTGTCGAAGGTGGGATCGGCGATTGGGACGAAGTCGTAACAAGGTAGCCGTACCGGAAGGTGCGGCTGGATCACCTCCTTT-------->M._nebraskense----------TGTTTGGAGAGTTTGATCCTGGCTCAGGACGAACGCTGGCGGCGTGCTTAACACATGCAAGTCGAACGGAAAGGTCTCT------------T--C---------------GGAGATACTCGAGTGGCGAACGGGTGAGTAACACGTGGGTAATCTGCCCTGCACTTC-GGGATAAGCCTGGGAAACTGGGTCTAATACCGAATAGGACCA-C-TTGGCGCATGCCT-T-GTGGTGGAAAG--CTT--T----T----GCGGTGTGGGATGGGCCCGCGGCCTATCAGCTTGTTGGTGGGGTGATGGCCTACCAAGGCGACGACGGGTAGCCGGCCTGAGAGGGTGTCCGGCCACACTGGGACTGAGATACGGCCCAGACTCCTACGGGAGGCAGCAGTGGGGAATATTGCACAATGGGCGCAAGCCTGATGCAGCGACGCCGCGTGGGGGATGACGGCCTTCGGGTTGTAAACCTCTTTCACCATCGACGAAGGTCCGG-GTTT--TCTC-GGATTGACGGTAGGTGGAGAAGAAGCACCGGCCAACTACGTGCCAGCAGCCGCGGTAATACGTAGGGTGCGAGCGTTGTCCGGAATTACTGGGCGTAAAGAGCTCGTAGGTGGTTTGTCGCGTTGTTCGTGAAATCTC-ACGGCTTAACTGTGAGCGTGCGGGCGATACGGGCAGACTAGAGTACTGCAGGGGAGACTGGAATTCCTGGTGTAGCGGTGGAATGCGCAGATATCAGGAGGAACACCGGTGGCGAAGGCGGGTCTCTGGGCAGTAACTGACGCTGAGGAGCGAAAGCGTGGGGAGCGAACAGGATTAGATACCCTGGTAGTCCACGCCGTAAACGGTGGGTACTAGGTGTGGGTTTCCTTCCTT--GGGATCCGTGCCGTAGCTAACGCATTAAGTACCCCGCCTGGGGAGTACGGCCGCAAGGCTAAAACTCAAAGGAATTGACGGGGGCCCGCACAAGCGGCGGAGCATGTGGATTAATTCGATGCAACGCGAAGAACCTTACCTGGGTTTGACATGCACAGGACGCGTCTAGAGATAGGCGTTCCCTTGTGGCCTGTGTGCAGGTGGTGCATGGCTGTCGTCAGCTCGTGTCGTGAGATGTTGGGTTAAGTCCCGCAACGAGCGCAACCCCCGTCTCATGTTGCCAGCGGGTAATGCCGGGGACTCGTGAGAGACTGCCGGGGTCAACTCGGAGGAAGGTGGGGATGACGTCAAGTCATCATGCCCCTTATGTCCAGGGCTTCACACATGCTACAATGGCCGGTACAAAGGGCTGCGATGCCGCAAGGTTAAGCGAATCCTT-TTAAAGCCGGTCTCAGTTCGGATCGGGGTCTGCAACTCGACCCCGTGAAGTCGGAGTCGCTAGTAATCGCAGATCAGCAACGCTGCGGTGAATACGTTCCCGGGCCTTGTACACACCGCCCGTCACGTCATGAAAGTCGGTAACACCCGAAGCCAGTGGCCTAACCCT----TGGGAGGGAGCTGTCGAAGGTGGGATCGGCGATTGGGACGAAGTCGTAACAAGGTAGCCGTACCGGAAGGTGCGGCTGGATCACCTCCTTT-------->M._neumannii----------TGTTTGGAGAGTTTGATTCTGGCTCAGGACGAACGCTGGCGGCGTGCTTAACACATGCAAGTCGAACGGAAAGGCCCCT------------T--T---------------GGGGGTGCTCGAGTGGCGAACGGGTGAGTAACACGTGGGTGATCTGCCCTGCACTTT-GGGATAAGCCTGGGAAACTGGGTCTAATACCGAATACACCTTGC-TGGTCGCATGGCCTG-GTGGGGGAAAG--CTT--T----T----GCGGTGTGGGATGGGCCCGCGGCCTATCAGCTTGTTGGTGAGGTTATGGCTTACCAAGGCGACGACGGGTAGCCGGCCTGAGAGGGTGACCGGCCACACTGGGACTGAGATACGGCCCAGACTCCTACGGGAGGCAGCAGTGGGGAATATTGCACAATGGGCGCAAGCCTGATGCAGCGACGCCGCGTGGGGGATGACGGCCTTCGGGTTGTAAACCTCTTTCAGTGCCGACGAAGCGT----------------AAGTGACGGTAGGCACAGAAGAAGGACCGGCCAACTACGTGCCAGCAGCCGCGGTAATACGTAGGGTCCGAGCGTTGTCCGGAATTACTGGGCGTAAAGAGCTCGTAGGTGGTTTGTCGCGTTGTCCGTGAAAACTC-ACAGCTTAACTGTGGGCGTGCGGGCGATACGGGCAGACTTGAGTACTGCAGGGGAGACTGGAATTCCTGGTGTAGCGGTGGAATGCGCAGATATCAGGAGGAACACCGGTGGCGAAGGCGGGTCTCTGGGCAGTAACTGACGCTGAGGAGCGAAAGCGTGGGGAGCGAACAGGATTAGATACCCTGGTAGTCCACGCCGTAAACGGTGGGTACTAGGTGTGGGTTTCCTTCCTT--GGGATCCGTGCCGTAGCTAACGCATTAAGTACCCCGCCTGGGGAGTACGGCCGCAAGGCTAAAACTCAAAGAAATTGACGGGGGCCCGCACAAGCGGCGGAGCATGTGGATTAATTCGATGCAACGCGAAGAACCTTACCTGGGTTTGACATGCACAGGACGACTGCAGAGATGTGGTTTCCCTTGTGGCCTGTGTGCAGGTGGTGCATGGCTGTCGTCAGCTCGTGTCGTGAGATGTTGGGTTAAGTCCCGCAACGAGCGCAACCCTTGTCTCATGTTGCCAGCGCGTTATGGCGGGGACTCGTGAGAGACTGCCGGGGTCAACTCGGAGGAAGGTGGGGATGACGTCAAGTCATCATGCCCCTTATGTCCAGGGCTTCACACATGCTACAATGGCCGGTACAAAGGGCTGCGATGCCGTGAGGTGGAGCGAATCCTT-TCAAAGCCGGTCTCAGTTCGGATCGGGGTCTGCAACTCGACCCCGTGAAGTCGGAGTCGCTAGTAATCGCAGATCAGCAACGCTGCGGTGAATACGTTCCCGGGCCTTGTACACACCGCCCGTCACGTCATGAAAGTCGGTAACACCCGAAGCCGGTGGCCTAACCCCT-TGTGGGAGGGAGCCGTCGAAGGTGGGATCGGCGATTGGGACGAAGTCGTAACAAGGTAGCCGTACCGGAAGGTGCGGCTGGATCACCTCCTTT-------->M._noviomagense---------TTGTTTGGAGAGTTTGATCCTGGCTCAGGACGAACGCTGGCGGCGTGCTTAACACATGCAAGTCGAACGGAAAGGCCCCT------------TT-CG--------------GGGGGTGCTCGAGTGGCGAACGGGTGAGTAACACGTGGGTGATCTGCCCTGCACTTT-GGGATAAGCCTGGGAAACTGGGTCTAATACCGGATAGGACCA-T-TCCATGCATGTGG-G-GTGGTGGAAAG--CGT--TTTGTA----GCGGTGTGGGATGGGCCCGCGGCCTATCAGCTTGTTGGTGGGGTGATGGCCTACCAAGGCGACGACGGGTAGCCGGCCTGAGAGGGTGTCCGGCCACACTGGGACTGAGATACGGCCCAGACTCCTACGGGAGGCAGCAGTGGGGAATATTGCACAATGGGCGCAAGCCTGATGCAGCGACGCCGCGTGGGGGATGACGGCCTTCGGGTTGTAAACCTCTTTCACCATCGACGAAGCCGCAC-GTTT--TCGTGTTGGTGACGGTAGGTGGAGAAGAAGCACCGGCCAACTACGTGCCAGCAGCCGCGGTAATACGTAGGGTGCGAGCGTTGTCCGGAATTACTGGGCGTAAAGAGCTCGTAGGTGGTTTGTCGCGTTGTTCGTGGAAGTCC-ACAGCTTAACTGTGGGGGTGCGGGCGATACGGGCAGACTGGAGTACTGCAGGGGAGACTGGAATTCCTGGTGTAGCGGTGGAATGCGCAGATATCAGGAGGAACACCGGTGGCGAAGGCGGGTCTCTGGGCAGTAACTGACGCTGAGGAGCGAAAGCGTGGGGAGCGAACAGGATTAGATACCCTGGTAGTCCACGCCGTAAACGGTGGGTACTAGGTGTGGGTTTCCTTCCTT--GGGATCCGTGCCGTAGCTAACGCATTAAGTACCCCGCCTGGGGAGTACGGCCGCAAGGCTAAAACTCAAAGGAATTGACGGGGGCCCGCACAAGCGGCGGAGCATGTGGATTAATTCGATGCAACGCGAAGAACCTTACCTGGGTTTGACATGCACAGGACGCGTCTAGAGATAGGCGTTCCCTTGTGGCCTGTGTGCAGGTGGTGCATGGCTGTCGTCAGCTCGTGTCGTGAGATGTTGGGTTAAGTCCCGCAACGAGCGCAACCCTTGTCCCATGTTGCCAGCACGTGATGGTGGGGACTCATGGGAGACTGCCGGGGTCAACTCGGAGGAAGGTGGGGATGACGTCAAGTCATCATGCCCCTTATGTCCAGGGCTTCACACATGCTACAATGGCCGGTACAAAGGGCTGCGATGCCGTGAGGTTAAGCGAATCCTCGCTAAAGCCGGTCTCAGTTCGGATCGGGGTCTGCAACTCGACCCCGTGAAGTCGGAGTCGCTAGTAATCGCAGATCAGCAATGCTGCGGTGAATACGTTCCCGGGCCTTGTACACACCGCCCGTCACGTCATGAAAGTCGGTAACACCCGAAGCCGCTGGCCTAACCCGT--ATGGGAGGGAGGCGTCGAAGGTGGGATCGGCGATTGGGACGAAGTCGTAACAAGGTAGCCGTACCGGAAGGTGCGGCTGGATCACCTCCTTTCTAA---->M._palustre----------TGTTTGGAGAGTTTGATCCTGGCTCAGGACGAACGCTGGCGGCGTGCTTAACACATGCAAGTCGAACGGAAAGGCCCCT------------T--C---------------GGGGGTACTCGAGTGGCGAACGGGTGAGTAACACGTGGGTAATCTGCCCTGCACTTC-GGGATAAGCCTGGGAAACTGGGTCTAATACCGGATAGGACCT-T-TAGGCGCATGCCT-T-TTGGTGGAAAG--CTT--T----T----GCGGTGTGGGATGGGCCCGCGGCCTATCAGCTTGTTGGTGGGGTGACGGCCTACCAAGGCGACGACGGGTAGCCGGCCTGAGAGGGTGTCCGGCCACACTGGGACTGAGATACGGCCCAGACTCCTACGGGAGGCAGCAGTGGGGAATATTGCACAATGGGCGCAAGCCTGATGCAGCGACGCCGCGTGGGGGATGACGGCCTTCGGGTTGTAAACCTCTTTCAGCAGGGACGAAGCGC----------------AAGTGACGGTACCTGCAGAAGAAGCACCGGCCAACTACGTGCCAGCAGCCGCGGTAATACGTAGGGTGCGAGCGTTGTCCGGAATTACTGGGCGTAAAGAGCTCGTAGGTGGTTTGTCGCGTTGTTCGTGAAAACCGGGGGGCTTAACCCTCGGCGTGCGGGCGATACGGGCAGACTGGAGTACTGCAGGGGAGACTGGAATTCCTGGTGTAGCGGTGGAATGCGCAGATATCAGGAGGAACACCGGTGGCGAAGGCGGGTCTCTGGGCAGTAACTGACGCTGAGGAGCGAAAGCGTGGGGAGCGAACAGGATTAGATACCCTGGTAGTCCACGCCGTAAACGGTGGGTACTAGGTGTGGGTTTCCTTCCTT--GGGATCCGTGCCGTAGCTAACGCATTAAGTACCCCGCCTGGGGAGTACGGCCGCAAGGCTAAAACTCAAAGGAATTGACGGGGGCCCGCACAAGCGGCGGAGCATGTGGATTAATTCGATGCAACGCGAAGAACCTTACCTGGGTTTGACATGCACAGGACGCGTCTAGAGATAGGCGTTCCCTTGTGGCCTGTGTGCAGGTGGTGCATGGCTGTCGTCAGCTCGTGTCGTGAGATGTTGGGTTAAGTCCCGCAACGAGCGCAACCCTTGTCTCATGTTGCCAGCGGGTAATGCCGGGGACTCGTGAGAGACTGCCGGGGTCAACTCGGAGGAAGGTGGGGATGACGTCAAGTCATCATGCCCCTTATGTCCAGGGCTTCACACATGCTACAATGGCCGGTACAAAGGGCTGCGATGCCGCAAGGTTAAGCGAATCCTT--TAAAGCCGGTCTCAGTTCGGATCGGGGTCTGCAACTCGACCCCGTGAAGTCGGAGTCGCTAGTAATCGCAGATCAGCAACGCTGCGGTGAATACGTTCCCGGGCCTTGTACACACCGCCCGTCACGTCATGAAAGTCGGTAACACCCGAAGCCAGTGGCCTAACCCTT---TGGGAGGGAGCTGTCGAAGGTGGGATCGGCGATTGGGACGAAGTCGTAACAAGGTAGCCGTACCGGAAGGTGCGGCTGGATCACCTCCTTT-------->M._paragordonae----------TGTTTGGAGAGTTTGATTCTGGCTCAGGACGAACGCTGGCGGCGTGCTTAACACATGCAAGTCGAACGGTAAGGCCC-T------------T--C----------------GGGGTACACGAGTGGCGAACGGGTGAGTAACACGTGGGTAATCTGCCCTGCACATC-GGGATAAGCCTGGGAAACTGGGTCTAATACCGAATAGGACCA-C-AGAACACATGTCC-T-GTGGTGGAAAG--CTT--T----T----GCGGTGTGGGATGGGCCCGCGGCCTATCAGCTTGTTGGTGGGGTGATGGCCCACCAAGGCGACGACGGGTAGCCGGCCTGAGAGGGTGTCCGGCCACACTGGGACTGAGATACGGCCCAGACTCCTACGGGAGGCAGCAGTGGGGAATATTGCACAATGGGCGAAAGCCTGATGCAGCGACGCCGCGTGGGGGATGACGGCCTTCGGGTTGTAAACCTCTTTCACCATCGACGAAGGTTCGG-GTTT--TCTC-GGATTGACGGTAGGTGGAGAAGAAGCACCGGCCAACTACGTGCCAGCAGCCGCGGTAATACGTAGGGTGCGAGCGTTGTCCGGAATTACTGGGCGTAAAGAGCTCGTAGGTGGTTTGTCGCGTTGTTCGTGAAATCTC-ACGGCTTAACTGTGAGCGTGCGGGCGATACGGGCAGACTTGAGTACTGCAGGGGAGACTGGAATTCCTGGTGTAGCGGTGGAATGCGCAGATATCAGGAGGAACACCGGTGGCGAAGGCGGGTCTCTGGGCAGTAACTGACGCTGAGGAGCGAAAGCGTGGGGAGCGAACAGGATTAGATACCCTGGTAGTCCACGCCGTAAACGGTGGGTACTAGGTGTGGGTTTCCTTCCTT--GGGATCCGTGCCGTAGCTAACGCATTAAGTACCCCGCCTGGGGAGTACGGCCGCAAGGCTAAAACTCAAAGAAATTGACGGGGGCCCGCACAAGCGGCGGAGCATGTGGATTAATTCGATGCAACGCGAAGAACCTTACCTGGGTTTGACATGCACAGGACGCCGGCAGAGATGTCGGTTCCCTTGTGGCCTGTGTGCAGGTGGTGCATGGCTGTCGTCAGCTCGTGTCGTGAGATGTTGGGTTAAGTCCCGCAACGAGCGCAACCCTTGTCTCATGTTGCCAGCGGGTAATGCCGGGGACTCGTGAGAGACTGCCGGGGTCAACTCGGAGGAAGGTGGGGATGACGTCAAGTCATCATGCCCCTTATGTCCAGGGCTTCACACATGCTACAATGGCCGGTACAAAGGGCTGCGATGCCGCGAGGTTAAGCGAATCCTT-TTAAAGCCGGTCTCAGTTCGGATCGGGGTCTGCAACTCGACCCCGTGAAGTCGGAGTCGCTAGTAATCGCAGATCAGCAACGCTGCGGTGAATACGTTCCCGGGCCTTGTACACACCGCCCGTCACGTCATGAAAGTCGGTAACACCCGAAGCCAGTGGCCTAACCCTT---TGGGAGGGAGCTGTCGAAGGTGGGATCGGCGATTGGGACGAAGTCGTAACAAGGTAGCCGTACCGGAAGGTGCGGCTGGATCACCTCCTTT-------->M._paraintracellulare----------TGTTTGGAGAGTTTGATCCTGGCTCAGGACGAACGCTGGCGGCGTGCTTAACACATGCAAGTCGAACGGAAAGGCCCCT------------T--C---------------GGGGGTACTCGAGTGGCGAACGGGTGAGTAACACGTGGGCAATCTGCCCTGCACTTC-GGGATAAGCCTGGGAAACTGGGTCTAATACCGGATAGGACCT-T-TAGGCGCATGTCT-T-TAGGTGGAAAG--CTT--T----T----GCGGTGTGGGATGGGCCCGCGGCCTATCAGCTTGTTGGTGGGGTGATGGCCTACCAAGGCGACGACGGGTAGCCGGCCTGAGAGGGTGTCCGGCCACACTGGGACTGAGATACGGCCCAGACTCCTACGGGAGGCAGCAGTGGGGAATATTGCACAATGGGCGCAAGCCTGATGCAGCGACGCCGCGTGGGGGATGACGGCCTTCGGGTTGTAAACCTCTTTCACCATCGACGAAGGTCCGG-GTTT--TCTC-GGATTGACGGTAGGTGGAGAAGAAGCACCGGCCAACTACGTGCCAGCAGCCGCGGTAATACGTAGGGTGCGAGCGTTGTCCGGAATTACTGGGCGTAAAGAGCTCGTAGGTGGTTTGTCGCGTTGTTCGTGAAATCTC-ACGGCTTAACTGTGAGCGTGCGGGCGATACGGGCAGACTAGAGTACTGCAGGGGAGACTGGAATTCCTGGTGTAGCGGTGGAATGCGCAGATATCAGGAGGAACACCGGTGGCGAAGGCGGGTCTCTGGGCAGTAACTGACGCTGAGGAGCGAAAGCGTGGGGAGCGAACAGGATTAGATACCCTGGTAGTCCACGCCGTAAACGGTGGGTACTAGGTGTGGGTTTCCTTCCTT--GGGATCCGTGCCGTAGCTAACGCATTAAGTACCCCGCCTGGGGAGTACGGCCGCAAGGCTAAAACTCAAAGGAATTGACGGGGGCCCGCACAAGCGGCGGAGCATGTGGATTAATTCGATGCAACGCGAAGAACCTTACCTGGGTTTGACATGCACAGGACGCGTCTAGAGATAGGCGTTCCCTTGTGGCCTGTGTGCAGGTGGTGCATGGCTGTCGTCAGCTCGTGTCGTGAGATGTTGGGTTAAGTCCCGCAACGAGCGCAACCCTTGTCTCATGTTGCCAGCGGGTAATGCCGGGGACTCGTGAGAGACTGCCGGGGTCAACTCGGAGGAAGGTGGGGATGACGTCAAGTCATCATGCCCCTTATGTCCAGGGCTTCACACATGCTACAATGGCCGGTACAAAGGGCTGCGATGCCGCAAGGTTAAGCGAATCCTT-TTAAAGCCGGTCTCAGTTCGGATTGGGGTCTGCAACTCGACCCCATGAAGTCGGAGTCGCTAGTAATCGCAGATCAGCAACGCTGCGGTGAATACGTTCCCGGGCCTTGTACACACCGCCCGTCACGTCATGAAAGTCGGTAACACCCGAAGCCAGTGGCCTAACCCT----TGGGAGGGAGCTGTCGAAGGTGGGATCGGCGATTGGGACGAAGTCGTAACAAGGTAGCCGTACCGGAAGGTGCGGCTGGATCACCTCCTTT-------->M._parascrofulaceum----------TGTTTGGAGAGTTTGATCCTGGCTCAGGACGAACGCTGGCGGCGTGCTTAACACATGCAAGTCGAACGGAAAGGTCTCT------------T--C---------------GGAGATACTCGAGTGGCGAACGGGTGAGTAACACGTGGGCAATCTGCCCTGCACTTC-GGGATAAGCCTGGGAAACTGGGTCTAATACCGGATAGGACCA-C-TTGGCGCATGCCT-T-GTGGTGGAAAG--CTT--T----T----GCGGTGTGGGATGGGCCCGCGGCCTATCAGCTAGTTGGTGGGGTGATGGCCTACCAAGGCGACGACGGGTAGCCGGCCTGAGAGGGTGTCCGGCCACACTGGGACTGAGATACGGCCCAGACTCCTACGGGAGGCAGCAGTGGGGAATATTGCACAATGGGCGCAAGCCTGATGCAGCGACGCCGCGTGGGGGATGACGGCCTTCGGGTTGTAAACCTCTTTCAGCAGGGACGAAGCGC----------------AAGTGACGGTACCTGCAGAAGAAGCACCGGCCAACTACGTGCCAGCAGCCGCGGTAATACGTAGGGTGCGAGCGTTGTCCGGAATTACTGGGCGTAAAGAGCTCGTAGGTGGTTTGTCGCGTTGTTCGTGAAAACCG-GGGGCTTAACCCTCGGCGTGCGGGCGATACGGGCAGACTAGAGTACTGCAGGGGAGACTGGAATTCCTGGTGTAGCGGTGGAATGCGCAGATATCAGGAGGAACACCGGTGGCGAAGGCGGGTCTCTGGGCAGTAACTGACGCTGAGGAGCGAAAGCGTGGGGAGCGAACAGGATTAGATACCCTGGTAGTCCACGCCGTAAACGGTGGGTACTAGGTGTGGGTTTCCTTCCTT--GGGATCCGTGCCGTAGCTAACGCATTAAGTACCCCGCCTGGGGAGTACGGCCGCAAGGCTAAAACTCAAAGGAATTGACGGGGGCCCGCACAAGCGGCGGAGCATGTGGATTAATTCGATGCAACGCGAAGAACCTTACCTGGGTTTGACATGCACAGGACGCGTCTAGAGATAGGCGTTCCCTTGTGGCCTGTGTGCAGGTGGTGCATGGCTGTCGTCAGCTCGTGTCGTGAGATGTTGGGTTAAGTCCCGCAACGAGCGCAACCCTTGTCTCATGTTGCCAGCGGGTAATGCCGGGGACTCGTGAGAGACTGCCGGGGTCAACTCGGAGGAAGGTGGGGATGACGTCAAGTCATCATGCCCCTTATGTCCAGGGCTTCACACATGCTACAATGGCCGGTACAAAGGGCTGCGATGCCGCAAGGTTAAGCGAATCCTT-TTAAAGCCGGTCTCAGTTCGGATCGGGGTCTGCAACTCGACCCCGTGAAGTCGGAGTCGCTAGTAATCGCAGATCAGCAACGCTGCGGTGAATACGTTCCCGGGCCTTGTACACACCGCCCGTCACGTCATGAAAGTCGGTAACACCCGAAGCCAGTGGCCTAACCCT----TGGGAGGGAGCTGTCGAAGGTGGGATCGGCGATTGGGACGAAGTCGTAACAAGGTAGCCGTACCGGAAGGTGCGGCTGGATCACCTCCTTT-------->M._paraseoulense---------TTGTTTGGAGAGTTTGATCCTGGCTCAGGACGAACGCTGGCGGCGTGCTTAACACATGCAAGTCGAACGGAAAGGCCCCT------------T--C---------------GGGGGTACTCGAGTGGCGAACGGGTGAGTAACACGTGGGCAATCTGCCCTGCACTTC-GGGATAAGCCTGGGAAACTGGGTCTAATACCGAATAGGACCA-C-TTGGCGCATGCCT-T-GTGGTGGAAAG--CTT--T----T----GCGGTGTGGGATGGGCCCGCGGCCTATCAGCTTGTTGGTGGGGTGATGGCCTACCAAGGCGACGACGGGTAGCCGGCCTGAGAGGGTGTCCGGCCACACTGGGACTGAGATACGGCCCAGACTCCTACGGGAGGCAGCAGTGGGGAATATTGCACAATGGGCGCAAGCCTGATGCAGCGACGCCGCGTGGGGGATGACGGCCTTCGGGTTGTAAACCTCTTTCACCATCGACGAAGGTCCGG-GT-T--TCTC-GGATTGACGGTAGGTGGAGAAGAAGCACCGGCCAACTACGTGCCAGCAGCCGCGGTAATACGTAGGGTGCGAGCGTTGTCCGGAATTACTGGGCGTAAAGAGCTCGTAGGTGGTTTGTCGCGTTGTTCGTGAAATCTC-ACGGCTTAACTGTGAGCGTGCGGGCGATACGGGCAGACTAGAGTACTGCAGGGGAGACTGGAATTCCTGGTGTAGCGGTGGAATGCGCAGATATCAGGAGGAACACCGGTGGCGAAGGCGGGTCTCTGGGCAGTAACTGACGCTGAGGAGCGAAAGCGTGGGGAGCGAACAGGATTAGATACCCTGGTAGTCCACGCCGTAAACGGTGGGTACTAGGTGTGGGTTTCCTTCCTT--GGGATCCGTGCCGTAGCTAACGCATTAAGTACCCCGCCTGGGGAGTACGGCCGCAAGGCTAAAACTCAAAGGAATTGACGGGGGCCCGCACAAGCGGCGGAGCATGTGGATTAATTCGATGCAACGCGAAGAACCTTACCTGGGTTTGACATGCACAGGACGCGTCTAGAGATAGGCGTTCCCTTGTGGCCTGTGTGCAGGTGGTGCATGGCTGTCGTCAGCTCGTGTCGTGAGATGTTGGGTTAAGTCCCGCAACGAGCGCAACCCTTGTCTCATGTTGCCAGCGGGTAATGCCGGGGACTCGTGAGAGACTGCCGGGGTCAACTCGGAGGAAGGTGGGGATGACGTCAAGTCATCATGCCCCTTATGTCCAGGGCTTCACACATGCTACAATGGCCGGTACAAAGGGCTGCGATGCCGCAAGGTTAAGCGAATCCTT-TTAAAGCCGGTCTCAGTTCGGATCGGGGTCTGCAACTCGACCCCGTGAAGTCGGAGTCGCTAGTAATCGCAGATCAGCAACGCTGCGGTGAATACGTTCCCGGGCCTTGTACACACCGCCCGTCACGTCATGAAAGTCGGTAACACCCGAAGCCAGTGGCCTAACCCT----TGGGAGGGAGCTGTCGAAGGTGGGATCGGCGATTGGGACGAAGTCGTAACAAGGTAGCCGTACCGGAAGGTGCGGCTGGATCACCTCCTTTCTAAGGAG>M._parmense----------TGTTTGGAGAGTTTGATCCTGGCTCAGGACGAACGCTGGCGGCGTGCTTAACACATGCAAGTCGAACGGAAAGGTCTCT------------T--C---------------GGAGATACTCGAGTGGCGAACGGGTGAGTAACACGTGGGCAATCTGCCCTGCACTTC-GGGATAAGCCTGGGAAACTGGGTCTAATACCGGATAGGACCA-C-TTGACGCATGTCT-T-GTGGTGGAAAG--CTT-TT----T----GCGGTGTGGGATGGGCCCGCGGCCTATCAGCTTGTTGGTGGGGTGACGGCCTACCAAGGCGACGACGGGTAGCCGGCCTGAGAGGGTGTCCGGCCACACTGGGACTGAGATACGGCCCAGACTCCTACGGGAGGCAGCAGTGGGGAATATTGCACAATGGGCGCAAGCCTGATGCAGCGACGCCGCGTGGGGGATGACGGCCTTCGGGTTGTAAACCTCTTTCAGCAGGGACGAAGCGC----------------AAGTGACGGTACCTGCAGAAGAAGCACCGGCCAACTACGTGCCAGCAGCCGCGGTAATACGTAGGGTGCGAGCGTTGTCCGGAATTACTGGGCGTAAAGAGCTCGTAGGTGGTTTGTCGCGTTGTTCGTGAAAACCG-GGGGCTTAACCCTCGGCGTGCGGGCGATACGGGCAGACTGGAGTACTGCAGGGGAGACTGGAATTCCTGGTGTAGCGGTGGAATGCGCAGATATCAGGAGGAACACCGGTGGCGAAGGCGGGTCTCTGGGCAGTAACTGACGCTGAGGAGCGAAAGCGTGGGGAGCGAACAGGATTAGATACCCTGGTAGTCCACGCCGTAAACGGTGGGTACTAGGTGTGGGTTTCCTTCCTT--GGGATCCGTGCCGTAGCTAACGCATTAAGTACCCCGCCTGGGGAGTACGGCCGCAAGGCTAAAACTCAAAGGAATTGACGGGGGCCCGCACAAGCGGCGGAGCATGTGGATTAATTCGATGCAACGCGAAGAACCTTACCTGGGTTTGACATGCACAGGACGCCGGCAGAGATGTCGGTTCCCTTGTGGCCTGTGTGCAGGTGGTGCATGGCTGTCGTCAGCTCGTGTCGTGAGATGTTGGGTTAAGTCCCGCAACGAGCGCAACCCTTGTCTCATGTTGCCAGCGGGTAATGCCGGGGACTCGTGAGAGACTGCCGGGGTCAACTCGGAGGAAGGTGGGGATGACGTCAAGTCATCATGCCCCTTATGTCCAGGGCTTCACACATGCTACAATGGCCGGTACAAAGGGCTGCGATGCCGCAAGGTTAAGCGAATCCTT-TTAAAGCCGGTCTCAGTTCGGATCGGGGTCTGCAACTCGACCCCGTGAAGTCGGAGTCGCTAGTAATCGCAGATCAGCAACGCTGCGGTGAATACGTTCCCGGGCCTTGTACACACCGCCCGTCACGTCATGAAAGTCGGTAACACCCGAAGCCAGTGGCCTAACCTT----TTGGAGGGAGCTGTCGAAGGTGGGATCGGCGATTGGGACGAAGTCGTAACAAGGTAGCCGTACCGGAAGGTGCGGCTGGATCACCTCCTTT-------->M._persicum----------TGTTTGGAGAGTTTGATCCTGGCTCAGGACGAACGCTGGCGGCGTGCTTAACACATGCAAGTCGAACGGAAAGGCCC-T------------T--C----------------GGGGTACTCGAGTGGCGAACGGGTGAGTAACACGTGGGCAATCTGCCCTGCACACC-GGGATAAGCCTGGGAAACTGGGTCTAATACCGGATAGGACCA-C-TTGGCGCATGCCT-T-GTGGTGGAAAG--CTT--T----T----GCGGTGTGGGATGGGCCCGCGGCCTATCAGCTTGTTGGTGGGGTGACGGCCTACCAAGGCGACGACGGGTAGCCGGCCTGAGAGGGTGTCCGGCCACACTGGGACTGAGATACGGCCCAGACTCCTACGGGAGGCAGCAGTGGGGAATATTGCACAATGGGCGCAAGCCTGATGCAGCGACGCCGCGTGGGGGATGACGGCCTTCGGGTTGTAAACCTCTTTCACCATCGACGAAGGTCCGG-GTTG--TCTC-GGATTGACGGTAGGTGGAGAAGAAGCACCGGCCAACTACGTGCCAGCAGCCGCGGTAATACGTAGGGTGCGAGCGTTGTCCGGAATTACTGGGCGTAAAGAGCTCGTAGGTGGTTTGTCGCGTTGTTCGTGAAATCTC-ACGGCTTAACTGTGAGCGTGCGGGCGATACGGGCAGACTAGAGTACTGCAGGGGAGACTGGAATTCCTGGTGTAGCGGTGGAATGCGCAGATATCAGGAGGAACACCGGTGGCGAAGGCGGGTCTCTGGGCAGTAACTGACGCTGAGGAGCGAAAGCGTGGGGAGCGAACAGGATTAGATACCCTGGTAGTCCACGCCGTAAACGGTGGGTACTAGGTGTGGGTTTCCTTCCTT--GGGATCCGTGCCGTAGCTAACGCATTAAGTACCCCGCCTGGGGAGTACGGCCGCAAGGCTAAAACTCAAAGGAATTGACGGGGGCCCGCACAAGCGGCGGAGCATGTGGATTAATTCGATGCAACGCGAAGAACCTTACCTGGGTTTGACATGCACAGGACGCGTCTAGAGATAGGCGTTCCCTTGTGGCCTGTGTGCAGGTGGTGCATGGCTGTCGTCAGCTCGTGTCGTGAGATGTTGGGTTAAGTCCCGCAACGAGCGCAACCCTTGTCTCATGTTGCCAGCGGGTAATGCCGGGGACTCGTGAGAGACTGCCGGGGTCAACTCGGAGGAAGGTGGGGATGACGTCAAGTCATCATGCCCCTTATGTCCAGGGCTTCACACATGCTACAATGGCCGGTACAAAGGGCTGCGATGCCGCGAGGTTAAGCGAATCCTT-TTAAAGCCGGTCTCAGTTCGGATCGGGGTCTGCAACTCGACCCCGTGAAGTCGGAGTCGCTAGTAATCGCAGATCAGCAACGCTGCGGTGAATACGTTCCCGGGCCTTGTACACACCGCCCGTCACGTCATGAAAGTCGGTAACACCCGAAGCCAGTGGCCTAACCCT----CGGGAGGGAGCTGTCGAAGGTGGGATCGGCGATTGGGACGAAGTCGTAACAAGGTAGCCGTACCGGAAGGTGCGGCTGGATCACCTCCTTT-------->M._pseudoshottsii---------TTGTTTGGAGAGTTTGATCCTGGCTCAGGACGAACGCTGGCGGCGTGCTTAACACATGCAAGTCGAACGGAAAGGTCTCT------------T--C---------------GGAGACACTCGAGTGGCGAACGGGTGAGTAACACGTGGGCGATCTGCCCTGCACTTC-GGGATAAGCCTGGGAAACTGGGTCTAATACCGGATAGGACCA-C-GGGATTCATGTCC-T-GTGGTGGAAAG--CTT--T----T----GCGGTGTGGGATGGGCCCGCGGCCTATCAGCTTGTTGGTGGGGTAACGGCCTACCAAGGCGACGACGGGTAGCCGGCCTGAGAGGGTGTCCGGCCACACTGGGACTGAGATACGGCCCAGACTCCTACGGGAGGCAGCAGTGGGGAATATTGCACAATGGGCGCAAGCCTGATGCAGCGACGCCGCGTGGGGGATGACGGCCTTCGGGTTGTAAACCTCTTTCACCATCGACGAAGGTTCGG-GTTT--TCTC-GGATTGACGGTAGATGGAGAAGAAGCACCGGCCAACTACGTGCCAGCAGCCGCGGTAATACGTAGGGTGCGAGCGTTGTCCGGAATTACTGGGCGTAAAGAGCTCGTAGGTGGTTTGTCGCGTTGTTCGTGAAATCTC-ACGGCTTAACTGTGAGCGTGCGGGCGATACGGGCAGACTAGAGTACTGCAGGGGAGACTGGAATTCCTGGTGTAGCGGTGGAATGCGCAGATATCAGGAGGAACACCGGTGGCGAAGGCGGGTCTCTGGGCAGTAACTGACGCTGAGGAGCGAAAGCGTGGGGAGCGAACAGGATTAGATACCCTGGTAGTCCACGCCGTAAACGGTGGGTACTAGGTGTGGGTTTCCTTCCTT--GGGATCCGTGCCGTAGCTAACGCATTAAGTACCCCGCCTGGGGAGTACGGCCGCAAGGCTAAAACTCAAAGGAATTGACGGGGGCCCGCACAAGCGGCGGAGCATGTGGATTAATTCGATGCGACGCGAAGAACCTTACCTGGGTTTGACATGCACAGGACTCGTCTAGAGATAGGCGTTCCCTTGTGGCCTGTGTGCAGGTGGTGCATGGCTGTCGTCAGCTCGTGTCGTGAGATGTTGGGTTAAGTCCCGCAACGAGCGCAACCCTTGTCTCATGTTGCCAGCACGTAATGGTGGGGACTCGTGAGAGACTGCCGGGGTCAACTCGGAGGAAGGTGGGGATGACGTCAAGTCATCATGCCCCTTATGCCCAGGGCTTCACACATGCTACAATGGCCGGTACAAAGGGCTGCGATGCCGCGAGGTTAAGCGAATCCTT--TAAAGCCGGTCTCAGTTCGGATCGGGGTCTGCAACTCGACCCCGTGAAGTCGGAGTCGCTAGTAATCGCAGATCAGCAACGCTGCGGTGAATACGTTCCCGGGCCTTGTACACACCGCCCGTCACGTCATGAAAGTCGGTAACACCCGAAGCCAGTGGCCTAACCCTT---TGGGAGGGAGCTGTCGAAGGTGGGATCGGCGATTGGGACGAAGTCGTAACAAGGTAGCCGTACCGGAAGGTGCGGCTGGATCACCTCCTTTCTAAGGAG>M._riyadhense----------TGTTTGGAGAGTTTGATCCTGGCTCAGGACGAACGCTGGCGGCGTGCTTAACACATGCAAGTCGAACGGAAAGGCCCCT------------T--C---------------GGGGGTACTCGAGTGGCGAACGGGTGAGTAACACGTGGGTAATCTGCCCTGCACTTC-GGGATAAGCCTGGGAAACTGGGTCTAATACCGGATAGGACCT-A-GAGGCGCATGCCT-T-GTGGTGGAAAG--CTT--T----T----GCGGTGTGGGATGGGCCCGCGGCCTATCAGCTTGTTGGTGGGGTGACGGCCTACCAAGGCGACGACGGGTAGCCGGCCTGAGAGGGTGTCCGGCCACACTGGGACTGAGATACGGCCCAGACTCCTACGGGAGGCAGCAGTGGGGAATATTGCACAATGGGCGCAAGCCTGATGCAGCGACGCCGCGTGGGGGATGACGGCCTTCGGGTTGTAAACCTCTTTCACCATCGACGAAGGTTCGG-GTTT--TCTC-GGATTGACGGTAGGTGGAGAAGAAGCACCGGCCAACTACGTGCCAGCAGCCGCGGTAATACGTAGGGTGCGAGCGTTGTCCGGAATTACTGGGCGTAAAGAGCTCGTAGGTGGTTTGTCGCGTTGTTCGTGAAATCTC-ACGGCTTAACTGTGAGCGTGCGGGCGATACGGGCAGACTGGAGTACTGCAGGGGAGACTGGAATTCCTGGTGTAGCGGTGGAATGCGCAGATATCAGGAGGAACACCGGTGGCGAAGGCGGGTCTCTGGGCAGTAACTGACGCTGAGGAGCGAAAGCGTGGGGAGCGAACAGGATTAGATACCCTGGTAGTCCACGCCGTAAACGGTGGGTACTAGGTGTGGGTTTCCTTCCTT--GGGATCCGTGCCGTAGCTAACGCATTAAGTACCCCGCCTGGGGAGTACGGCCGCAAGGCTAAAACTCAAAGGAATTGACGGGGGCCCGCACAAGCGGCGGAGCATGTGGATTAATTCGATGCAACGCGAAGAACCTTACCTGGGTTTGACATGCACAGGACGCGTCTAGAGATAGGCGTTCCCTTGTGGCCTGTGTGCAGGTGGTGCATGGCTGTCGTCAGCTCGTGTCGTGAGATGTTGGGTTAAGTCCCGCAACGAGCGCAACCCTTGTCTCATGTTGCCAGCGGGTAATGCCGGGGACTCGTGAGAGACTGCCGGGGTCAACTCGGAGGAAGGTGGGGATGACGTCAAGTCATCATGCCCCTTATGTCCAGGGCTTCACACATGCTACAATGGCCGGTACAAAGGGCTGCGATGCCGTGAGGTTAAGCGAATCCTT-TTAAAGCCGGTCTCAGTTCGGATCGGGGTCTGCAACTCGACCCCGTGAAGTCGGAGTCGCTAGTAATCGCAGATCAGCAACGCTGCGGTGAATACGTTCCCGGGCCTTGTACACACCGCCCGTCACGTCATGAAAGTCGGTAACACCCGAAGCCAGTGGCCTAACCCT----TGGGAGGGAGCTGTCGAAGGTGGGATCGGCGATTGGGACGAAGTCGTAACAAGGTAGCCGTACCGGAAGGTGCGGCTGGATCACCTCCTTT-------->M._saskatchewanense---------TTGTTTGGAGAGTTTGATCCTGGCTCAGGACGAACGCTGGCGGCGTGCTTAACACATGCAAGTCGAACGGAAAGGTCTCT------------T--C---------------GGAGATACTCGAGTGGCGAACGGGTGAGTAACACGTGGGCAATCTGCCCTGCACTTC-GGGATAAGCCTGGGAAACTGGGTCTAATACCGGATAGGACCT-T-TAGGCGCATGCCT-T-TTGGTGGAAAG--CTT--T----T----GCGGTGTGGGATGGGCCCGCGGCCTATCAGCTTGTTGGTGGGGTGATGGCCTACCAAGGCGACGACGGGTAGCCGGCCTGAGAGGGTGTCCGGCCACACTGGGACTGAGATACGGCCCAGACTCCTACGGGAGGCAGCAGTGGGGAATATTGCACAATGGGCGCAAGCCTGATGCAGCGACGCCGCGTGGGGGATGACGGCCTTCGGGTTGTAAACCTCTTTCAGCAGGGACGAAGCGC----------------AAGTGACGGTACCTGCAGAAGAAGCACCGGCCAACTACGTGCCAGCAGCCGCGGTAATACGTAGGGTGCGAGCGTTGTCCGGAATTACTGGGCGTAAAGAGCTCGTAGGTGGTTTGTCGCGTTGTTCGTGAAATCTC-ACGGCTTAACTGTGAGCGTGCGGGCGATACGGGCAGACTAGAGTACTGCAGGGGAGACTGGAATTCCTGGTGTAGCGGTGGAATGCGCAGATATCAGGAGGAACACCGGTGGCGAAGGCGGGTCTCTGGGCAGTAACTGACGCTGAGGAGCGAAAGCGTGGGGAGCGAACAGGATTAGATACCCTGGTAGTCCACGCCGTAAACGGTGGGTACTAGGTGTGGGTTTCCTTCCTT--GGGATCCGTGCCGTAGCTAACGCATTAAGTACCCCGCCTGGGGAGTACGGCCGCAAGGCTAAAACTCAAAGGAATTGACGGGGGCCCGCACAAGCGGCGGAGCATGTGGATTAATTCGATGCAACGCGAAGAACCTTACCTGGGTTTGACATGCACAGGACGCCGGCAGAGATGTCGGTTCCCTTGTGGCCTGTGTGCAGGTGGTGCATGGCTGTCGTCAGCTCGTGTCGTGAGATGTTGGGTTAAGTCCCGCAACGAGCGCAACCCTTGTCTCATGTTGCCAGCGGGTAATGCCGGGGACTCGTGAGAGACTGCCGGGGTCAACTCGGAGGAAGGTGGGGATGACGTCAAGTCATCATGCCCCTTATGTCCAGGGCTTCACACATGCTACAATGGCCGGTACAAAGGGCTGCGATGCCGCAAGGTTAAGCGAATCCTT-TTAAAGCCGGTCTCAGTTCGGATCGGGGTCTGCAACTCGACCCCGTGAAGTCGGAGTCGCTAGTAATCGCAGATCAGCAACGCTGCGGTGAATACGTTCCCGGGCCTTGTACACACCGCCCGTCACGTCATGAAAGTCGGTAACACCCGAAGCCAGTGGCCTAACCCTT---TGGGAGGGAGCTGTCGAAGGTGGGATCGGCGATTGGGACGAAGTCGTAACAAGGTAGCCGTACCGGAAGGTGCGGCTGGATCACCTCCTTTCTAA---->M._scrofulaceum---------TTGTTTGGAGAGTTTGATCCTGGCTCAGGACGAACGCTGGCGGCGTGCTTAACACATGCAAGTCGAACGGAAAGGCCCCT------------T--C---------------GGGGGTACTCGAGTGGCGAACGGGTGAGTAACACGTGGGCAATCTGCCCTGCACTTC-GGGATAAGCCTGGGAAACTGGGTCTAATACCGGATAGGACCA-C-TTGGCGCATGCCT-T-GTGGTGGAAAG--CTT--T----T----GCGGTGTGGGATGGGCCCGCGGCCTATCAGCTAGTTGGTGGGGTGATGGCCTACCAAGGCGACGACGGGTAGCCGGCCTGAGAGGGTGTCCGGCCACACTGGGACTGAGATACGGCCCAGACTCCTACGGGAGGCAGCAGTGGGGAATATTGCACAATGGGCGCAAGCCTGATGCAGCGACGCCGCGTGGGGGATGACGGCCTTCGGGTTGTAAACCTCTTTCACCATCGACGAAGGCTCAC-TTT-----GT-GGGTTGACGGTAGGTGGAGAAGAAGCACCGGCCAACTACGTGCCAGCAGCCGCGGTAATACGTAGGGTGCGAGCGTTGTCCGGAATTACTGGGCGTAAAGAGCTCGTAGGTGGTTTGTCGCGTTGTTCGTGAAATCTC-ACGGCTTAACTGTGAGCGTGCGGGCGATACGGGCAGACTAGAGTACTGCAGGGGAGACTGGAATTCCTGGTGTAGCGGTGGAATGCGCAGATATCAGGAGGAACACCGGTGGCGAAGGCGGGTCTCTGGGCAGTAACTGACGCTGAGGAGCGAAAGCGTGGGGAGCGAACAGGATTAGATACCCTGGTAGTCCACGCCGTAAACGGTGGGTACTAGGTGTGGGTTTCCTTCCTT--GGGATCCGTGCCGTAGCTAACGCATTAAGTACCCCGCCTGGGGAGTACGGCCGCAAGGCTAAAACTCAAAGGAATTGACGGGGGCCCGCACAAGCGGCGGAGCATGTGGATTAATTCGATGCAACGCGAAGAACCTTACCTGGGTTTGACATGCACAGGACGCGTCTAGAGATAGGCGTTCCCTTGTGGCCTGTGTGCAGGTGGTGCATGGCTGTCGTCAGCTCGTGTCGTGAGATGTTGGGTTAAGTCCCGCAACGAGCGCAACCCTTGTCTCATGTTGCCAGCGGGTAATGCCGGGGACTCGTGAGAGACTGCCGGGGTCAACTCGGAGGAAGGTGGGGATGACGTCAAGTCATCATGCCCCTTATGTCCAGGGCTTCACACATGCTACAATGGCCGGTACAAAGGGCTGCGATGCCGCAAGGTTAAGCGAATCCTT-TTAAAGCCGGTCTCAGTTCGGATCGGGGTCTGCAACTCGACCCCGTGAAGTCGGAGTCGCTAGTAATCGCAGATCAGCAACGCTGCGGTGAATACGTTCCCGGGCCTTGTACACACCGCCCGTCACGTCATGAAAGTCGGTAACACCCGAAGCCAGTGGCCTAACCCT----TGGGAGGGAGCTGTCGAAGGTGGGATCGGCGATTGGGACGAAGTCGTAACAAGGTAGCCGTACCGGAAGGTGCGGCTGGATCACCTCCTTTCTAA---->M._seoulense---------TTGTTTGGAGAGTTTGATCCTGGCTCAGGACGAACGCTGGCGGCGTGCTTAACACATGCAAGTCGAACGGAAAGGCCCCT------------T--C---------------GGGGGTACTCGAGTGGCGAACGGGTGAGTAACACGTGGGCAATCTGCCCTGCACTTC-GGGATAAGCCTGGGAAACTGGGTCTAATACCGAATAGGACCA-C-TTGGCGCATGCCT-T-GTGGTGGAAAG--CTT--T----T----GCGGTGTGGGATGGGCCCGCGGCCTATCAGCTTGTTGGTGGGGTGATGGCCTACCAAGGCGACGACGGGTAGCCGGCCTGAGAGGGTGTCCGGCCACACTGGGACTGAGATACGGCCCAGACTCCTACGGGAGGCAGCAGTGGGGAATATTGCACAATGGGCGCAAGCCTGATGCAGCGACGCCGCGTGGGGGATGACGGCCTTCGGGTTGTAAACCTCTTTCACCATCGACGAAGGTCCGG-GT-T--TCTC-GGATTGACGGTAGGTGGAGAAGAAGCACCGGCCAACTACGTGCCAGCAGCCGCGGTAATACGTAGGGTGCGAGCGTTGTCCGGAATTACTGGGCGTAAAGAGCTCGTAGGTGGTTTGTCGCGTTGTTCGTGAAATCTC-ACGGCTTAACTGTGAGCGTGCGGGCGATACGGGCAGACTAGAGTACTGCAGGGGAGACTGGAATTCCTGGTGTAGCGGTGGAATGCGCAGATATCAGGAGGAACACCGGTGGCGAAGGCGGGTCTCTGGGCAGTAACTGACGCTGAGGAGCGAAAGCGTGGGGAGCGAACAGGATTAGATACCCTGGTAGTCCACGCCGTAAACGGTGGGTACTAGGTGTGGGTTTCCTTCCTT--GGGATCCGTGCCGTAGCTAACGCATTAAGTACCCCGCCTGGGGAGTACGGCCGCAAGGCTAAAACTCAAAGGAATTGACGGGGGCCCGCACAAGCGGCGGAGCATGTGGATTAATTCGATGCAACGCGAAGAACCTTACCTGGGTTTGACATGCACAGGACGCGTCTAGAGATAGGCGTTCCCTTGTGGCCTGTGTGCAGGTGGTGCATGGCTGTCGTCAGCTCGTGTCGTGAGATGTTGGGTTAAGTCCCGCAACGAGCGCAACCCTTGTCTCATGTTGCCAGCGGGTAATGCCGGGGACTCGTGAGAGACTGCCGGGGTCAACTCGGAGGAAGGTGGGGATGACGTCAAGTCATCATGCCCCTTATGTCCAGGGCTTCACACATGCTACAATGGCCGGTACAAAGGGCTGCGATGCCGCAAGGTTAAGCGAATCCTT-TTAAAGCCGGTCTCAGTTCGGATCGGGGTCTGCAACTCGACCCCGTGAAGTCGGAGTCGCTAGTAATCGCAGATCAGCAACGCTGCGGTGAATACGTTCCCGGGCCTTGTACACACCGCCCGTCACGTCATGAAAGTCGGTAACACCCGAAGCCAGTGGCCTAACCCT----TGGGAGGGAGCTGTCGAAGGTGGGATCGGCGATTGGGACGAAGTCGTAACAAGGTAGCCGTACCGGAAGGTGCGGCTGGATCACCTCCTTTCTAAGGAG>M._shigaense----------TGTTTGGAGAGTTTGATCCTGGCTCAGGACGAACGCTGGCGGCGTGCTTAACACATGCAAGTCGAACGGAAAGGCCCCT------------T--C---------------GGGGGTACTCGAGTGGCGAACGGGTGAGTAACACGTGGGTAATCTGCCCTGCACTTC-GGGATAAGCCTGGGAAACTGGGTCTAATACCGGATAGGACCA-C-TTAGCGCATGCTT-T-GTGGTGGAAAG--CTT--T----T----GCGGTGTGGGATGGGCCCGCGGCCTATCAGCTTGTTGGTGGGGTGACGGCCTACCAAGGCGACGACGGGTAGCCGGCCTGAGAGGGTGTCCGGCCACACTGGGACTGAGATACGGCCCAGACTCCTACGGGAGGCAGCAGTGGGGAATATTGCACAATGGGCGCAAGCCTGATGCAGCGACGCCGCGTGGGGGATGACGGCCTTCGGGTTGTAAACCTCTTTCAGCAGGGACGAAGCGC----------------AAGTGACGGTACCTGCAGAAGAAGCACCGGCCAACTACGTGCCAGCAGCCGCGGTAATACGTAGGGTGCGAGCGTTGTCCGGAATTACTGGGCGTAAAGAGCTCGTAGGTGGTTTGTCGCGTTGTTCGTGAAAACCG-GGGGCTTAACCCTCGGCGTGCGGGCGATACGGGCAGACTGGAGTACTGCAGGGGAGACTGGAATTCCTGGTGTAGCGGTGGAATGCGCAGATATCAGGAGGAACACCGGTGGCGAAGGCGGGTCTCTGGGCAGTAACTGACGCTGAGGAGCGAAAGCGTGGGGAGCGAACAGGATTAGATACCCTGGTAGTCCACGCCGTAAACGGTGGGTACTAGGTGTGGGTTTCCTTCCTT--GGAATCCGTGCCGTAGCTAACGCATTAAGTACCCCGCCTGGGGAGTACGGCCGCAAGGCTAAAACTCAAAGGAATTGACGGGGGCCCGCACAAGCGGCGGAGCATGTGGATTAATTCGATGCAACGCGAAGAACCTTACCTGGGTTTGACATGCACAGGACGCCGGCAGAGATGTCGGTTCCCTTGTGGCCTGTGTGCAGGTGGTGCATGGCTGTCGTCAGCTCGTGTCGTGAGATGTTGGGTTAAGTCCCGCAACGAGCGCAACCCTTGTCTCATGTTGCCAGCGCGTAATGGCGGGGACTCGTGAGAGACTGCCGGGGTCAACTCGGAGGAAGGTGGGGATGACGTCAAGTCATCATGCCCCTTATGTCCAGGGCTTCACACATGCTACAATGGCCGGTACAAAGGGCTGCGATGCCGCAAGGTTAAGCGAATCCTT-TTAAAGCCGGTCTCAGTTCGGATCGGGGTCTGCAACTCGACCCCGTGAAGTCGGAGTCGCTAGTAATCGCAGATCAGCAACGCTGCGGTGAATACGTTCCCGGGCCTTGTACACACCGCCCGTCACGTCATGAAAGTCGGTAACACCCGAAGCCAGTGGCCTAACCTT----TTGGAGGGAGCTGTCGAAGGTGGGATCGGCGATTGGGACGAAGTCGTAACAAGGTAGCCGTACCGGAAGGTGCGGCTGGATCACCTCCTTT-------->M._shimoideiG----GTTTTTGTTTGGAGAGTTTGATCCTGGCTCAGGACGAACGCTGGCGGCGTGCTTAACACATGCAAGTCGAACGGAAAGGCCCCT------------TT-CG--------------GGGGGTACTCGAGTGGCGAACGGGTGAGTAACACGTGGGTGATCTGCCCTGCACTTC-GGGATAAGCTTGGGAAACTGGGTCTAATACCGGATAGGACCA-C-ATGTCGCATGGTG-T-GTGGTGGAAAG--CTT--T----T----GCGGTGTGGGATGGGCCCGCGGCCTATCAGCTTGTTGGTGGGGTGATGGCCTACCAAGGCGACGACGGGTAGCCGGCCTGAGAGGGTGTCCGGCCACACTGGGACTGAGATACGGCCCAGACTCCTACGGGAGGCAGCAGTGGGGAATATTGCACAATGGGCGCAAGCCTGATGCAGCGACGCCGCGTGGGGGATGACGGCCTTCGGGTTGTAAACCTCTTTCACCATCGACGAAGCTGCGG-GTTT--TCTC-GTGGTGACGGTAGGTGGAGAAGAAGCACCGGCCAACTACGTGCCAGCAGCCGCGGTAATACGTAGGGTGCGAGCGTTGTCCGGAATTACTGGGCGTAAAGAGCTCGTAGGTGGTTTGTCGCGTTGTTCGTGGAATGCC-ACAGCTTAACTGTGGGCGTGCGGGCGATACGGGCAGACTGGAGTACTGCAGGGGAGACTGGAATTCCTGGTGTAGCGGTGGAATGCGCAGATATCAGGAGGAACACCGGTGGCGAAGGCGGGTCTCTGGGCAGTAACTGACGCTGAGGAGCGAAAGCGTGGGGAGCGAACAGGATTAGATACCCTGGTAGTCCACGCCGTAAACGGTGGGTACTAGGTGTGGGT-TCTTTCCTA--AGGATCCGTGCCGTAGCTAACGCATTAAGTACCCCGCCTGGGGAGTACGGCCGCAAGGCTAAAACTCAAAGGAATTGACGGGGGCCCGCACAAGCGGCGGAGCATGTGGATTAATTCGATGCAACGCGAAGAACCTTACCTGGGTTTGACATGCACAGGACGCGTCTAGAGATAGGCGTTCCCTTGTGGCCTGTGTGCAGGTGGTGCATGGCTGTCGTCAGCTCGTGTCGTGAGATGTTGGGTTAAGTCCCGCAACGAGCGCAACCCTTGTCTCATGTTGCCAGCGCGTGATGGCGGGGACTCGTGAGAGACTGCCGGGGTCAACTCGGAGGAAGGTGGGGATGACGTCAAGTCATCATGCCCCTTATGTCCAGGGCTTCACACATGCTACAATGGCCGGTACAAAGGGCTGCGATGCCGTGAGGTTAAGCGAATCCTTGTTAAAGCCGGTCTCAGTTCGGATCGGGGTCTGCAACTCGACCCCGTGAAGTCGGAGTCGCTAGTAATCGCAGATCAGCAACGCTGCGGTGAATACGTTCCCGGGCCTTGTACACACCGCCCGTCACGTCATGAAAGTCGGTAACACCCGAAGCCGGTGGCCTAACCCGT--TTGGGAGGGAGCCGTCGAAGGTGGGATCGGCGATTGGGACGAAGTCGTAACAAGGTAGCCGTACCGGAAGGTGCGGCTGGATCACCTCCTTTCTAA---->M._shinjukuense---------TTGTTTGGAGAGTTTGATCCTGGCTCAGGACGAACGCTGGCGGCGTGCTTAACACATGCAAGTCGAACGGAAAGGTCTCT------------T--C---------------GGAGACACTCGAGTGGCGAACGGGTGAGTAACACGTGGGTGATCTGCCCTGCACACC-GGGATAAGCCTGGGAAACTGGGTCTAATACCGGATAGGACCA-C-GGGATGCATGTCT-T-GTGGTGGAAAG--CTT--T----T----GCGGTGTGGGATGAGCCCGCGGCCTATCAGCTTGTTGGTGGGGTGATGGCCTACCAAGGCGACGACGGGTAGCCGGCCTGAGAGGGTGTCCGGCCACACTGGGACTGAGATACGGCCCAGACTCCTACGGGAGGCAGCAGTGGGGAATATTGCACAATGGGCGCAAGCCTGATGCAGCGACGCCGCGTGGGGGATGACGGCCTTCGGGTTGTAAACCTCTTTCACCATCGACGAAGGTCCGG-GTTT--TCTC-GGGCTGACGGTAGGTGGAGAAGAAGCACCGGCCAACTACGTGCCAGCAGCCGCGGTAATACGTAGGGTGCGAGCGTTGTCCGGAATTACTGGGCGTAAAGAGCTCGTAGGTGGTTTGTCGCGTTGTTCGTGAAATCTC-ACGGCTTAACTGTGAGCGTGCGGGCGATACGGGCAGACTAGAGTACTGCAGGGGAGACTGGAATTCCTGGTGTAGCGGTGGAATGCGCAGATATCAGGAGGAACACCGGTGGCGAAGGCGGGTCTCTGGGCAGTAACTGACGCTGAGGAGCGAAAGCGTGGGGAGCGAACAGGATTAGATACCCTGGTAGTCCACGCCGTAAACGGTGGGTACTAGGTGTGGGTTCTTTCCTTA--AGGATCCGTGCCGTAGCTAACGCATTAAGTACCCCGCCTGGGGAGTACGGCCGCAAGGCTAAAACTCAAAGGAATTGACGGGGGCCCGCACAAGCGGCGGAGCATGTGGATTAATTCGATGCAACGCGAAGAACCTTACCTGGGTTTGACATGCACAGGACGCGTCTAGAGATAGGCGTTCCCTTGTGGCCTGTGTGCAGGTGGTGCATGGCTGTCGTCAGCTCGTGTCGTGAGATGTTGGGTTAAGTCCCGCAACGAGCGCAACCCTTGTCTCATGTTGCCAGCACGTCATGGTGGGGACTCGTGAGAGACTGCCGGGGTCAACTCGGAGGAAGGTGGGGATGACGTCAAGTCATCATGCCCCTTATGTCCAGGGCTTCACACATGCTACAATGGCCGGTACAAAGGGCTGCGATGCCGCAAGGTTAAGCGAATCCTT-CTAAAGCCGGTCTCAGTTCGGATCGGGGTCTGCAACTCGACCCCGTGAAGTCGGAGTCGCTAGTAATCGCAGATCAGCAACGCTGCGGTGAATACGTTCCCGGGCCTTGTACACACCGCCCGTCACGTCATGAAAGTCGGTAACACCCGAAGCCAGTGGCCTAACCCGC--AAGGGAGGGAGCTGTCGAAGGTGGGATCGGCGATTGGGACGAAGTCGTAACAAGGTAGCCGTACCGGAAGGTGCGGCTGGATCACCTCCTTTCTAAGGAG>M._shottsii----------TGTTTGGAGAGTTTGATCCTGGCTCAGGACGAACGCTGGCGGCGTGCTTAACACATGCAAGTCGAACGGAAAGGTCTCC------------T--C---------------GGAGATACTCGAGTGGCGAACGGGTGAGTAACACGTGGGCGATCTGCCCTGCACTTC-GGGATAAGCCTGGGAAACTGGGTCTAATACCGGATAGGACCA-C-GGGATTCATGTCC-T-GTGGTGGAAAG--CTT--T----T----GCGGTGTGGGATGAGCCCGCGGCCTATCAGCTTGTTGGTGGGGTAACGGCCTACCAAGGCGACGACGGGTAGCCGGCCTGAGAGGGTGTCCGGCCACACTGGGACTGAGATACGGCCCAGACTCCTACGGGAGGCAGCAGTGGGGAATATTGCACAATGGGCGCAAGCCTGATGCAGCGACGCCGCGTGGGGGATGACGGCCTTCGGGTTGTAAACCTCTTTCACCATCGACGAAGGTTCGG-GTTT--TCTC-GGATTGACGGTAGGTGGAGAAGAAGCACCGGCCAACTACGTGCCAGCAGCCGCGGTAATACGTAGGGTGCGAGCGTTGTCCGGAATTACTGGGCGTAAAGAGCTCGTAGGTGGTTTGTCGCGTTGTTCGTGAAATCTC-ACGGCTTAACTGTGAGCGTGCGGGCGATACGGGCAGACTAGAGTACTGCAGGGGAGACTGGAATTCCTGGTGTAGCGGTGGAATGCGCAGATATCAGGAGGAACACCGGTGGCGAAGGCGGGTCTCTGGGCAGTAACTGACGCTGAGGAGCGAAAGCGTGGGGAGCGAACAGGATTAGATACCCTGGTAGTCCACGCCGTAAACGGTGGGTACTAGGTGTGGGTTTCCTTCCTT--GGGATCCGTGCCGTAGCTAACGCATTAAGTACCCCGCCTGGGGAGTACGGCCGCAAGGCTAAAACTCAAAGGAATTGACGGGGGCCCGCACAAGCGGCGGAGCATGTGGATTAATTCGATGCAACGCGAAGAACCTTACCTGGGTTTGACATGCACAGGACGCGTCTAGAGATAGGCGTTCCCTTGTGGCCTGTGTGCAGGTGGTGCATGGCTGTCGTCAGCTCGTGTCGTGAGATGTTGGGTTAAGTCCCGCAACGAGCGCAACCCTTGTCTCATGTTGCCAGCACGTAATGGTGGGGACTCGTGAGAGACTGCCGGGGTCAACTCGGAGGAAGGTGGGGATGACGTCAAGTCATCATGCCCCTTATGTCCAGGGCTTCACACATGCTACAATGGCCGGTACAAAGGGCTGCGATGCCGCGAGGTTAAGCGAATCCTT--TAAAGCCGGTCTCAGTTCGGATCGGGGTCTGCAACTCGACCCCGTGAAGTCGGAGTCGCTAGTAATCGCAGATCAGCAACGCTGCGGTGAATACGTTCCCGGGCCTTGTACACACCGCCCGTCACGTCATGAAAGTCGGTAACACCCGAAGCCAGTGGCTTAACCCTT---TGGGAGGGAGCTGTCGAAGGTGGGATCGGCGATTGGGACGAAGTCGTAACAAGGTAGCCGTACCGGAAGGTGCGGCTGGATCACCTCCTTT-------->M._simiae---------TTGTTTGGAGAGTTTGATCCTGGCTCAGGACGAACGCTGGCGGCGTGCTTAACACATGCAAGTCGAACGGAAAGGCCCCT------------T--C---------------GGGGGTACTCGAGTGGCGAACGGGTGAGTAACACGTGGGTAATCTGCCCTGCACTTC-GGGATAAGCCTGGGAAACTGGGTCTAATACCGGATAGGACCA-C-TTGGCGCATGCCT-T-GTGGTGGAAAG--CTT--T----T----GCGGTGTGGGATGGGCCCGCGGCCTATCAGCTTGTTGGTGGGGTGACGGCCTACCAAGGCGACGACGGGTAGCCGGCCTGAGAGGGTGTCCGGCCACACTGGGACTGAGATACGGCCCAGACTCCTACGGGAGGCAGCAGTGGGGAATATTGCACAATGGGCGCAAGCCTGATGCAGCGACGCCGCGTGGGGGATGACGGCCTTCGGGTTGTAAACCTCTTTCAGCAGGGACGAAGCGC----------------AAGTGACGGTACCTGCAGAAGAAGCACCGGCCAACTACGTGCCAGCAGCCGCGGTAATACGTAGGGTGCGAGCGTTGTCCGGAATTACTGGGCGTAAAGAGCTCGTAGGTGGTTTGTCGCGTTGTTCGTGAAAACCG-GGGGCTTAACCCTCGGCGTGCGGGCGATACGGGCAGACTGGAGTACTGCAGGGGAGACTGGAATTCCTGGTGTAGCGGTGGAATGCGCAGATATCAGGAGGAACACCGGTGGCGAAGGCGGGTCTCTGGGCAGTAACTGACGCTGAGGAGCGAAAGCGTGGGGAGCGAACAGGATTAGATACCCTGGTAGTCCACGCCGTAAACGGTGGGTACTAGGTGTGGGTTTCCTTCCTT--GGAATCCGTGCCGTAGCTAACGCATTAAGTACCCCGCCTGGGGAGTACGGCCGCAAGGCTAAAACTCAAAGGAATTGACGGGGGCCCGCACAAGCGGCGGAGCATGTGGATTAATTCGATGCAACGCGAAGAACCTTACCTGGGTTTGACATGCACAGGACGCCGGCAGAGATGTCGGTTCCCTTGTGGCCTGTGTGCAGGTGGTGCATGGCTGTCGTCAGCTCGTGTCGTGAGATGTTGGGTTAAGTCCCGCAACGAGCGCAACCCTTGTCTCATGTTGCCAGCGGGTAATGCCGGGGACTCGTGAGAGACTGCCGGGGTCAACTCGGAGGAAGGTGGGGATGACGTCAAGTCATCATGCCCCTTATGTCCAGGGCTTCACACATGCTACAATGGCCGGTACAAAGGGCTGCGATGCCGCAAGGTTAAGCGAATCCTT-TTAAAGCCGGTCTCAGTTCGGATCGGGGTCTGCAACTCGACCCCGTGAAGTCGGAGTCGCTAGTAATCGCAGATCAGCAACGCTGCGGTGAATACGTTCCCGGGCCTTGTACACACCGCCCGTCACGTCATGAAAGTCGGTAACACCCGAAGCCAGTGGCCTAACCTT----TTGGAGGGAGCTGTCGAAGGTGGGATCGGCGATTGGGACGAAGTCGTAACAAGGTAGCCGTACCGGAAGGTGCGGCTGGATCACCTCCTTTCTAA---->M._smegmatis Strain mc2 155T------TTTTGTTTGGAGAGTTTGATCCTGGCTCAGGACGAACGCTGGCGGCGTGCTTAACACATGCAAGTCGAACGGAAAGGCCCTT------------T--C---------------GGGGGTACTCGAGTGGCGAACGGGTGAGTAACACGTGGGTGATCTGCCCTGCACTTT-GGGATAAGCCTGGGAAACTGGGTCTAATACCGAATACACCCTGC-TGGTCGCATGGCCTG-GTAGGGGAAAG--CTT--T----T----GCGGTGTGGGATGGGCCCGCGGCCTATCAGCTTGTTGGTGGGGTGATGGCCTACCAAGGCGACGACGGGTAGCCGGCCTGAGAGGGTGACCGGCCACACTGGGACTGAGATACGGCCCAGACTCCTACGGGAGGCAGCAGTGGGGAATATTGCACAATGGGCGCAAGCCTGATGCAGCGACGCCGCGTGAGGGATGACGGCCTTCGGGTTGTAAACCTCTTTCAGCACAGACGAAGCGC----------------AAGTGACGGTATGTGCAGAAGAAGGACCGGCCAACTACGTGCCAGCAGCCGCGGTAATACGTAGGGTCCGAGCGTTGTCCGGAATTACTGGGCGTAAAGAGCTCGTAGGTGGTTTGTCGCGTTGTTCGTGAAAACTC-ACAGCTTAACTGTGGGCGTGCGGGCGATACGGGCAGACTAGAGTACTGCAGGGGAGACTGGAATTCCTGGTGTAGCGGTGGAATGCGCAGATATCAGGAGGAACACCGGTGGCGAAGGCGGGTCTCTGGGCAGTAACTGACGCTGAGGAGCGAAAGCGTGGGGAGCGAACAGGATTAGATACCCTGGTAGTCCACGCCGTAAACGGTGGGTACTAGGTGTGGGTTTCCTTCCTT--GGGATCCGTGCCGTAGCTAACGCATTAAGTACCCCGCCTGGGGAGTACGGCCGCAAGGCTAAAACTCAAAGGAATTGACGGGGGCCCGCACAAGCGGCGGAGCATGTGGATTAATTCGATGCAACGCGAAGAACCTTACCTGGGTTTGACATGCACAGGACGCCGGCAGAGATGTCGGTTCCCTTGTGGCCTGTGTGCAGGTGGTGCATGGCTGTCGTCAGCTCGTGTCGTGAGATGTTGGGTTAAGTCCCGCAACGAGCGCAACCCTTGTCTCATGTTGCCAGCACGTTATGGTGGGGACTCGTGAGAGACTGCCGGGGTCAACTCGGAGGAAGGTGGGGATGACGTCAAGTCATCATGCCCCTTATGTCCAGGGCTTCACACATGCTACAATGGCCGGTACAAAGGGCTGCGATGCCGTGAGGTGGAGCGAATCCTT-TCAAAGCCGGTCTCAGTTCGGATCGGGGTCTGCAACTCGACCCCGTGAAGTCGGAGTCGCTAGTAATCGCAGATCAGCAACGCTGCGGTGAATACGTTCCCGGGCCTTGTACACACCGCCCGTCACGTCATGAAAGTCGGTAACACCCGAAGCCGGTGGCCTAACCCTT---GTGGAGGGAGCCGTCGAAGGTGGGATCGGCGATTGGGACGAAGTCGTAACAAGGTAGCCGTACCGGAAGGTGCGGCTGGATCACCTCCTTTCT------>M._sp._155---------TTGTTTGGAGAGTTTGATCCTGGCTCAGGACGAACGCTGGCGGCGTGCTTAACACATGCAAGTCGAACGGAAAGGCCC-T------------T--C----------------GGGGTGCTCGAGTGGCGAACGGGTGAGTAACACGTGGGTGATCTGCCCTGCACTTT-GGGATAAGCCTGGGAAACTGGGTCTAATACCGAATAGGACTC-C-GGACTGCATGGTC-T-GGGGTGGAAAG--CTT--T----T----GCGGTGTGGGATGGGCCCGCGGCCTATCAGCTTGTTGGTGGGGTGATGGCCTACCAAGGCGACGACGGGTAGCCGGCCTGAGAGGGTGACCGGCCACACTGGGACTGAGATACGGCCCAGACTCCTACGGGAGGCAGCAGTGGGGAATATTGCACAATGGGCGCAAGCCTGATGCAGCGACGCCGCGTGGGGGATGACGGCCTTCGGGTTGTAAACCTCTTTCAGCACAGACGAAGCGC----------------AAGTGACGGTATGTGCAGAAGAAGCACCGGCCAACTACGTGCCAGCAGCCGCGGTAATACGTAGGGTGCGAGCGTTGTCCGGAATTACTGGGCGTAAAGAGCTCGTAGGTGGTTTGTCGCGTTGTTCGTGAAAACTC-ACAGCTTAACTGTGGGCGTGCGGGCGATACGGGCAGACTGGAGTACTGCAGGGGAGACTGGAATTCCTGGTGTAGCGGTGGAATGCGCAGATATCAGGAGGAACACCGGTGGCGAAGGCGGGTCTCTGGGCAGTAACTGACGCTGAGGAGCGAAAGCGTGGGGAGCGAACAGGATTAGATACCCTGGTAGTCCACGCCGTAAACGGTGGGTACTAGGTGTGGGTTTCCTTCCTT--GGGATCCGTGCCGTAGCTAACGCATTAAGTACCCCGCCTGGGGAGTACGGCCGCAAGGCTAAAACTCAAAGGAATTGACGGGGGCCCGCACAAGCGGCGGAGCATGTGGATTAATTCGATGCAACGCGAAGAACCTTACCTGGGTTTGACATGCACAGGACGCCGGTAGAGATATCGGTTCCCTTGTGGCCTGTGTGCAGGTGGTGCATGGCTGTCGTCAGCTCGTGTCGTGAGATGTTGGGTTAAGTCCCGCAACGAGCGCAACCCTTGTCCTATGTTGCCAGCGGGTTATGCCGGGGACTCGTAGGAGACTGCCGGGGTCAACTCGGAGGAAGGTGGGGATGACGTCAAGTCATCATGCCCCTTATGTCCAGGGCTTCACACATGCTACAATGGCCGGTACAAAGGGCTGCGATGCCGTGAGGTGGAGCGAATCCTT-TCAAAGCCGGTCTCAGTTCGGATCGGGGTCTGCAACTCGACCCCGTGAAGTCGGAGTCGCTAGTAATCGCAGATCAGCAACGCTGCGGTGAATACGTTCCCGGGCCTTGTACACACCGCCCGTCACGTCATGAAAGTCGGTAACACCCGAAGCCGGTGGCCTAACCCCT-TGTGGGAGGGAGCCGTCGAAGGTGGGATCGGCGATTGGGACGAAGTCGTAACAAGGTAGCCGTACCGGAAGGTGCGGCTGGATCACCTCCTTTCTAA---->M._sp._EPa45---------TTGTTTGGAGAGTTTGATCCTGGCTCAGGACGAACGCTGGCGGCGTGCTTAACACATGCAAGTCGAACGGAAAGTGCTCT------------T--C---------------GGAGTGACTCGAGTGGCGAACGGGTGAGTAACACGTGGGTGATCTGCCCTGCACTTT-GGGATAAGCCTGGGAAACTGGGTCTAATACCGAATAGGACCG-C-ATGCTTCATGGTG-T-GTGGTGGAAAG--CTT--T----T----GCGGTGTGGGATGGGCCCGCGGCCTATCAGCTTGTTGGTGGGGTAATGGCCTACCAAGGCGACGACGGGTAGCCGGCCTGAGAGGGTGTCCGGCCACACTGGGACTGAGATACGGCCCAGACTCCTACGGGAGGCAGCAGTGGGGAATATTGCACAATGGGCGCAAGCCTGATGCAGCGACGCCGCGTGAGGGATGACGGCCTTCGGGTTGTAAACCTCTTTCAGTAGGGACGAAGCGT----------------AAGTGACGGTACCTATAGAAGAAGCACCGGCCAACTACGTGCCAGCAGCCGCGGTAATACGTAGGGTGCGAGCGTTGTCCGGAATTACTGGGCGTAAAGAGCTCGTAGGTGGTTTGTCGCGTTGTTCGTGAAAACTC-ACAGCTTAACTGTGGGCGTGCGGGCGATACGGGCAGACTGGAGTACTGCAGGGGAGACTGGAATTCCTGGTGTAGCGGTGGAATGCGCAGATATCAGGAGGAACACCGGTGGCGAAGGCGGGTCTCTGGGCAGTAACTGACGCTGAGGAGCGAAAGCGTGGGGAGCGAACAGGATTAGATACCCTGGTAGTCCACGCCGTAAACGGTGGGTACTAGGTGTGGGTTTCCTTCCTT--GGGATCCGTGCCGTAGCTAACGCATTAAGTACCCCGCCTGGGGAGTACGGCCGCAAGGCTAAAACTCAAAGGAATTGACGGGGGCCCGCACAAGCGGCGGAGCATGTGGATTAATTCGATGCAACGCGAAGAACCTTACCTGGGTTTGACATGCACAGGACGCCGGCAGAGATGTCGGTTCCCTTGTGGCCTGTGTGCAGGTGGTGCATGGCTGTCGTCAGCTCGTGTCGTGAGATGTTGGGTTAAGTCCCGCAACGAGCGCAACCCTTGTCTCATGTTGCCAGCACGTTATGGTGGGGACTCGTGAGAGACTGCCGGGGTCAACTCGGAGGAAGGTGGGGATGACGTCAAGTCATCATGCCCCTTATGTCCAGGGCTTCACACATGCTACAATGGCCGGTACAAAGGGCTGCGATGCCGTGAGGTGGAGCGAATCCTT-TCAAAGCCGGTCTCAGTTCGGATCGGGGTCTGCAACTCGACCCCGTGAAGTCGGAGTCGCTAGTAATCGCAGATCAGCAACGCTGCGGTGAATACGTTCCCGGGCCTTGTACACACCGCCCGTCACGTCATGAAAGTCGGTAACACCCGAAGCCGGTGGCCTAACCCCT-TGTGGGAGGGAGCCGTCGAAGGTGGGATCGGCGATTGGGACGAAGTCGTAACAAGGTAGCCGTACCGGAAGGTGCGGCTGGATCACCTCCTTTCTAA---->M._sp._GA-0227b---------TTGTTTGGAGAGTTTGATCCTGGCTCAGGACGAACGCTGGCGGCGTGCTTAACACATGCAAGTCGAACGGAAAGGCCCTT------------T--C---------------GGGGGTACTCGAGTGGCGAACGGGTGAGTAACACGTGGGTGATCTGCCCTGCACTTT-GGGATAAGCCTGGGAAACTGGGTCTAATACCGAATATTCCCT-GCCGGTTGCATGGTT-G-GTGGGGGAAAG--CTT--T----T----GCGGTGTGGGATGGGCCCGCGGCCTATCAGCTTGTTGGTGGGGTGATGGCCTACCAAGGCGACGACGGGTAGCCGGCCTGAGAGGGTGTCCGGCCACACTGGGACTGAGATACGGCCCAGACTCCTACGGGAGGCAGCAGTGGGGAATATTGCACAATGGGCGCAAGCCTGATGCAGCGACGCCGCGTGGGGGATGACGGCCTTCGGGTTGTAAACCTCTTTCAGTACCGACGAAGCGG----------------AAGTGACGGTAGGTACAGAAGAAGCACCGGCCAACTACGTGCCAGCAGCCGCGGTAATACGTAGGGTGCGAGCGTTGTCCGGAATTACTGGGCGTAAAGAGCTCGTAGGTGGTTTGTCGCGTTGTCCGTGAAAACTC-ACAGCTTAACTGTGGGCGTGCGGGCGATACGGGCAGACTGGAGTACTGCAGGGGAGACTGGAATTCCTGGTGTAGCGGTGGAATGCGCAGATATCAGGAGGAACACCGGTGGCGAAGGCGGGTCTCTGGGCAGTAACTGACGCTGAGGAGCGAAAGCGTGGGGAGCGAACAGGATTAGATACCCTGGTAGTCCACGCCGTAAACGGTGGGTACTAGGTGTGGGTTTCCTTCCTT--GGGATCCGTGCCGTAGCTAACGCATTAAGTACCCCGCCTGGGGAGTACGGCCGCAAGGCTAAAACTCAAAGGAATTGACGGGGGCCCGCACAAGCGGCGGAGCATGTGGATTAATTCGATGCAACGCGAAGAACCTTACCTGGGTTTGACATGCACAGGACGCCGGCAGAGATGTCGGTTCCCTTGTGGCCTGTGTGCAGGTGGTGCATGGCTGTCGTCAGCTCGTGTCGTGAGATGTTGGGTTAAGTCCCGCAACGAGCGCAACCCTTGTCTCATGTTGCCAGCGGGTAATGCCGGGGACTCGTGAGAGACTGCCGGGGTCAACTCGGAGGAAGGTGGGGATGACGTCAAGTCATCATGCCCCTTATGTCCAGGGCTTCACACATGCTACAATGGCCGGTACAAAGGGCTGCGATGCCGTGAGGTGGAGCGAATCCTT-TCAAAGCCGGTCTCAGTTCGGATCGGGGTCTGCAACTCGACCCCGTGAAGTCGGAGTCGCTAGTAATCGCAGATCAGCAACGCTGCGGTGAATACGTTCCCGGGCCTTGTACACACCGCCCGTCACGTCATGAAAGTCGGTAACACCCGAAGCCGGTGGCCTAACCCCT-TGTGGGAGGGAGCCGTCGAAGGTGGGATCGGCGATTGGGACGAAGTCGTAACAAGGTAGCCGTACCGGAAGGTGCGGCTGGATCACCTCCTTTCTAA---->M._sp._JLSG----GGTTTTGTTTGGAGAGTTTGATTCTGGCTCAGGACGAACGCTGGCGGCGTGCTTAACACATGCAAGTCGAACGGAAAGGCCC-T------------T--C----------------GGGGTACTCGAGTGGCGAACGGGTGAGTAACACGTGGGTGATCTGCCCTGCACTTT-GGGATAAAGCCTGGGAACTGGGTCTAATACCGAATACACCCTGC-TGGTCGCATGGCCTG-GTGGGGGAAAG--CTT--T----T----GCGGTGTGGGATGGGCCCGCGGCCTATCAGCTTGTTGGTGAGGTTACGGCTCACCAAGGCGACGACGGGTAGCCGGCCTGAGAGGGTGACCGGCCACACTGGGACTGAGATACGGCCCAGACTCCTACGGGAGGCAGCAGTGGGGAATATTGCACAATGGGCGCAAGCCTGATGCAGCGACGCCGCGTGGGGGATGACGGCCTTCGGGTTGTAAACCTCTTTCAGTAGGGACGAAGCGC----------------AAGTGACGGTACCTACAGAAGAAGGACCGGCCAACTACGTGCCAGCAGCCGCGGTAATACGTAGGGTCCGAGCGTTGTCCGGAATTACTGGGCGTAAAGAGCTCGTAGGTGGTTTGTCGCGTTGTTCGTGAAAACCG-GGGGCTTAACCCTCGGCGTGCGGGCGATACGGGCAGACTTGAGTACTGCAGGGGAGACTGGAATTCCTGGTGTAGCGGTGGAATGCGCAGATATCAGGAGGAACACCGGTGGCGAAGGCGGGTCTCTGGGCAGTAACTGACGCTGAGGAGCGAAAGCGTGGGGAGCGAACAGGATTAGATACCCTGGTAGTCCACGCCGTAAACGGTGGGTACTAGGTGTGGGTTTCCTTCCTT--GGGATCCGTGCCGTAGCTAACGCATTAAGTACCCCGCCTGGGGAGTACGGCCGCAAGGCTAAAACTCAAAGAAATTGACGGGGGCCCGCACAAGCGGCGGAGCATGTGGATTAATTCGATGCAACGCGAAGAACCTTACCTGGGTTTGACATGCACAGGACGCTGGTAGAGATATCAGTTCCCTTGTGGCCTGTGTGCAGGTGGTGCATGGCTGTCGTCAGCTCGTGTCGTGAGATGTTGGGTTAAGTCCCGCAACGAGCGCAACCCTTGTCTCATGTTGCCAGCACGTTATGGTGGGGACTCGTGAGAGACTGCCGGGGTCAACTCGGAGGAAGGTGGGGATGACGTCAAGTCATCATGCCCCTTATGTCCAGGGCTTCACACATGCTACAATGGCCGGTACAAAGGGCTGCGATGCCGTGAGGTGGAGCGAATCCTT-TCAAAGCCGGTCTCAGTTCGGATCGGGGTCTGCAACTCGACCCCGTGAAGTCGGAGTCGCTAGTAATCGCAGATCAGCAACGCTGCGGTGAATACGTTCCCGGGCCTTGTACACACCGCCCGTCACGTCATGAAAGTCGGTAACACCCGAAGCCGGTGGCCTAACCCCT-TGTGGGAGGGAGCCGTCGAAGGTGGGATCGGCGATTGGGACGAAGTCGTAACAAGGTAGCCGTACCGGAAGGTGCGGCTGGATCACCTCCTTTCTAA---->M._sp._KMSG----GGTTTTGTTTGGAGAGTTTGATTCTGGCTCAGGACGAACGCTGGCGGCGTGCTTAACACATGCAAGTCGAACGGAAAGGCCC-T------------T--C----------------GGGGTGCTCGAGTGGCGAACGGGTGAGTAACACGTGGGTGATCTGCCCTGCACTTT-GGGATAAGCCTGGGAAACTGGGTCTAATACCGAATACACCCTGC-TGGTCGCATGGCCTG-GTGGGGGAAAG--CTT--T----T----GCGGTGTGGGATGGGCCCGCGGCCTATCAGCTTGTTGGTGAGGTTACGGCTCACCAAGGCGACGACGGGTAGCCGGCCTGAGAGGGTGACCGGCCACACTGGGACTGAGATACGGCCCAGACTCCTACGGGAGGCAGCAGTGGGGAATATTGCACAATGGGCGCAAGCCTGATGCAGCGACGCCGCGTGGGGGATGACGGCCTTCGGGTTGTAAACCTCTTTCAGTAGGGACGAAGCGC----------------AAGTGACGGTACCTACAGAAGAAGGACCGGCCAACTACGTGCCAGCAGCCGCGGTAATACGTAGGGTCCGAGCGTTGTCCGGAATTACTGGGCGTAAAGAGCTCGTAGGTGGTTTGTCGCGTTGTTCGTGAAAACCG-GGGGCTTAACCCTCGGCGTGCGGGCGATACGGGCAGACTTGAGTATTGCAGGGGAGACTGGAATTCCTGGTGTAGCGGTGGAATGCGCAGATATCAGGAGGAACACCGGTGGCGAAGGCGGGTCTCTGGGCAGTAACTGACGCTGAGGAGCGAAAGCGTGGGGAGCGAACAGGATTAGATACCCTGGTAGTCCACGCCGTAAACGGTGGGTACTAGGTGTGGGTTTCCTTCCTT--GGGATCCGTGCCGTAGCTAACGCATTAAGTACCCCGCCTGGGGAGTACGGCCGCAAGGCTAAAACTCAAAGAAATTGACGGGGGCCCGCACAAGCGGCGGAGCATGTGGATTAATTCGATGCAACGCGAAGAACCTTACCTGGGTTTGACATGCACAGGACGCTGGTAGAGATATCAGTTCCCTTGTGGCCTGTGTGCAGGTGGTGCATGGCTGTCGTCAGCTCGTGTCGTGAGATGTTGGGTTAAGTCCCGCAACGAGCGCAACCCTTGTCTCATGTTGCCAGCACGTTATGGTGGGGACTCGTGAGAGACTGCCGGGGTCAACTCGGAGGAAGGTGGGGATGACGTCAAGTCATCATGCCCCTTATGTCCAGGGCTTCACACATGCTACAATGGCCGGTACAAAGGGCTGCGATGCCGTGAGGTGGAGCGAATCCTT-TCAAAGCCGGTCTCAGTTCGGATCGGGGTCTGCAACTCGACCCCGTGAAGTCGGAGTCGCTAGTAATCGCAGATCAGCAACGCTGCGGTGAATACGTTCCCGGGCCTTGTACACACCGCCCGTCACGTCATGAAAGTCGGTAACACCCGAAGCCGGTGGCCTAACCCCT-TGTGGGAGGGAGCCGTCGAAGGTGGGATCGGCGATTGGGACGAAGTCGTAACAAGGTAGCCGTACCGGAAGGTGCGGCTGGATCACCTCCTTTCTAA---->M._sp._M26---------TTGTTTGGAGAGTTTGATCCTGGCTCAGGACGAACGCTGGCGGCGTGCTTAACACATGCAAGTCGAACGGTAAGGCCC-T------------T--C----------------GGGGTACACGAGTGGCGAACGGGTGAGTAACACGTGGGTGATCTGCCCTGCACTTT-GGGATAAGCCTGGGAAACTGGGTCTAATACCGAATACACCCTGC-TGGTCGCATGGCCTG-GTGGGGGAAAG--CTT--T----T----GCGGTGTGGGATGGGCCCGCGGCCTATCAGCTTGTTGGTGGGGTGATGGCCTACCAAGGCGACGACGGGTAGCCGGCCTGAGAGGGTGACCGGCCACACTGGGACTGAGATACGGCCCAGACTCCTACGGGAGGCAGCAGTGGGGAATATTGCACAATGGGCGCAAGCCTGATGCAGCGACGCCGCGTGAGGGATGACGGCCTTCGGGTTGTAAACCTCTTTCAATAGGGACGAAGCGC----------------AAGTGACGGTACCTATAGAAGAAGGACCGGCCAACTACGTGCCAGCAGCCGCGGTAATACGTAGGGTCCGAGCGTTGTCCGGAATTACTGGGCGTAAAGAGCTCGTAGGTGGTTTGTCGCGTTGTTCGTGAAAACCC-ACAGCTTAACTGTGGGCGTGCGGGCGATACGGGCAGACTTGAGTACTGCAGGGGAGACTGGAATTCCTGGTGTAGCGGTGGAATGCGCAGATATCAGGAGGAACACCGGTGGCGAAGGCGGGTCTCTGGGCAGTAACTGACGCTGAGGAGCGAAAGCGTGGGGAGCGAACAGGATTAGATACCCTGGTAGTCCACGCCGTAAACGGTGGGTACTAGGTGTGGGTTTCCTTCCTT--GGGATCCGTGCCGTAGCTAACGCATTAAGTACCCCGCCTGGGGAGTACGGCCGCAAGGCTAAAACTCAAAGGAATTGACGGGGGCCCGCACAAGCGGCGGAGCATGTGGATTAATTCGATGCAACGCGAAGAACCTTACCTGGGTTTGACATGCACAGGACGCCGGCAGAGATGTCGGTTCCCTTGTGGCCTGTGTGCAGGTGGTGCATGGCTGTCGTCAGCTCGTGTCGTGAGATGTTGGGTTAAGTCCCGCAACGAGCGCAACCCTTGTCTCATGTTGCCAGCACGTTATGGTGGGGACTCGTGAGAGACTGCCGGGGTCAACTCGGAGGAAGGTGGGGATGACGTCAAGTCATCATGCCCCTTATGTCCAGGGCTTCACACATGCTACAATGGCCGGTACAAAGGGCTGCGATGCCGTGAGGTGGAGCGAATCCTT-TCAAAGCCGGTCTCAGTTCGGATCGGGGTCTGCAACTCGACCCCGTGAAGTCGGAGTCGCTAGTAATCGCAGATCAGCAACGCTGCGGTGAATACGTTCCCGGGCCTTGTACACACCGCCCGTCACGTCATGAAAGTCGGTAACACCCGAAGCCGGTGGCCTAACCCCT-TGTGGGAGGGAGCCGTCGAAGGTGGGATCGGCGATTGGGACGAAGTCGTAACAAGGTAGCCGTACCGGAAGGTGCGGCTGGATCACCTCCTTTCTAA---->M._sp._MCSG----GGTTTTGTTTGGAGAGTTTGATTCTGGCTCAGGACGAACGCTGGCGGCGTGCTTAACACATGCAAGTCGAACGGAAAGGCCC-T------------T--C----------------GGGGTACTCGAGTGGCGAACGGGTGAGTAACACGTGGGTGATCTGCCCTGCACTTT-GGGATAAGCCTGGGAAACTGGGTCTAATACCGAATACACCCTGC-TGGTCGCATGGCCTG-GTGGGGGAAAG--CTT--T----T----GCGGTGTGGGATGGGCCCGCGGCCTATCAGCTTGTTGGTGAGGTTACGGCTCACCAAGGCGACGACGGGTAGCCGGCCTGAGAGGGTGACCGGCCACACTGGGACTGAGATACGGCCCAGACTCCTACGGGAGGCAGCAGTGGGGAATATTGCACAATGGGCGCAAGCCTGATGCAGCGACGCCGCGTGGGGGATGACGGCCTTCGGGTTGTAAACCTCTTTCAGTAGGGACGAAGCGC----------------AAGTGACGGTACCTACAGAAGAAGGACCGGCCAACTACGTGCCAGCAGCCGCGGTAATACGTAGGGTCCGAGCGTTGTCCGGAATTACTGGGCGTAAAGAGCTCGTAGGTGGTTTGTCGCGTTGTTCGTGAAAACCG-GGGGCTTAACCCTCGGCGTGCGGGCGATACGGGCAGACTTGAGTACTGCAGGGGAGACTGGAATTCCTGGTGTAGCGGTGGAATGCGCAGATATCAGGAGGAACACCGGTGGCGAAGGCGGGTCTCTGGGCAGTAACTGACGCTGAGGAGCGAAAGCGTGGGGAGCGAACAGGATTAGATACCCTGGTAGTCCACGCCGTAAACGGTGGGTACTAGGTGTGGGTTTCCTTCCTT--GGGATCCGTGCCGTAGCTAACGCATTAAGTACCCCGCCTGGGGAGTACGGCCGCAAGGCTAAAACTCAAAGAAATTGACGGGGGCCCGCACAAGCGGCGGAGCATGTGGATTAATTCGATGCAACGCGAAGAACCTTACCTGGGTTTGACATGCACAGGACGCTGGTAGAGATATCAGTTCCCTTGTGGCCTGTGTGCAGGTGGTGCATGGCTGTCGTCAGCTCGTGTCGTGAGATGTTGGGTTAAGTCCCGCAACGAGCGCAACCCTTGTCTCATGTTGCCAGCACGTTATGGTGGGGACTCGTGAGAGACTGCCGGGGTCAACTCGGAGGAAGGTGGGGATGACGTCAAGTCATCATGCCCCTTATGTCCAGGGCTTCACACATGCTACAATGGCCGGTACAAAGGGCTGCGATGCCGTGAGGTGGAGCGAATCCTT-TCAAAGCCGGTCTCAGTTCGGATCGGGGTCTGCAACTCGACCCCGTGAAGTCGGAGTCGCTAGTAATCGCAGATCAGCAACGCTGCGGTGAATACGTTCCCGGGCCTTGTACACACCGCCCGTCACGTCATGAAAGTCGGTAACACCCGAAGCCGGTGGCCTAACCCCT-TGTGGGAGGGAGCCGTCGAAGGTGGGATCGGCGATTGGGACGAAGTCGTAACAAGGTAGCCGTACCGGAAGGTGCGGCTGGATCACCTCCTTTCTAA---->M._sp._MOTT36Y----------TGTTTGGAGAGTTTGATCCTGGCTCAGGACGAACGCTGGCGGCGTGCTTAACACATGCAAGTCGAACGGAAAGGCCCCT------------T--C---------------GGGGGTACTCGAGTGGCGAACGGGTGAGTAACACGTGGGCAATCTGCCCTGCACTTC-GGGATAAGCCTGGGAAACTGGGTCTAATACCGGATAGGACCT-T-TAGGCGCATGTCT-T-TAGGTGGAAAG--CTT--T----T----GCGGTGTGGGATGGGCCCGCGGCCTATCAGCTTGTTGGTGGGGTGATGGCCTACCAAGGCGACGACGGGTAGCCGGCCTGAGAGGGTGTCCGGCCACACTGGGACTGAGATACGGCCCAGACTCCTACGGGAGGCAGCAGTGGGGAATATTGCACAATGGGCGCAAGCCTGATGCAGCGACGCCGCGTGGGGGATGACGGCCTTCGGGTTGTAAACCTCTTTCACCATCGACGAAGGTCCGG-GTTT--TCTC-GGATTGACGGTAGGTGGAGAAGAAGCACCGGCCAACTACGTGCCAGCAGCCGCGGTAATACGTAGGGTGCGAGCGTTGTCCGGAATTACTGGGCGTAAAGAGCTCGTAGGTGGTTTGTCGCGTTGTTCGTGAAATCTC-ACGGCTTAACTGTGAGCGTGCGGGCGATACGGGCAGACTAGAGTACTGCAGGGGAGACTGGAATTCCTGGTGTAGCGGTGGAATGCGCAGATATCAGGAGGAACACCGGTGGCGAAGGCGGGTCTCTGGGCAGTAACTGACGCTGAGGAGCGAAAGCGTGGGGAGCGAACAGGATTAGATACCCTGGTAGTCCACGCCGTAAACGGTGGGTACTAGGTGTGGGTTTCCTTCCTT--GGGATCCGTGCCGTAGCTAACGCATTAAGTACCCCGCCTGGGGAGTACGGCCGCAAGGCTAAAACTCAAAGGAATTGACGGGGGCCCGCACAAGCGGCGGAGCATGTGGATTAATTCGATGCAACGCGAAGAACCTTACCTGGGTTTGACATGCACAGGACGCGTCTAGAGATAGGCGTTCCCTTGTGGCCTGTGTGCAGGTGGTGCATGGCTGTCGTCAGCTCGTGTCGTGAGATGTTGGGTTAAGTCCCGCAACGAGCGCAACCCTTGTCTCATGTTGCCAGCGGGTAATGCCGGGGACTCGTGAGAGACTGCCGGGGTCAACTCGGAGGAAGGTGGGGATGACGTCAAGTCATCATGCCCCTTATGTCCAGGGCTTCACACATGCTACAATGGCCGGTACAAAGGGCTGCGATGCCGCAAGGTTAAGCGAATCCTT-TTAAAGCCGGTCTCAGTTCGGATTGGGGTCTGCAACTCGACCCCATGAAGTCGGAGTCGCTAGTAATCGCAGATCAGCAACGCTGCGGTGAATACGTTCCCGGGCCTTGTACACACCGCCCGTCACGTCATGAAAGTCGGTAACACCCGAAGCCAGTGGCCTAACCCT----TGGGAGGGAGCTGTCGAAGGTGGGATCGGCGATTGGGACGAAGTCGTAACAAGGTAGCCGTACCGGAAGGTGCGGCTGGATCACCTCCTTT-------->M._sp._QIA-37---------TTGTTTGGAGAGTTTGATCCTGGCTCAGGACGAACGCTGGCGGCGTGCTTAACACATGCAAGTCGAACGGAAAGGCCC-T------------T--C----------------GGGGTACTCGAGTGGCGAACGGGTGAGTAACACGTGGGTGATCTGCCCTGCACTCT-GGGATAAGCCTGGGAAACTGGGTCTAATACCGGATAGGACCA-C-ACACTTCATGGTG-A-GTGGTGCAAAG--CTT--T----T----GCGGTGTGGGATGAGCCCGCGGCCTATCAGCTTGTTGGTGGGGTAATGGCCCACCAAGGCGACGACGGGTAGCCGGCCTGAGAGGGTGACCGGCCACACTGGGACTGAGATACGGCCCAGACTCCTACGGGAGGCAGCAGTGGGGAATATTGCACAATGGGCGCAAGCCTGATGCAGCGACGCCGCGTGAGGGATGACGGCCTTCGGGTTGTAAACCTCTTTCAGTAGGGACGAAGCGA----------------AAGTGACGGTACCTACAGAAGAAGGACCGGCCAACTACGTGCCAGCAGCCGCGGTAATACGTAGGGTCCGAGCGTTGTCCGGAATTACTGGGCGTAAAGAGCTCGTAGGTGGTTTGTCGCGTTGTTCGTGAAAACTC-ACAGCTTAACTGTGGGCGTGCGGGCGATACGGGCAGACTAGAGTACTGCAGGGGAGACTGGAATTCCTGGTGTAGCGGTGGAATGCGCAGATATCAGGAGGAACACCGGTGGCGAAGGCGGGTCTCTGGGCAGTAACTGACGCTGAGGAGCGAAAGCGTGGGTAGCGAACAGGATTAGATACCCTGGTAGTCCACGCCGTAAACGGTGGGTACTAGGTGTGGGTTTCCTTCCTT--GGGATCCGTGCCGTAGCTAACGCATTAAGTACCCCGCCTGGGGAGTACGGTCGCAAGACTAAAACTCAAAGGAATTGACGGGGGCCCGCACAAGCGGCGGAGCATGTGGATTAATTCGATGCAACGCGAAGAACCTTACCTGGGTTTGACATGCACAGGACGTACCTAGAGATAGGTATTCCCTTGTGGCCTGTGTGCAGGTGGTGCATGGCTGTCGTCAGCTCGTGTCGTGAGATGTTGGGTTAAGTCCCGCAACGAGCGCAACCCTTGTCCTATGTTGCCAGCGGGTAATGCCGGGGACTCGTAGGAGACTGCCGGGGTCAACTCGGAGGAAGGTGGGGATGACGTCAAGTCATCATGCCCCTTATGTCCAGGGCTTCACACATGCTACAATGGCCAGTACAGAGGGCTGCGAAGCCGCAAGGTGGAGCGAATCCCT--TAAAGCTGGTCTCAGTTCGGATTGGGGTCTGCAACTCGACCCCATGAAGTCGGAGTCGCTAGTAATCGCAGATCAGCAACGCTGCGGTGAATACGTTCCCGGGCCTTGTACACACCGCCCGTCACGTCATGAAAGTCGGTAACACCCGAAGCCAGTGGCCTAACCTT----TTGGAGGGAGCTGTCGAAGGTGGGATCGGCGATTGGGACGAAGTCGTAACAAGGTAGCCGTACCGGAAGGTGCGGCTGGATCACCTCCTTTCTAA---->M._sp._Root135---------TTGTTTGGAGAGTTTGATTCTGGCTCAGGACGAACGCTGGCGGCGTGCTTAACACATGCAAGTCGAACGGAAAGGCCC-T------------T--C----------------GGGGTACTCGAGTGGCGAACGGGTGAGTAACACGTGGGTGATCTGCCCTGCACTTC-GGGATAAGCCTGGGAAACTGGGTCTAATACCGGATATGACCA-T-GRGATGCATGTCT-T-GTGGTGGAAAG--CTT--T----T----GCGGTGTGGGATGGGCCCGCGGCCTATCAGCTTGTTGGTGGGGTTAAGGCCTACCAAGGCGACGACGGGTAGCCGGCCTGAGAGGGTGACCGGCCACACTGGGACTGAGATACGGCCCAGACTCCTACGGGAGGCAGCAGTGGGGAATATTGCACAATGGGCGCAAGCCTGATGCAGCGACGCCGCGTGAGGGACGACGGCCTTCGGGTTGTAAACCTCTTTCAGCACAGACGAAGCGC----------------AAGTGACGGTATGTGCAGAAGAAGGACCGGCCAACTACGTGCCAGCAGCCGCGGTAATACGTAGGGTCCGAGCGTTGTCCGGAATTACTGGGCGTAAAGAGCTCGTAGGTGGTTTGTCGCGTTGTTCGTGAAAACTC-ACAGCTCAACTGTGGGCGTGCGGGCGATACGGGCAGACTAGAGTACTGCAGGGGAGACTGGAATTCCTGGTGTAGCGGTGGAATGCGCAGATATCAGGAGGAACACCGGTGGCGAAGGCGGGTCTCTGGGCAGTAACTGACGCTGAGGAGCGAAAGCGTGGGGAGCGAACAGGATTAGATACCCTGGTAGTCCACGCCGTAAACGGTGGGTACTAGGTGTGGGTTTCCTTCCTT--GGGATCCGTGCCGTAGCTAACGCATTAAGTACCCCGCCTGGGGAGTACGGCCGCAAGGCTAAAACTCAAAGAAATTGACGGGGGCCCGCACAAGCGGCGGAGCATGTGGATTAATTCGATGCAACGCGAAGAACCTTACCTGGGTTTGACATGCACAGGACGTGCCTAGAGATAGGTATTCCCTTGTGGCCTGTGTGCAGGTGGTGCATGGCTGTCGTCAGCTCGTGTCGTGAGATGTTGGGTTAAGTCCCGCAACGAGCGCAACCCCTATCTTATGTTGCCAGCGCGTYATGGCGGGGACTCGTAAGAGACTGCCGGGGTCAACTCGGAGGAAGGTGGGGATGACGTCAAGTCATCATGCCCCTTATGTCCAGGGCTTCACACATGCTACAATGGCCGGTACAAAGGGCTGCGATGCCGTGAGGTGGAGCGAATCCTT--TAAAGCCGGTCTCAGTTCGGATCGGGGTCTGCAACTCGACCCCGTGAAGTCGGAGTCGCTAGTAATCGCAGATCAGCAACGCTGCGGTGAATACGTTCCCGGGCCTTGTACACACCGCCCGTCACGTCATGAAAGTCGGTAACACCCGAAGCCGGTGGCCTAACCCTT---GTGGAGGGAGCCGTCGAAGGTGGGATCGGCGATTGGGACGAAGTCGTAACAAGGTAGCCGTACCGGAAGGTGCGGCTGGATCACCTCCTTTCTAA---->M._sp._Root265---------TTGTTTGGAGAGTTTGATTCTGGCTCAGGACGAACGCTGGCGGCGTGCTTAACACATGCAAGTCGAACGGAAAGGCCC-T------------T--C----------------GGGGTACTCGAGTGGCGAACGGGTGAGTAACACGTGGGTGATCTGCCCTGCACTTT-GGGATAAGCCTGGGAAACTGGGTCTAATACCGAATATGACCA-T-GCGCCTCCTGGTG-T-GTGGTGGAAAG--CTT--T----T----GCGGTGTGGGATGGGCCCGCGGCCTATCAGCTTGTTGGTGGGGTAATGGCCTACCAAGGCGACGACGGGTAGCCGGCCTGAGAGGGTGACCGGCCACACTGGGACTGAGATACGGCCCAGACTCCTACGGGAGGCAGCAGTGGGGAATATTGCACAATGGGCGCAAGCCTGATGCAGCGACGCCGCGTGAGGGATGACGGCCTTCGGGTTGTAAACCTCTTTCAGCACAGACGAAGCGC----------------AAGTGACGGTATGTGCAGAAGAAGGACCGGCCAACTACGTGCCAGCAGCCGCGGTAATACGTAGGGTCCGAGCGTTGTCCGGAATTACTGGGCGTAAAGAGCTCGTAGGTGGTTTGTCGCGTTGTTCGTGAAAACTC-ACAACTCAATTGTGGGCGTGCGGGCGATACGGGCAGACTTGAGTACTGCAGGGGAGACTGGAATTCCTGGTGTAGCGGTGGAATGCGCAGATATCAGGAGGAACACCGGTGGCGAAGGCGGGTCTCTGGGCAGTAACTGACGCTGAGGAGCGAAAGCGTGGGGAGCGAACAGGATTAGATACCCTGGTAGTCCACGCCGTAAACGGTGGGTACTAGGTGTGGGTTTCCTTCCTT--GGGATCCGTGCCGTAGCTAACGCATTAAGTACCCCGCCTGGGGAGTACGGCCGCAAGGCTAAAACTCAAAGAAATTGACGGGGGCCCGCACAAGCGGCGGAGCATGTGGATTAATTCGATGCAACGCGAAGAACCTTACCTGGGTTTGACATGCACAGGACGCCGGCAGAGATGTCGGTTCCCTTGTGGCCTGTGTGCAGGTGGTGCATGGCTGTCGTCAGCTCGTGTCGTGAGATGTTGGGTTAAGTCCCGCAACGAGCGCAACCCTTGTCCTATGTTGCCAGCGGGTTATGCCGGGGACTCGTAGGAGACTGCCGGGGTCAACTCGGAGGAAGGTGGGGATGACGTCAAGTCATCATGCCCCTTATGTCCAGGGCTTCACACATGCTACAATGGCCGGTACAAAGGGCTGCGATGCCGTGAGGTGGAGCGAATCCTT-TCAAAGCCGGTCTCAGTTCGGATCGGGGTCTGCAACTCGACCCCGTGAAGTCGGAGTCGCTAGTAATCGCAGATCAGCAACGCTGCGGTGAATACGTTCCCGGGCCTTGTACACACCGCCCGTCACGTCATGAAAGTCGGTAACACCCGAAGCCGGTGGCCTAACCCCT-TGTGGGAGGGAGCCGTCGAAGGTGGGATCGGCGATTGGGACGAAGTCGTAACAAGGTAGCCGTACCGGAAGGTGCGGCTGGATCACCTCCTTTCTAA---->M._sp._UM_Kg1----------TGTTTGGAGAGTTTGATCCTGGCTCAGGACGAACGCTGGCGGCGTGCTTAACACATGCAAGTCGAACGGAAAGGCCCCT------------T--C---------------GGGGGTACTCGAGTGGCGAACGGGTGAGTAACACGTGGGTGATCTGCCCTGCACTCT-GGGATAAGCCTGGGAAACTGGGTCTAATACCGGATAGGACCG-C-GCGCTTCATGGTG-T-GTGGTGGAAAG--CTT--T----T----GCGGTGTGGGATGGGCCCGCGGCCTATCAGCTTGTTGGTGGGGTAATGGCCTACCAAGGCGACGACGGGTAGCCGGCCTGAGAGGGTGACCGGCCACACTGGGACTGAGATACGGCCCAGACTCCTACGGGAGGCAGCAGTGGGGAATATTGCACAATGGGCGCAAGCCTGATGCAGCGACGCCGCGTGGGGGATGACGGCCTTCGGGTTGTAAACCTCTTTCAGTATCGGCGAAGCTCCGT-GGTTTTCTGC-GGGGTGACGGTAGGTACAGAAGAAGCACCGGCCAACTACGTGCCAGCAGCCGCGGTAATACGTAGGGTGCGAGCGTTGTCCGGAATTACTGGGCGTAAAGAGCTCGTAGGTGGTTTGTCACGTTGTTCGTGAAAACTC-ACAGCTTAACTGTGGGCGTGCGGGCGATACGGGCAGACTAGAGTACTGTAGGGGAGACTGGAATTCCTGGTGTAGCGGTGGAATGCGCAGATATCAGGAGGAACACCGGTGGCGAAGGCGGGTCTCTGGGCAGTAACTGACGCTGAGGAGCGAAAGCGTGGGGAGCGAACAGGATTAGATACCCTGGTAGTCCACGCCGTAAACGGTGGGTACTAGGTGTGGGTTTCCTTCCTTTAGGGATCCGTGCCGTAGCTAACGCATTAAGTACCCCGCCTGGGGAGTACGGCCGCAAGGCTAAAACTCAAAGGAATTGACGGGGGCCCGCACAAGCGGCGGAGCATGTGGATTAATTCGATGCAACGCGAAGAACCTTACCTGGGTTTGACATGCACAGGACGCCGGTAGAGATATCGGTTCCCTTGTGGCCTGTGTGCAGGTGGTGCATGGCTGTCGTCAGCTCGTGTCGTGAGATGTTGGGTTAAGTCCCGCAACGAGCGCAACCCTTGTCTCATGTTGCCAGCACGTTATGGTGGGGACTCGTGAGAGACTGCCGGGGTCAACTCGGAGGAAGGTGGGGATGACGTCAAGTCATCATGCCCCTTATGTCCAGGGCTTCACACATGCTACAATGGCCGGTACAAAGGGCTGCGATCCCGTGAGGGTTAGCGAATCCTT--TAAAGCCGGTCTCAGTTCGGATTGGGGTCTGCAACTCGACCCCATGAAGTCGGAGTCGCTAGTAATCGCAGATCAGCAACGCTGCGGTGAATACGTTCCCGGGCCTTGTACACACCGCCCGTCACGTCATGAAAGTCGGTAACACCCGAAGCCGGTGGCCTAACCCTT---GTGGAGGGAGCCGTCGAAGGTGGGATCGGCGATTGGGACGAAGTCGTAACAAGGTAGCCGTACCGGAAGGTGCGGCTGGATCACCTCCTTT-------->M._sp._URHB0044---------TTGTTTGGAGAGTTTGATTCTGGCTCAGGACGAACGCTGGCGGCGTGCTTAACACATGCAAGTCGAACGGTAAGGCCC-T------------T--C----------------GGGGTACACGAGTGGCGAACGGGTGAGTAACACGTGGGTGATCTGCCCTGCACTTT-GGGATAAGCCTGGGAAACTGGGTCTAATACCGAATACACC----------------------------------------------------------------------------------------------------------------------------------------------------------------------------------------------------------------------------------------------------------------------------------------------------------------------------------------------------------------------------------------------------------------------------------------------------------------------------------------------------------------------------------------------------------------------------------------------------------------------------------------------------------------------------------------------------------------------------------------------------------------------------------------------------------------------------------------------------------------------------------------------------------------------------------------------------------------------------------------------------------------------------------------------------------------------------------------------------------------------------------------------------------------------------------------------------------------------------------------------------------------------------------------------------------------------------------------------------------------------------------------------------------------------------------TCT-------->M._sp._VKM_Ac-1817DTGGGAGTTTTTGTTTGGAGAGTTTGATCCTGGCTCAGGACGAACGCTGGCGGCGTGCTTAACACATGCAAGTCGAACGGAAAGGCCC-T------------T--C----------------GGGGTACTCGAGTGGCGAACGGGTGAGTAACACGTGGGTGATCTGCCCTGCACTTT-GGGATAAGCCTGGGAAACTGGGTCTAATACCGAATATGACCA-C-GCGCTTCATGGTG-T-GTGGTGGAAAG--CTT--T----T----GCGGTGTGGGATGGGCCCGCGGCCTATCAGCTTGTTGGTGGGGTAATGGCCTACCAAGGCGACGACGGGTAGCCGGCCTGAGAGGGTGACCGGCCACACTGGGACTGAGATACGGCCCAGACTCCTACGGGAGGCAGCAGTGGGGAATATTGCACAATGGGCGCAAGCCTGATGCAGCGACGCCGCGTGAGGGATGACGGCCTTCGGGTTGTAAACCTCTTTCAATAGGGACGAAGCGC----------------AAGTGACGGTACCTATAGAAGAAGGACCGGCCAACTACGTGCCAGCAGCCGCGGTAATACGTAGGGTCCGAGCGTTGTCCGGAATTACTGGGCGTAAAGAGCTCGTAGGTGGTTTGTCGCGTTGTTCGTGAAAACTC-ACAGCTTAACTGTGGGCGTGCGGGCGATACGGGCAGACTAGAGTACTGCAGGGGAGACTGGAATTCCTGGTGTAGCGGTGGAATGCGCAGATATCAGGAGGAACACCGGTGGCGAAGGCGGGTCTCTGGGCAGTAACTGACGCTGAGGAGCGAAAGCGTGGGGAGCGAACAGGATTAGATACCCTGGTAGTCCACGCCGTAAACGGTGGGTACTAGGTGTGGGTTTCCTTCCTT--GGGATCCGTGCCGTAGCTAACGCATTAAGTACCCCGCCTGGGGAGTACGGCCGCAAGGCTAAAACTCAAAGGAATTGACGGGGGCCCGCACAAGCGGCGGAGCATGTGGATTAATTCGATGCAACGCGAAGAACCTTACCTGGGTTTGACATGCACAGGACGACTGCAGAGATGTGGTTTCCCTTGTGGCCTGTGTGCAGGTGGTGCATGGCTGTCGTCAGCTCGTGTCGTGAGATGTTGGGTTAAGTCCCGCAACGAGCGCAACCCTTGACTCATGTTGCCAGCACGTTATGGTGGGGACTCGTGAGAGACTGCCGGGGTCAACTCGGAGGAAGGTGGGGATGACGTCAAGTCATCATGCCCCTTATGTCCAGGGCTTCACACATGCTACAATGGCCGGTACAAAGGGCTGCGATGCCGTGAGGTGGAGCGAATCCTT-TCAAAGCCGGTCTCAGTTCGGATCGGGGTCTGCAACTCGACCCCGTGAAGTCGGAGTCGCTAGTAATCGCAGATCAGCAACGCTGCGGTGAATACGTTCCCGGGCCTTGTACACACCGCCCGTCACGTCATGAAAGTCGGTAACACCCGAAGCCGGTGGCCTAACCCTT---GTGGAGGGAGCCGTCGAAGGTGGGATCGGCGATTGGGACGAAGTCGTAACAAGGTAGCCGTACCGGAAGGTGCGGCTGGATCACCTCCTTTCTAA---->M._sp._YCRL4----------TGTTTGGAGAGTTTGATTCTGGCTCAGGACGAACGCTGGCGGCGTGCTTAACACATGCAAGTCGAACGGAAAGGCCC-T------------T--C----------------GGGGTACTCGAGTGGCGAACGGGTGAGTAACACGTGGGTGATCTGCCCTGCACTTT-GGGATAAGCCTGGGAAACTGGGTCTAATACCGAATATGACCA-T-GCACTTCCTGGTG-T-GTGGTGGAAAG--CTT--T----T----GCGGTGTGGGATGGGCCCGCGGCCTATCAGCTTGTTGGTGGGGTAATGGCCTACCAAGGCGACGACGGGTAGCCGGCCTGAGAGGGTGACCGGCCACACTGGGACTGAGATACGGCCCAGACTCCTACGGGAGGCAGCAGTGGGGAATATTGCACAATGGGCGCAAGCCTGATGCAGCGACGCCGCGTGAGGGATGACGGCCTTCGGGTTGTAAACCTCTTTCAGCACAGACGAAGCGC----------------AAGTGACGGTATGTGCAGAAGAAGGACCGGCCAACTACGTGCCAGCAGCCGCGGTAATACGTAGGGTCCGAGCGTTGTCCGGAATTACTGGGCGTAAAGAGCTCGTAGGTGGTTTGTCGCGTTGTTCGTGAAAACTC-ACAGCTTAACTGTGGGCGTGCGGGCGATACGGGCAGACTTGAGTACTGCAGGGGAGACTGGAATTCCTGGTGTAGCGGTGGAATGCGCAGATATCAGGAGGAACACCGGTGGCGAAGGCGGGTCTCTGGGCAGTAACTGACGCTGAGGAGCGAAAGCGTGGGGAGCGAACAGGATTAGATACCCTGGTAGTCCACGCCGTAAACGGTGGGTACTAGGTGTGGGTTTCCTTCCTT--GGGATCCGTGCCGTAGCTAACGCATTAAGTACCCCGCCTGGGGAGTACGGCCGCAAGGCTAAAACTCAAAGAAATTGACGGGGGCCCGCACAAGCGGCGGAGCATGTGGATTAATTCGATGCAACGCGAAGAACCTTACCTGGGTTTGACATGCACAGGACGCCAGTAGAGATATTGGTTCCCTTGTGGCCTGTGTGCAGGTGGTGCATGGCTGTCGTCAGCTCGTGTCGTGAGATGTTGGGTTAAGTCCCGCAACGAGCGCAACCCTTGTCCTATGTTGCCAGCGGGTTATGCCGGGGACTCGTAGGAGACTGCCGGGGTCAACTCGGAGGAAGGTGGGGATGACGTCAAGTCATCATGCCCCTTATGTCCAGGGCTTCACACATGCTACAATGGCCGGTACAAAGGGCTGCGATGCCGTGAGGTGGAGCGAATCCTT-TCAAAGCCGGTCTCAGTTCGGATCGGGGTCTGCAACTCGACCCCGTGAAGTCGGAGTCGCTAGTAATCGCAGATCAGCAACGCTGCGGTGAATACGTTCCCGGGCCTTGTACACACCGCCCGTCACGTCATGAAAGTCGGTAACACCCGAAGCCGGTGGCCTAACCCTT---GTGGAGGGAGCCGTCGAAGGTGGGATCGGCGATTGGGACGAAGTCGTAACAAGGTAGCCGTACCGGAAGGTGCGGCTGGATCACCTCCTTT-------->M._stomatepiae---------TTGTTTGGAGAGTTTGATCCTGGCTCAGGACGAACGCTGGCGGCGTGCTTAACACATGCAAGTCGAACGGAAAGGCCCCT------------T--C---------------GGGGGTACTCGAGTGGCGAACGGGTGAGTAACACGTGGGTAATCTGCCCTGCACTTC-GGGATAAGCCTGGGAAACTGGGTCTAATACCGGATATGACCA-C-GAGGCGCATGCCT-T-GTGGTGGAAAG--CTT--T----T----GCGGTGTGGGATGGGCCCGCGGCCTATCAGCTTGTTGGTGGGGTGATGGCCTACCAAGGCGACGACGGGTAGCCGGCCTGAGAGGGTGTCCGGCCACACTGGGACTGAGATACGGCCCAGACTCCTACGGGAGGCAGCAGTGGGGAATATTGCACAATGGGCGCAAGCCTGATGCAGCGACGCCGCGTGGGGGATGACGGCCTTCGGGTTGTAAACCTCTTTCAGCAGGGACGAAGCGC----------------AAGTGACGGTACCTGCAGAAGAAGCACCGGCCAACTACGTGCCAGCAGCCGCGGTAATACGTAGGGTGCGAGCGTTGTCCGGAATTACTGGGCGTAAAGAGCTCGTAGGTGGTTTGTCGCGTTGTTCGTGAAAACCG-GGGGCTTAACCCTCGGCGTGCGGGCGATACGGGCAGACTGGAGTACTGCAGGGGAGACTGGAATTCCTGGTGTAGCGGTGGAATGCGCAGATATCAGGAGGAACACCGGTGGCGAAGGCGGGTCTCTGGGCAGTAACTGACGCTGAGGAGCGAAAGCGTGGGGAGCGAACAGGATTAGATACCCTGGTAGTCCACGCCGTAAACGGTGGGTACTAGGTGTGGGTTTCCTTCCTT--GGAATCCGTGCCGTAGCTAACGCATTAAGTACCCCGCCTGGGGAGTACGGCCGCAAGGCTAAAACTCAAAGGAATTGACGGGGGCCCGCACAAGCGGCGGAGCATGTGGATTAATTCGATGCAACGCGAAGAACCTTACCTGGGTTTGACATGCACAGGACGCCGGCAGAGATGTCGGTTCCCTTGTGGCCTGTGTGCAGGTGGTGCATGGCTGTCGTCAGCTCGTGTCGTGAGATGTTGGGTTAAGTCCCGCAACGAGCGCAACCCTTGTCTCATGTTGCCAGCACGTAATGGTGGGGACTCGTGAGAGACTGCCGGGGTCAACTCGGAGGAAGGTGGGGATGACGTCAAGTCATCATGCCCCTTATGTCCAGGGCTTCACACATGCTACAATGGGCGGTACAAAGGGCTGCGATGCCGTAAGGTTAAGCGAATCCTT-ATAAAGCCGGTCTCAGTTCGGATCGGGGTCTGCAACTCGACCCCGTGAAGTCGGAGTCGCTAGTAATCGCAGATCAGCAACGCTGCGGTGAATACGTTCCCGGGCCTTGTACACACCGCCCGTCACGTCATGAAAGTCGGTAACACCCGAAGCCAGTGGCCTAACCTT----TTGGAGGGAGCTGTCGAAGGTGGGATCGGCGATTGGGACGAAGTCGTAACAAGGTAGCCGTACCGGAAGGTGCGGCTGGATCACCTCCTTTCTAA---->M._szulgai----------TGTTTGGAGAGTTTGATCCTGGCTCAGGACGAACGCTGGCGGCGTGCTTAACACATGCAAGTCGAACGGAAAGGCCCCT------------T--C---------------GGGGGTACTCGAGTGGCGAACGGGTGAGTAACACGTGGGTAATCTGCCCTGCACTTC-GGGATAAGCCTGGGAAACTGGGTCTAATACCGGATAGGACCC-C-GAGGCGCATGCCT-T-GGGGTGGAAAG--CTT--T----T----GCGGTGTGGGATGGGCCCGCGGCCTATCAGCTTGTTGGTGGGGTGACGGCCTACCAAGGCGACGACGGGTAGCCGGCCTGAGAGGGTGTCCGGCCACACTGGGACTGAGATACGGCCCAGACTCCTACGGGAGGCAGCAGTGGGGAATATTGCACAATGGGCGCAAGCCTGATGCAGCGACGCCGCGTGGGGGATGACGGCCTTCGGGTTGTAAACCTCTTTCACCATCGACGAAGGTCCGG-GTTT--TCTC-GGATTGACGGTAGGTGGAGAAGAAGCACCGGCCAACTACGTGCCAGCAGCCGCGGTAATACGTAGGGTGCGAGCGTTGTCCGGAATTACTGGGCGTAAAGAGCTCGTAGGTGGTTTGTCGCGTTGTTCGTGAAATCTC-ACGGCTTAACTGTGAGCGTGCGGGCGATACGGGCAGACTGGAGTACTGCAGGGGAGACTGGAATTCCTGGTGTAGCGGTGGAATGCGCAGATATCAGGAGGAACACCGGTGGCGAAGGCGGGTCTCTGGGCAGTAACTGACGCTGAGGAGCGAAAGCGTGGGGAGCGAACAGGATTAGATACCCTGGTAGTCCACGCCGTAAACGGTGGGTACTAGGTGTGGGTTTCCTTCCTT--GGGATCCGTGCCGTAGCTAACGCATTAAGTACCCCGCCTGGGGAGTACGGCCGCAAGGCTAAAACTCAAAGGAATTGACGGGGGCCCGCACAAGCGGCGGAGCATGTGGATTAATTCGATGCAACGCGAAGAACCTTACCTGGGTTTGACATGCACAGGACGCGTCTAGAGATAGGCGTTCCCTTGTGGCCTGTGTGCAGGTGGTGCATGGCTGTCGTCAGCTCGTGTCGTGAGATGTTGGGTTAAGTCCCGCAACGAGCGCAACCCTTGTCTCATGTTGCCAGCGGGTAATGCCGGGGACTCGTGAGAGACTGCCGGGGTCAACTCGGAGGAAGGTGGGGATGACGTCAAGTCATCATGCCCCTTATGTCCAGGGCTTCACACATGCTACAATGGCCGGTACAAAGGGCTGCGATGCCGCGAGGTTAAGCGAATCCTT-TTAAAGCCGGTCTCAGTTCGGATCGGGGTCTGCAACTCGACCCCGTGAAGTCGGAGTCGCTAGTAATCGCAGATCAGCAACGCTGCGGTGAATACGTTCCCGGGCCTTGTACACACCGCCCGTCACGTCATGAAAGTCGGTAACACCCGAAGCCAGTGGCCTAACCCT----TGGGAGGGAGCTGTCGAAGGTGGGATCGGCGATTGGGACGAAGTCGTAACAAGGTAGCCGTACCGGAAGGTGCGGCTGGATCACCTCCTTT-------->M._timonense---------TTGTTTGGAGAGTTTGATCCTGGCTCAGGACGAACGCTGGCGGCGTGCTTAACACATGCAAGTCGAACGGAAAGGCCTCT------------T--C---------------GGAGGTACTCGAGTGGCGAACGGGTGAGTAACACGTGGGCAATCTGCCCTGCACTTC-GGGATAAGCCTGGGAAACTGGGTCTAATACCGGATAGGACCT-C-AAGACGCATGTCT-T-CTGGTGGAAAG--CTT--T----T----GCGGTGTGGGATGGGCCCGCGGCCTATCAGCTTGTTGGTGGGGTGACGGCCTACCAAGGCGACGACGGGTAGCCGGCCTGAGAGGGTGTCCGGCCACACTGGGACTGAGATACGGCCCAGACTCCTACGGGAGGCAGCAGTGGGGAATATTGCACAATGGGCGCAAGCCTGATGCAGCGACGCCGCGTGGGGGATGACGGCCTTCGGGTTGTAAACCTCTTTCACCATCGACGAAGGTCCGG-GTTT--TCTC-GGATTGACGGTAGGTGGAGAAGAAGCACCGGCCAACTACGTGCCAGCAGCCGCGGTAATACGTAGGGTGCGAGCGTTGTCCGGAATTACTGGGCGTAAAGAGCTCGTAGGTGGTTTGTCGCGTTGTTCGTGAAATCTC-ACGGCTTAACTGTGAGCGTGCGGGCGATACGGGCAGACTAGAGTACTGCAGGGGAGACTGGAATTCCTGGTGTAGCGGTGGAATGCGCAGATATCAGGAGGAACACCGGTGGCGAAGGCGGGTCTCTGGGCAGTAACTGACGCTGAGGAGCGAAAGCGTGGGGAGCGAACAGGAGTAGATACCCTGGTAGTCCACGCCGTAAACGGTGGGTACTAGGTGTGGGTTTCCTTCCTT--GGGATCCGTGCCGTAGCTAACGCATTAAGTACCCCGCCTGGGGAGTACGGCCGCAAGGCTAAAACTCAAAGGAATTGACGGGGGCCCGCACAAGCGGCGGAGCATGTGGATTAATTCGATGCAACGCGAAGAACCTTACCTGGGTTTGACATGCACAGGACGCGTCTAGAGATAGGCGTTCCCTTGTGGCCTGTGTGCAGGTGGTGCATGGCTGTCGTCAGCTCGTGTCGTGAGATGTTGGGTTAAGTCCCGCAACGAGCGCAACCCTTGTCTCATGTTGCCAGCGGGTAATGCCGGGGACTCGTGAGAGACTGCCGGGGTCAACTCGGAGGAAGGTGGGGATGACGTCAAGTCATCATGCCCCTTATGTCCAGGGCTTCACACATGCTACAATGGCCGGTACAAAGGGCTGCGATGCCGTAAGGTTAAGCGAATCCTT-TTAAAGCCGGTCTCAGTTCGGATTGGGGTCTGCAACTCGACCCCATGAAGTCGGAGTCGCTAGTAATCGCAGATCAGCAACGCTGCGGTGAATACGTTCCCGGGCCTTGTACACACCGCCCGTCACGTCATGAAAGTCGGTAACACCCGAAGCCAGTGGCCTAACCCTT--TTGGGAGGGAGCTGTCGAAGGTGGGATCGGCGATTGGGACGAAGTCGTAACAAGGTAGCCGTACCGGAAGGTGCGGCTGGATCACCTCCTTTCTAAGGAG>M._triplex----------TGTTTGGAGAGTTTGATCCTGGCTCAGGACGAACGCTGGCGGCGTGCTTAACACATGCAAGTCGAACGGAAAGGCCTCT------------T--C---------------GGAGGTACTCGAGTGGCGAACGGGTGAGTAACACGTGGGTAATCTGCCCTGCACTTC-GGGATAAGCCTGGGAAACTGGGTCTAATACCGGATATGACCA-C-GAGACGCATGTCC-T-GTGGTGGAAAG--CTT--T----T----GCGGTGTGGGATGGGCCCGCGGCCTATCAGCTTGTTGGTGGGGTGACGGCCTACCAAGGCGACGACGGGTAGCCGGCCTGAGAGGGTGTCCGGCCACACTGGGACTGAGATACGGCCCAGACTCCTACGGGAGGCAGCAGTGGGGAATATTGCACAATGGGCGCAAGCCTGATGCAGCGACGCCGCGTGGGGGATGACGGCCTTCGGGTTGTAAACCTCTTTCAGCAGGGACGAAGCGC----------------AAGTGACGGTACCTGCAGAAGAAGCACCGGCCAACTACGTGCCAGCAGCCGCGGTAATACGTAGGGTGCGAGCGTTGTCCGGAATTACTGGGCGTAAAGAGCTCGTAGGTGGTTTGTCGCGTTGTTCGTGAAAACCG-GGGGCTTAACCCTCGGCGTGCGGGCGATACGGGCAGACTGGAGTACTGCAGGGGAGACTGGAATTCCTGGTGTAGCGGTGGAATGCGCAGATATCAGGAGGAACACCGGTGGCGAAGGCGGGTCTCTGGGCAGTAACTGACGCTGAGGAGCGAAAGCGTGGGGAGCGAACAGGATTAGATACCCTGGTAGTCCACGCCGTAAACGGTGGGTACTAGGTGTGGGTTTCCTTCCTT--GGAATCCGTGCCGTAGCTAACGCATTAAGTACCCCGCCTGGGGAGTACGGCCGCAAGGCTAAAACTCAAAGGAATTGACGGGGGCCCGCACAAGCGGCGGAGCATGTGGATTAATTCGATGCAACGCGAAGAACCTTACCTGGGTTTGACATGCACAGGACGCCGGCAGAGATGTCGGTTCCCTTGTGGCCTGTGTGCAGGTGGTGCATGGCTGTCGTCAGCTCGTGTCGTGAGATGTTGGGTTAAGTCCCGCAACGAGCGCAACCCTTGTCTCATGTTGCCAGCGGGTAATGCCGGGGACTCGTGAGAGACTGCCGGGGTCAACTCGGAGGAAGGTGGGGATGACGTCAAGTCATCATGCCCCTTATGTCCAGGGCTTCACACATGCTACAATGGCCGGTACAAAGGGCTGCGATGCCGTAAGGTTAAGCGAATCCTT-TTAAAGCCGGTCTCAGTTCGGATCGGGGTCTGCAACTCGACCCCGTGAAGTCGGAGTCGCTAGTAATCGCAGATCAGCAACGCTGCGGTGAATACGTTCCCGGGCCTTGTACACACCGCCCGTCACGTCATGAAAGTCGGTAACACCCGAAGCCAGTGGCCTAACCTT----TTGGAGGGAGCTGTCGAAGGTGGGATCGGCGATTGGGACGAAGTCGTAACAAGGTAGCCGTACCGGAAGGTGCGGCTGGATCACCTCCTTT-------->M._tuberculosisT-------TTTGTTTGGAGAGTTTGATCCTGGCTCAGGACGAACGCTGGCGGCGTGCTTAACACATGCAAGTCGAACGGAAAGGTCTCT------------T--C---------------GGAGATACTCGAGTGGCGAACGGGTGAGTAACACGTGGGTGATCTGCCCTGCACTTC-GGGATAAGCCTGGGAAACTGGGTCTAATACCGGATAGGACCA-C-GGGATGCATGTCT-T-GTGGTGGAAAGCGCTT--T----A----GCGGTGTGGGATGAGCCCGCGGCCTATCAGCTTGTTGGTGGGGTGACGGCCTACCAAGGCGACGACGGGTAGCCGGCCTGAGAGGGTGTCCGGCCACACTGGGACTGAGATACGGCCCAGACTCCTACGGGAGGCAGCAGTGGGGAATATTGCACAATGGGCGCAAGCCTGATGCAGCGACGCCGCGTGGGGGATGACGGCCTTCGGGTTGTAAACCTCTTTCACCATCGACGAAGGTCCGG-GTTC--TCTC-GGATTGACGGTAGGTGGAGAAGAAGCACCGGCCAACTACGTGCCAGCAGCCGCGGTAATACGTAGGGTGCGAGCGTTGTCCGGAATTACTGGGCGTAAAGAGCTCGTAGGTGGTTTGTCGCGTTGTTCGTGAAATCTC-ACGGCTTAACTGTGAGCGTGCGGGCGATACGGGCAGACTAGAGTACTGCAGGGGAGACTGGAATTCCTGGTGTAGCGGTGGAATGCGCAGATATCAGGAGGAACACCGGTGGCGAAGGCGGGTCTCTGGGCAGTAACTGACGCTGAGGAGCGAAAGCGTGGGGAGCGAACAGGATTAGATACCCTGGTAGTCCACGCCGTAAACGGTGGGTACTAGGTGTGGGTTTCCTTCCTT--GGGATCCGTGCCGTAGCTAACGCATTAAGTACCCCGCCTGGGGAGTACGGCCGCAAGGCTAAAACTCAAAGGAATTGACGGGGGCCCGCACAAGCGGCGGAGCATGTGGATTAATTCGATGCAACGCGAAGAACCTTACCTGGGTTTGACATGCACAGGACGCGTCTAGAGATAGGCGTTCCCTTGTGGCCTGTGTGCAGGTGGTGCATGGCTGTCGTCAGCTCGTGTCGTGAGATGTTGGGTTAAGTCCCGCAACGAGCGCAACCCTTGTCTCATGTTGCCAGCACGTAATGGTGGGGACTCGTGAGAGACTGCCGGGGTCAACTCGGAGGAAGGTGGGGATGACGTCAAGTCATCATGCCCCTTATGTCCAGGGCTTCACACATGCTACAATGGCCGGTACAAAGGGCTGCGATGCCGCGAGGTTAAGCGAATCCTT--AAAAGCCGGTCTCAGTTCGGATCGGGGTCTGCAACTCGACCCCGTGAAGTCGGAGTCGCTAGTAATCGCAGATCAGCAACGCTGCGGTGAATACGTTCCCGGGCCTTGTACACACCGCCCGTCACGTCATGAAAGTCGGTAACACCCGAAGCCAGTGGCCTAACCCT----CGGGAGGGAGCTGTCGAAGGTGGGATCGGCGATTGGGACGAAGTCGTAACAAGGTAGCCGTACCGGAAGGTGCGGCTGGATCACCTCCTTTCT------>M._ulceransT------TTTTGTTTGGAGAGTTTGATCCTGGCTCAGGACGAACGCTGGCGGCGTGCTTAACACATGCAAGTCGAACGGAAAGGTCTCT------------T--C---------------GGAGATACTCGAGTGGCGAACGGGTGAGTAACACGTGGGCGATCTGCCCTGCACTTC-GGGATAAGCCTGGGAAACTGGGTCTAATACCGGATAGGACCA-C-GGGATTCATGTCC-T-GTGGTGGAAAG--CTT--T----T----GCGGTGTGGGATGGGCCCGCGGCCTATCAGCTTGTTGGTGGGGTAACGGCCTACCAAGGCGACGACGGGTAGCCGGCCTGAGAGGGTGTCCGGCCACACTGGGACTGAGATACGGCCCAGACTCCTACGGGAGGCAGCAGTGGGGAATATTGCACAATGGGCGCAAGCCTGATGCAGCGACGCCGCGTGGGGGATGACGGCCTTCGGGTTGTAAACCTCTTTCACCATCGACGAAGGTTCGG-GTTT--TCTC-GGATTGACGGTAGGTGGAGAAGAAGCACCGGCCAACTACGTGCCAGCAGCCGCGGTAATACGTAGGGTGCGAGCGTTGTCCGGAATTACTGGGCGTAAAGAGCTCGTAGGTGGTTTGTCGCGTTGTTCGTGAAATCTC-ACGGCTTAACTGTGAGCGTGCGGGCGATACGGGCAGACTAGAGTACTGCAGGGGAGACTGGAATTCCTGGTGTAGCGGTGGAATGCGCAGATATCAGGAGGAACACCGGTGGCGAAGGCGGGTCTCTGGGCAGTAACTGACGCTGAGGAGCGAAAGCGTGGGGAGCGAACAGGATTAGATACCCTGGTAGTCCACGCCGTAAACGGTGGGTACTAGGTGTGGGTTTCCTTCCTT--GGGATCCGTGCCGTAGCTAACGCATTAAGTACCCCGCCTGGGGAGTACGGCCGCAAGGCTAAAACTCAAAGGAATTGACGGGGGCCCGCACAAGCGGCGGAGCATGTGGATTAATTCGATGCAACGCGAAGAACCTTACCTGGGTTTGACATGCACAGGACGCGTCTAGAGATAGGCGTTCCCTTGTGGCCTGTGTGCAGGTGGTGCATGGCTGTCGTCAGCTCGTGTCGTGAGATGTTGGGTTAAGTCCCGCAACGAGCGCAACCCTTGTCTCATGTTGCCAGCACGTAATGGTGGGGACTCGTGAGAGACTGCCGGGGTCAACTCGGAGGAAGGTGGGGATGACGTCAAGTCATCATGCCCCTTATGTCCAGGGCTTCACACATGCTACAATGGCCGGTGCAAAGGGCTGCGATGCCGCGAGGTTAAGCGAATCCTT--TAACGCCGGTCTCAGTTCGGATCGGGGTCTGCAACTCGACCCCGTGAAGTCGGAGTCGCTAGTAATCGCAGATCAGCAACGCTGCGGTGAATACGTTCCCGGGCCTTGTACACACCGCCCGTCACGTCATGAAAGTCGGTAACACCCGAAGCCAGTGGCCTAACCCTTTTTTGGGAGGGAGCTGTCGAAGGTGGGATCGGCGATTGGGACGAAGTCGTAACAAGGTAGCCGTACCGGAAGGTGCGGCTGGATCACCTCCTTTCTAAGGAG>M._xenopi---------TTGTTTGGAGAGTTTGATCCTGGCTCAGGACGAACGCTGGCGGCGTGCTTAACACATGCAAGTCGAACGGAAAGGCCCCT------------TTTT---------------TGGGGTGCTCGAGTGGCGAACGGGTGAGTAACACGTGGGTGACCTGCCCTGCACTTC-GGGATAAGCCTGGGAAACTGGGTCTAATACCGGATAGGACCA-T-TCTGCGCATGTGG-G-GTGGTGGAAAG--TGT--T----TGGTAGCGGTGTGGGATGGGCCCGCGGCCTATCAGCTTGTTGGTGGGGTGATGGCCTACCAAGGCGACGACGGGTAGCCGGCCTGAGAGGGTGTCCGGCCACACTGGGACTGAGATACGGCCCAGACTCCTACGGGAGGCAGCAGTGGGGAATATTGCACAATGGGCGCAAGCCTGATGCAGCGACGCCGCGTGGGGGATGACGGCCTTCGGGTTGTAAACCCCTTTCAGCCTCGACGAAGCTGCGG-GTTT--TCTC-GTGGTGACGGTAGGGGCAGAAGAAGCACCGGCCAACTACGTGCCAGCAGCCGCGGTAATACGTAGGGTGCAAGCGTTGTCCGGAATTACTGGGCGTAAAGAGCTCGTAGGCGGCTTGTCGCGTTGTTCGTGGAATGCC-ACAGCTTAACTGTGGGCGTGCGGGCGATACGGGCAGGCTGGAGTGCTGCAGGGGAGACTGGAATTCCTGGTGTAGCGGTGGAATGCGCAGATATCAGGAGGAACACCGGTGGCGAAGGCGGGTCTCTGGGCAGTAACTGACGCTGAGGAGCGAAAGCGTGGGGAGCGAACAGGATTAGATACCCTGGTAGTCCACGCCGTAAACGGTGGGTACTAGGTGTGGGTTCTTTCCTGA--AGGATCCGTGCCGTAGCTAACGCATTAAGTACCCCGCCTGGGGAGTACGGCCGCAAGGCTAAAACTCAAAGGAATTGACGGGGGCCCGCACAAGCGGCGGAGCATGTGGATTAATTCGATGCAACGCGAAGAACCTTACCTGGGTTTGACATGCACAGGACG---GTCGAGATACGGCTTCCCTTGTGGCCTGTGTGCAGGTGGTGCATGGCTGTCGTCAGCTCGTGTCGTGAGATGTTGGGTTAAGTCCCGCAACGAGCGCAACCCTTGTCCCATGTTGCCAGCACGTGATGGTGGGGACTCATGGGAGACTGCCGGGGTCAACTCGGAGGAAGGTGGGGATGACGTCAAGTCATCATGCCCCTTATGTCCAGGGCTTCACACATGCTACAATGGCCGGTACAAAGGGCTGCGATGCCGTGAGGTTAAGCGAATCCTT-GGAAAGCCGGTCTCAGTTCGGATCGGGGTCTGCAACTCGACCCCGTGAAGTCGGAGTCGCTAGTAATCGCAGATCAGCAATGCTGCGGTGAATACGTTCCCGGGCCTTGTACACACCGCCCGTCACGTCATGAAAGTCGGTAACACCCGAAGCCGCTGGCCTAACCCCGTTTGGGGAGGGAGGCGTCGAAGGTGGGATCGGCGATTGGGACGAAGTCGTAACAAGGTAGCCGTACCGGAAGGTGCGGCTGGATCACCTCCTTTCT-A----
