# Supplementary material for: The mevalonate pathway of isoprenoid biosynthesis supports metabolic flexibility in Mycobacterium marinum
Source: J Bacteriol. 2025 Oct 30;207(11):e00287-25. doi: 10.1128/jb.00287-25 (PMC12632256; doi:10.1128/jb.00287-25)
Supplement: File S2 — HmgR alignment across species. [file jb.00287-25-s0002.rtf]

>Actinoplanes_sp._A40644 hmgR, GenBank: BAD07379.1MSD--------------------------------------------------------------------------------T-TLTASVPLRWVGPLRITGNVGDIETEVPLATYESPLWPSVGRGAKISRLVGPGIVTTLVDERMTRSVLV-RAEDAQTAYMAALEVDARLDELREIVRTC-----GRFVE--LIGFHHE---ITAHLLYLRFDFTTGDASGHNMATLAADALLAHILKTI-----P-GISY-GSISGNYCTDKKATAVNGIL--------GRGKNVITELTIPRDIVRDNLHTTAAAIAELNVQKNLIGTLLAGGIRSANAHYANMLLGIYLATGQDAANIIEGSQGV-----------TV-AEDRD-GDLYFSCTLPNLILGTVGNGKGL-DFVEENLARLGCREAREPGENARRLAVITAATVLCGELSLLAAQTNP-GELMQAHVRLERD----------NTTAKI----------------------------------GE>Corynebacterium_striatum GenBank: PIS61883.1MLPLG----------------------KDFRH----LS----REEKLNCLVEYG-WLNKENQDM-LLTHPLIPEEIANSLIENV-IGQGALPVGLLPEIIVDD----KSYVVPMMVEEPSVVAAASYGAKLVNETG-GFHVIASERLMIGQIVFDNVKDTQRLSQNIKNLE---PKIKQIADESYPSIIKRGGGYRRVEIDTF---PEAQLVSLKVFVDTKDAMGANMLNTILEGISAYLKTEI-----EDVDIL-MSILSNHATASVVKVQGEIDVQSLSKDGRDGAKVAHRMER------------ASVLAQVDI----------HRAATHNKGVMNGIHAVVLATGNDTRGAEASAHAYAS-KDGQYRGIATWQYDASRQKLVGTIEVP-MTLATVGGGTRVLPIARAALDLMKV-------DSAQELGHVVAAVGLAQNFAACRALVSE-G-IQKGHMSLQYK----------SLAIVVGAQGDEITKVAELLKEKPSANAAMAEQILNQMREAH>Listeria_monocytogenes hmgR, GenBank: KHS63588.1MNAFD-----------------------KFYK----KT----VEERHAILAEYA-DLNEEEQAF-LASTGALSFDKANHMIENT-IGIYSLPLGLGMNMLLND----KRYVVPMAMEEPSVVAAQSAGAKLIAQNG-GITGSATKRKMIGQIELISVSDIQAAKENIIANE---EQLIAIANQAHPSLQKRGGGAVKIQVRTAQTANDETLFIVHLLVDTQEAMGANMVNTMVETLAPELE-ML-----TNGTAN-MRILSNLVDEATATAVCRINPESLATKTQSGEWVRDRIIA------------AYEFADADI----------YRAATHNKGIMNGIDAVIMAFGNDWRAVEAASHAYAA-RTGSYKPMSKWSKDAD-GYLVGELTLP-MPVAFVGGSIAIHPIASLSKKIARV-------ESAKELAMLVCAVGLTQNLAALKALVTE-G-IQRGHMSLQAK----------SLAMTAGAEADEIEIVATFLQESKQLNVVAAKEFIAKLRSEK>Mycobacterium_marinum hmgR, GenBank: ACC41640.1MTT-------------------------------------------------------------------------------QQEQLSVHVPLRWVGPIKISGSEGQFSAHAPLATYESPVWPSVGRGAKLSRLTENGIVTTLIDEKMTRSILLI-APDAAVANNTARQLQTEFAELAAVVGQT-----SRFAE--LIGMHPQ---ITANLLFIRFEFRTGDASGHNMATLAADHLIAHILTRY-----P-QMEY-GSISGNMCCDKKPTAINGIL--------GRGKNVVTEIVIPRDLVEQHLHTSAAAITALNIRKNLIGTLQAGTIRSANAHYANMLLAFYLATGQDAANIIAGSQGV-----------TH-AEDRD-GDLYFSCTIPNLIVGTVGNGKDL-DFVTDNLAQLGCREQRPPGANARRLAVICAATVLCGELSLMAAQTNPGE-LMRAHTTYERK------------------------------------------PS------TA>Nocardia_brasiliensis hmgR, GenBank: AFU04600.1MTE-------------------------------------------------------------------------------DT-FSAATVPMQWVGPLKISGNVAEGLVEVPLATYESPLWPSVGRGAKISTLCERGIVATLVDERMTRSVLLE-ADDAGTALAAARQLTAELPQLRRIVGEC-----SRFAD--LIDLHHQ---IVGNLLFLRFEFTTGDASGHNMVTLAADTLLGHIVNTV-----P-GVRY-GSVSGNFCTDKKASAVNGIL--------GRGKNVVAEILIPREIVAQRLHTTAAQVADLNVRKNLIGTTIAGGIRTANAHYANMLLGVYLATGQDAANIVEGSQGI-----------TH-VEDRD-GDLYFSCALPNLIVGTVGNGKGL-DFVEANLARMGCRESREVGGNARRLAVLTAAAVLCGELSLLAAQTNPGE-LMRTHIRFERS------------------------------------------AS-GEGT-GQ>Pseudomonas_mevalonii hmgR, UniProtKB/Swiss-Prot: P13702.1MSLDS--------------------RLPAFRN----LS----PAARLDHIGQLL-GLSHDDVSL-LANAGALPMDIANGMIENV-IGTFELPYAVASNFQING----RDVLVPLVVEEPSIVAAASYMAKLARANG-GFTTSSSAPLMHAQVQIVGIQDPLNARLSLLRRK---DEIIELANRKDQLLNSLGGGCRDIEVHTFADTPRGPMLVAHLIVDVRDAMGANTVNTMAEAVAPLME-AI-----TGGQVR-LRILSNLADLRLARAQVRITPQQLETAEFSGEAVIEGILD------------AYAFAAVDP----------YRAATHNKGIMNGIDPLIVATGNDWRAVEAGAHAYAC-RSGHYGSLTTWEKDNN-GHLVGTLEMP-MPVGLVGGATKTHPLAQLSLRILGV-------KTAQALAEIAVAVGLAQNLGAMRALATE-G-IQRGHMALHAR----------NIAVVAGARGDEVDWVARQLVEYHDVRADRAVALLKQKR-GQ>Rhodococcus_erythropolis hmgR, GenBank: RGP46201.1MSSTSSRSTNS--------------RIADFRS----YD----VAARRAEIVRQT-DLTEADIAV-YDAADGLTIDQADRMVENV-LGVIGIPVGVATNFTING----TDYLIPLATEEPSVVAAASNAARIARGLG-GFHVSSTQPIMQAQIQLVDVVDPAAARIRLLEAR---EEIIALANEQDPKLVSVGGGVKDISVRIVS-SDKAAYVVLHLHVDVRDAMGANAVNTMAEAIADRVA-EI-----GGGHVV-LRILTNKADLRLTRVRAVFDAEL-----IGGAEVVDNLIH------------AARLAELDP----------YRAATHNKGIMNGISAVVLATGNDTRAVEAGCHSHAVNADGIYSSLSHFEKNAD-GNIVGTLELP-MPVGLVGGATKVHPVAQAAIKMLGV-------ESAEELAGIILAVGLAQNLAAVRVLASE-G-VQRGHMGLHAR----------NIAATAGAQKSEIDAVVARLIADKSIRVEHAEKVLAEIRGGN>Staphylococcus_aureus hmgR, GenBank: OHS84849.1MQNLD----------------------KNFRH----LS----RKEKLQQLVDKQ-WLSEEQFDI-LLNHPLIDEEVANSLIENV-IAQGALPVGLLPNIIVDD----KAYVVPMMVEEPSVVAAASYGAKLVNQTG-GFKTVSSERIMIGQIVFDGVDDTEKLSADIKALE---KQIHKIADEAYPSIKARGGGYQRIAIDTF---PEQQLLSLKVFVDTKDAMGANMLNTILEAITAFLKNEF-----PQSDIL-MSILSNHATASVVKVQGEIDVKDLARGERTGEEVAKRMER------------ASVLAQVDI----------HRAATHNKGVMNGIHAVVLATGNDTRGAEASAHAYAS-RDGQYRGIATWRYDQDRQRLIGTIEVP-MTLAIVGGGTKVLPIAKASLELLNV-------ESAQELGHVVAAVGLAQNFAACRALVSE-G-IQQGHMSLQYK----------SLAIVVGAKGDEIAKVAEALKKEPRANTQAAEHILQEIR-QQ>Streptococcus_pneumoniae hmgR, GenBank: OBX46788.1MKISW----------------------NGFSK----KS----YQERLELLKAQA-LLSPERQAS-LEKDEQMSVTVADQLSENV-VGTFSLPYSLVPEVLVNG----QEYTVPYVTEEPSVVAAASYASKIIKRAG-GFTAQVHQRQMIGQVALYQVANPKLAQEKIASKK---AELLELANQAYPSIVKRGGGARDLHVEQIK--GEPDFLVVYIHVDTQEAMGANMLNTMLEALKPVLE-EL-----SQGQSL-MGILSNYATDSLVTASCRIAFRYLSRQKDQGREIAEKIAL------------ASQFAQADP----------YRAATHNKGIFNGIDAILIATGNDWRAIEAGAHAFAS-RDGRYQGLSCWTLDLEREELVGEMTLP-MPVATKGGSIGLNPRVALSHDLLGN-------PSARELAQIIVSIGLAQNFAALKALVST-G-IQQGHMKLQAK----------SLALLAGASESEVAPLVERLISDKTFNLETAQRYLENLR--S>Streptomyces_rutgerensis hmgR, GenBank: GFH69097.1MNSL---------------------------------------------------------------QHTRLDAGKLTGNIEGL-VGAVEIPIGVAGPLLFCGTNVRGERYAPLATTEGALVSSATRGALAVTMAG-GVSTHAVSQAMTRAPVFA-FSRLADASRFAATVPQHIGDLRTAVRQV-----SSHAV--LVSIEPV---ILGRAVHLRFRYTTGDAAGQNMTTVCTWHACQWILRQSHLVTDD-ALEF-FLVEGNSSGDKKASALAMAE--------GRGIRVAADCVLPTGVIERVLRTTPDELVRGYQ-HIAAGAVH-SAMLGSNANSANVVAAIFTATGQDIACVHESGMGH-----------FV-LERTR-DGVHASMTLPGLALGTVGGGTHL-PTQHEMLALMGCTG----PGSAVRLAEIIAGFALALDLSTVSAAVSD-D-FASAHERLGRNRLPHQSDTGRHVTPSAGAEGP-VTEAAGPLSLPPDVHTQEREAS-AEGG-AR>Vibrio_cholerae hmgR, GenBank: GIA94837.1MPKLALQPLYSPDVATLAPDRLIDQLAPRFDRPSVRLTPSPALTEQ-QVMKRWQKLAAPEAQALLLDEQTERTMQAYQKNIEYF-IGTVKLPVGIAGPLRVNGSHAQGDYLVPLATTEAALVASYHRGSQLITAAG-GASALLLNEGVTRTPVFA-FLSLAQAGQFVGWVTSQFEQMKEVAQST-----TAHGKLKDIQ--VN---IEGNHVYLVFEYTTGDASGQNMVTIATHAVFEFIMRHS-----P-VAPVQAFLDGNLSGDKKANSYTLRS--------VRGKKVSAEVHLSAELVKKYLHTTPEQMVQFGQ-MTTVGGAL-SGAIGVNAHYANALAALYIACGQDAACVAESAIGM-----------TRMEIHPH-GGLYASVTLPNLMVGTVGGGTHL-PSQHACLSLMGLAG----QGHARALAEVAAALCLAGELSIVGAFCAG-H-FSRAHHKLA-----------------------------------------------------R
