# Supplementary material for: The mevalonate pathway of isoprenoid biosynthesis supports metabolic flexibility in Mycobacterium marinum
Source: J Bacteriol. 2025 Oct 30;207(11):e00287-25. doi: 10.1128/jb.00287-25 (PMC12632256; doi:10.1128/jb.00287-25)
Supplement: Supplemental figures — Figures S1 to S5. [file jb.00287-25-s0004.pdf]

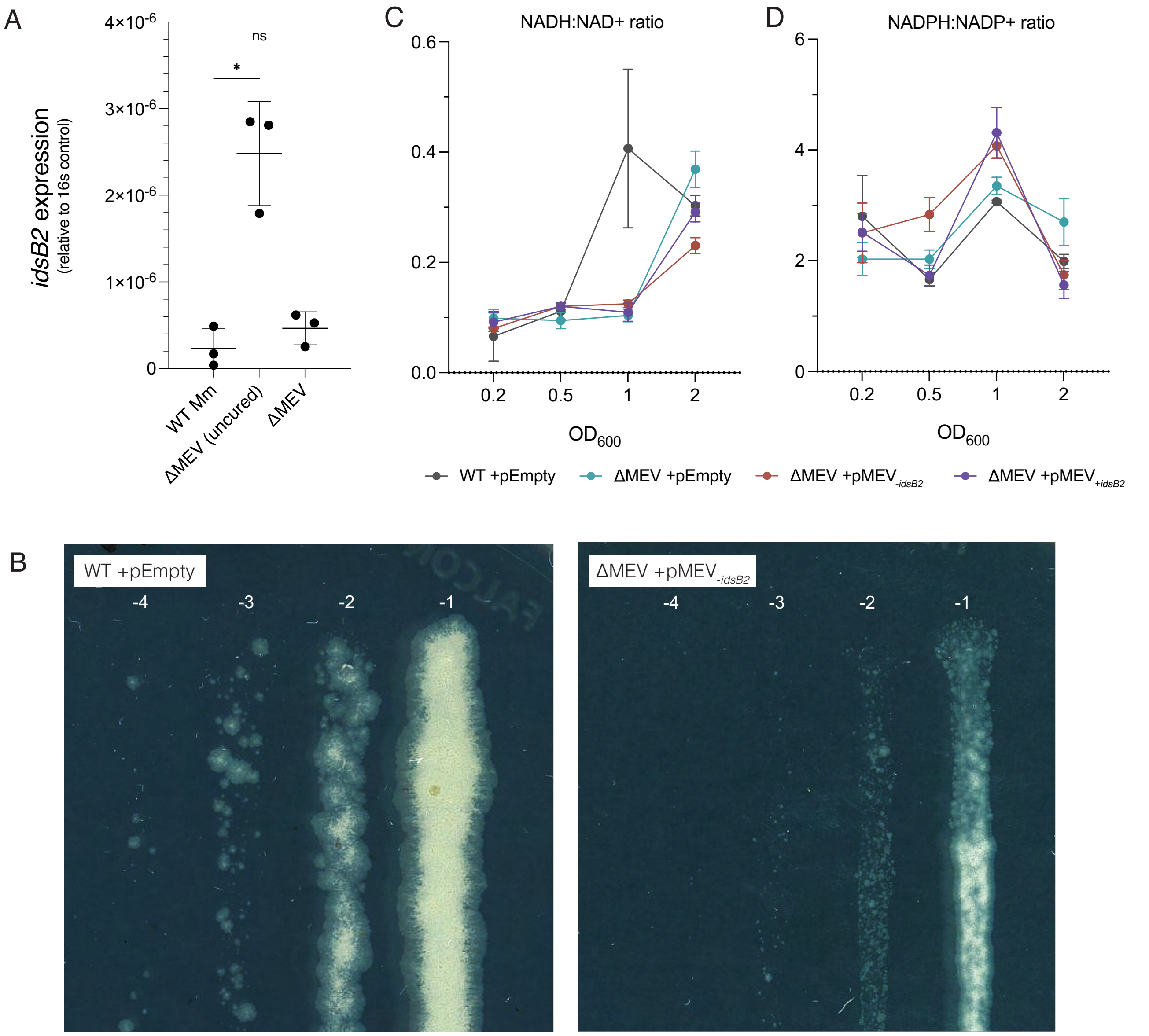

Figure S1: Initial strain characterization.

A. Relative expression of *idsB2* in WT, ORBIT-integrated ΔMEV, and cured ΔMEV. Integration of the ORBIT plasmid to generate the ΔMEV knockout strain caused strong polar effects on the expression of the downstream gene *idsB2* (p=0.0208; one-way mixed effects ANOVA + Dunnett's multiple comparison). Upon excising the integrated plasmid from this strain, *idsB2* levels returned to WT (p=n.s.). Data shown are relative expression of *idsB2* compared to 16s mRNA. N=3 per strain.

B. ΔMEV +pMEV$_{-idsB2}$ forms small colonies. Mid-log cultures of WT and ΔMEV +pMEV$_{-idsB2}$ were serially diluted and plated on 7H10 agar. Plates were incubated at 30°C for five days prior to imaging.

C, D. Dinucleotide cofactors to assess cellular redox states. NADH:NAD+ **(C)** and NADPH:NADP+ **(D)** ratios across growth phases. NADH:NAD+ ratio is inversely correlated with AEC ratio. Shown is mean ± SD. N=3 biological replicates per strain.

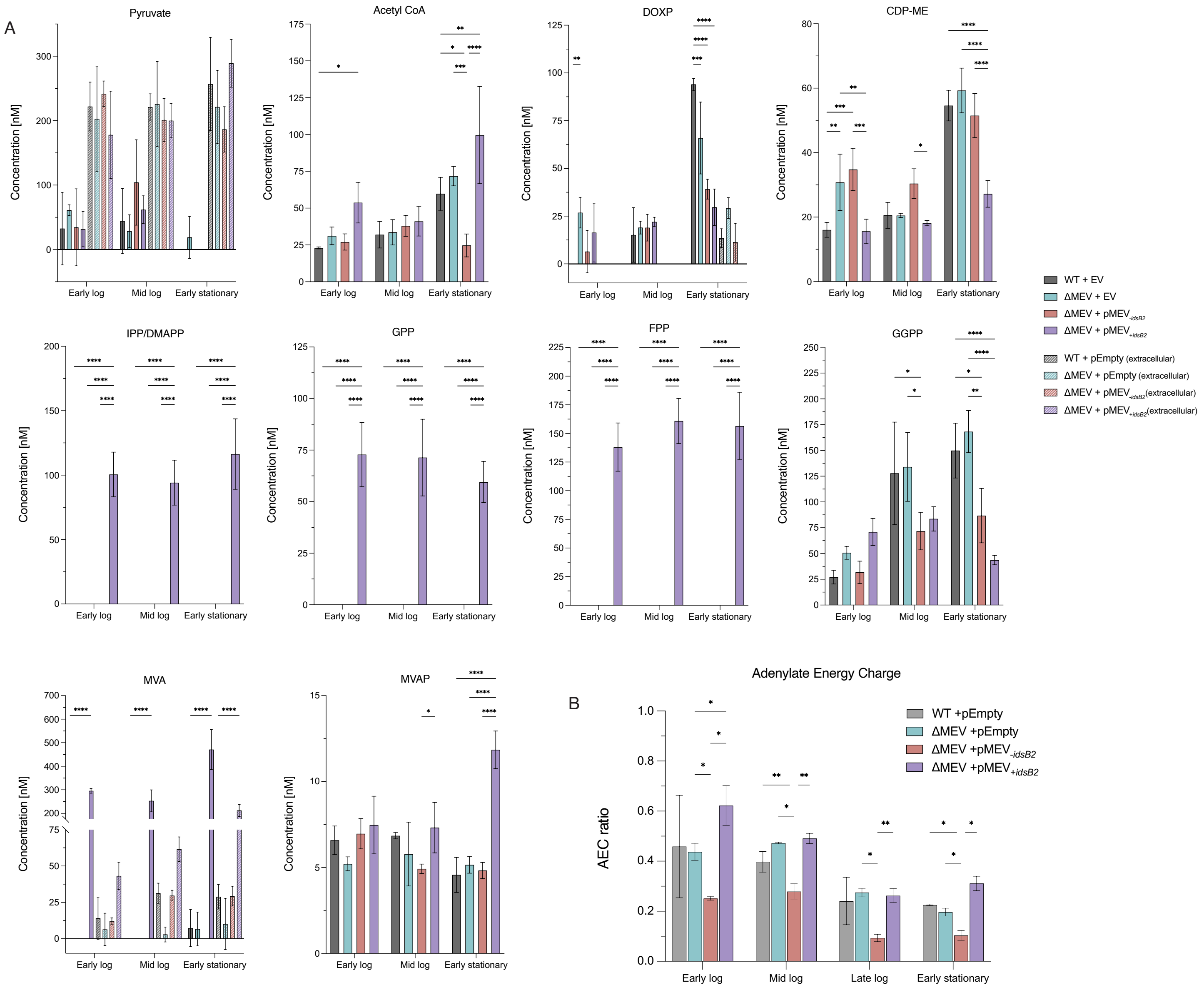

Figure S2: Statistical analyses of metabolomics and survival data.
A. Metabolic differences among strains. Data from figure 4A were visualized via bar graph and analyzed via two-way ANOVA followed by Tukey's multiple comparisons test. *$p \leq 0.05$, **$p \leq 0.01$, ***$p \leq 0.001$, ****$p < 0.0001$. Extracellular concentrations of metabolites are included in panels only when detected.
B. Adenylate energy charge ratio differences among strains. Data from figure 4D were visualized via bar graph and analyzed via two-way ANOVA followed by Tukey's multiple comparisons test. *$p \leq 0.05$, **$p \leq 0.01$.

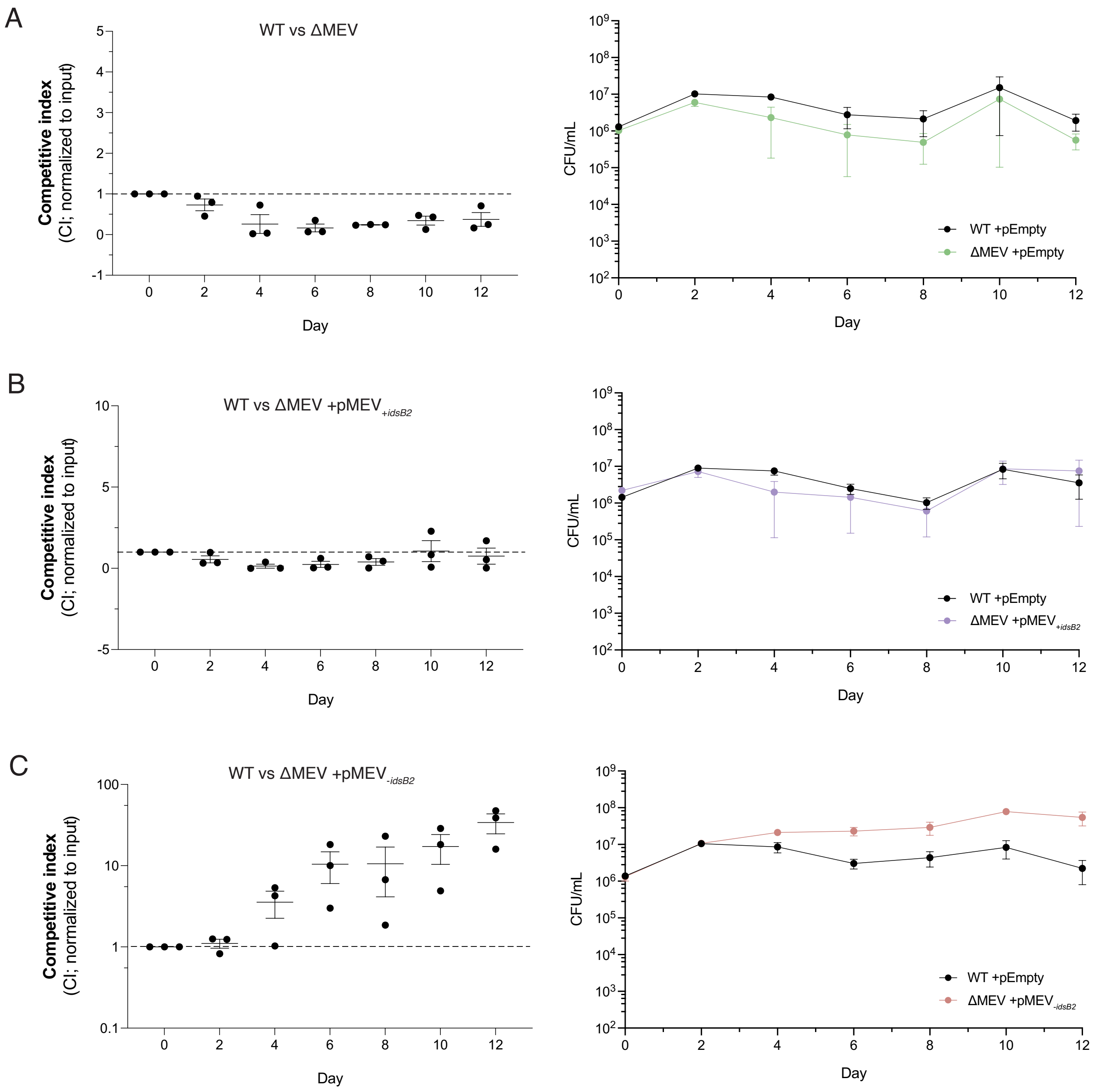

Figure S3: Similarly to normoxic competition, ΔMEV has a competitive defect while ΔMEV +pMEV$_{-idsB2}$ outcompetes WT. The strains were directly competed as in figure 5 (normoxic competition) but in sealed tubes per the Wayne model. As observed in normoxia, ΔMEV had a competitive defect (**A**) which can be rescued by complementation with MEV$_{+idsB2}$ (**B**), but complementation with MEV$_{-idsB2}$ strongly outcompeted WT (**C**). Cultures were plated every other day for twelve days on selective media. CFU were counted to calculate competitive index (CI) normalized to input.

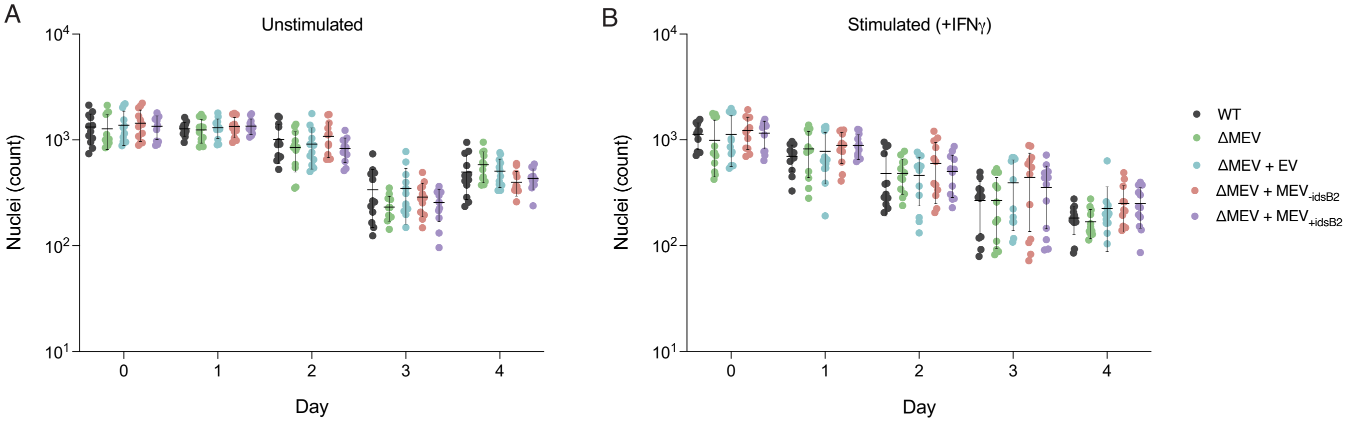

Figure S4: Macrophage cell death is not significantly different between the strains. Wild-type bone marrow-derived macrophages (BMMs) were infected at an MOI of 1 as previously described, either without (**A**) or with (**B**) IFNγ. Plates were imaged on a PerkinElmer OperaPhenix confocal microscope and number of nuclei were quantified via the Harmony software. Data plotted are mean nuclei count ± SD. N=3 biological replicates; 4 technical replicates per biological replicate.

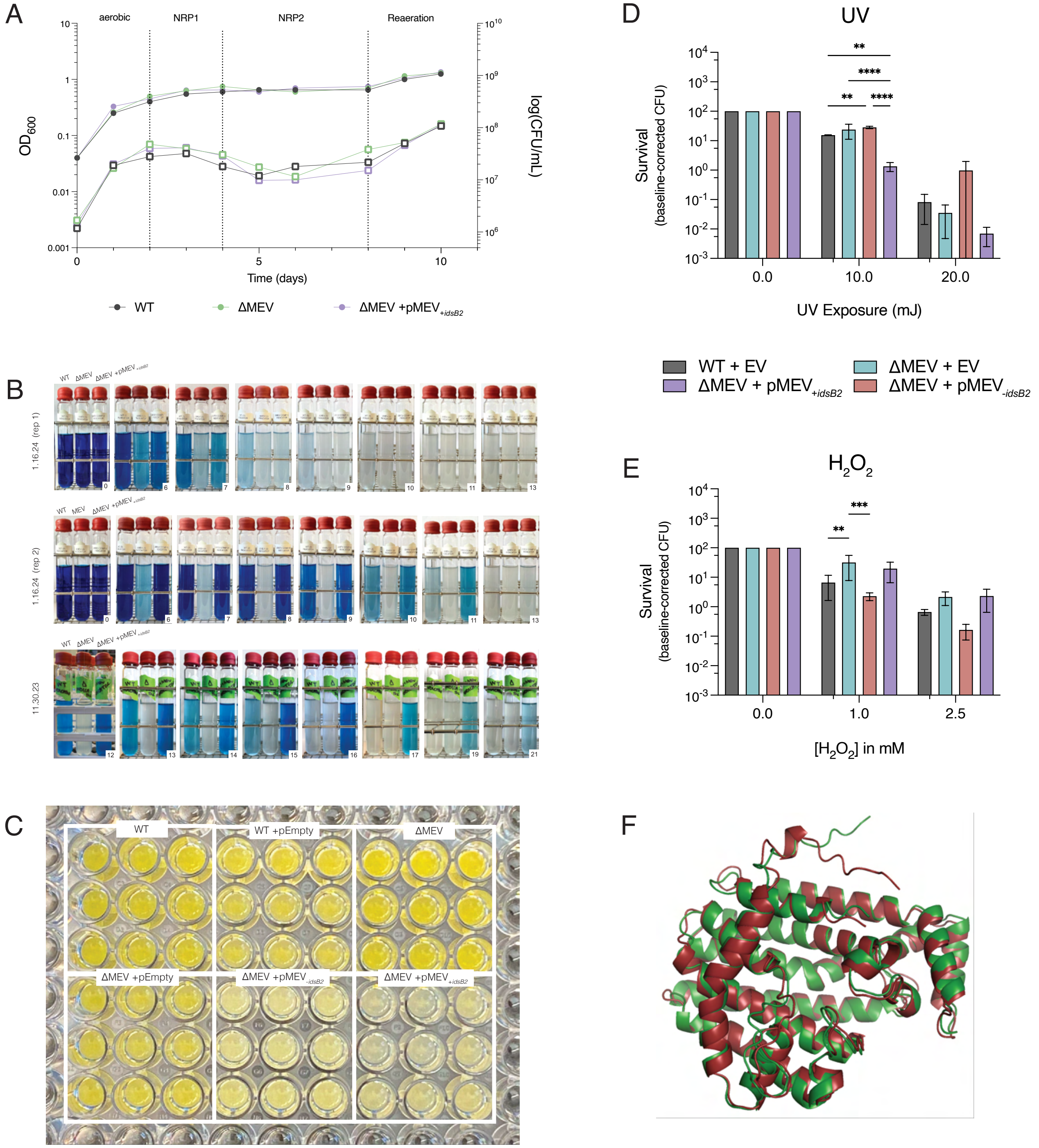

Figure S5: Environmental stress and response.

A. There is no significant difference in OD or CFU throughout hypoxia among the strains. Strains were slowly induced for hypoxia using the Wayne model. Cultures were started at OD_{600} 0.05 in glass tubes with 8mm stir bars to a headspace ratio of 0.5. Tubes were sealed with a rubber stopper and incubated at 30°C with stirring. OD_{600} and CFU samples were taken daily for a minimum of 10 days. To avoid reaeration during the time course, one tube was designated per timepoint.

B. MmΔMEV decolorizes quickly in hypoxia, while the complemented strain decolourizes more slowly compared to WT. WT Mm (left), ΔMEV (middle), and ΔMEV +pMEV$_{+idsB2}$ (right) were slowly induced for

hypoxia as described above. Aeration of the cultures was visualized with the addition of methylene blue (25 µg/mL). Three biological replicates are shown by row. Days after induction of hypoxia are labeled in the bottom right corner.

C. **Complementation strains produce less β-carotene over eight days of growth.** Strains were grown for eight days and OD_{600} was measured to generate the growth curve in Figure 3C. Following this period, the growth plate was left at room temperature in light for 2 days to develop prior to imaging.

D, E. Statistical analyses of UV and H_2O_2 differences among strains. Data from figures 7B and 7C were visualized via bar graph and analyzed via two-way ANOVA followed by Tukey's multiple comparisons test. $^{**}p \leq 0.01$, $^{***}p \leq 0.001$, $^{****}p < 0.0001$.

F. **IdsB2 has high predicted structural homology to IdsB in *M. tuberculosis*.** Shown are the aligned predicted structures of Mm IdsB2 (green) and *M. tuberculosis* IdsB (red). RMSD = 0.822 Å.
